# *Aggregatibacter* *aphrophilus* T6SS Effectors in Host–Bacterial Interactions

**DOI:** 10.1177/00220345251337745

**Published:** 2025-06-26

**Authors:** K. Bao, J. Oscarsson, P. Gehring, J. Grossmann, G.N. Belibasakis, N. Bostanci

**Affiliations:** 1 Division of Oral Health and Periodontology, Department of Dental Medicine, Karolinska Institutet, Stockholm, Sweden; 2 Oral Microbiology, Department of Odontology, Umeå University, Umeå, Sweden; 3 Functional Genomics Center Zurich, ETH Zurich and University of Zurich, Zurich, Switzerland; 4 Swiss Institute of Bioinformatics (SIB) Quartier Sorge-Batiment Amphipole, 1015 Lausanne, Switzerland

**Keywords:** type VI secretion system, type VI secretion system effectors, *Aggregatibacter actinomycetemcomitans*, dental plaque, biofilm, phospholipase D

## Abstract

*Aggregatibacter aphrophilus* is the only known oral bacterium with a functional type VI secretion system (T6SS) that acts against *Aggregatibacter actinomycetemcomitans*. Bacteria use the T6SS to deliver toxic effectors into bacterial or eukaryotic cells during interbacterial competition or host colonization. To date, the T6SS of *A. aphrophilus* has not been demonstrated to participate in antieukaryotic activity, nor have the cytotoxic effectors involved been identified. Here, we identified 2 T6SS effectors in *A. aphrophilus*, a glycosyl hydrolase (Glh) and a phospholipase D (Tle5), which, upon inactivation of their respective genes, together resulted in abolished T6SS activity against *A. actinomycetemcomitans* in multispecies biofilms. Next, we probed the role of the 2 T6SS effectors in host–cell interactions using gingival keratinocytes. Interestingly, although neither of the effectors appeared to contribute to the acute inflammatory response directly, the co-presence of both species reduced the inflammatory effect, likely due to the T6SS-dependent elimination of *A. actinomycetemcomitans*, hence decreasing the bacterial abundance. This reduction was not observed using *A. aphrophilus* mutants lacking the effectors or the T6SS “tube” core protein, hemolysin co-regulated protein (Hcp). Here, we show that the T6SS effectors in *A. aphrophilus* have distinct functions in eukaryotic versus bacterial cell interactions. Hence, these T6SS effectors may represent novel mechanisms of interaction between bacteria and the oral–mucosal barrier, offering potential therapeutic targets for managing periodontal pathogens.

## Introduction

Oral bacteria live within a multispecies, spatially structured biofilm, where they must compete for limited growing space and nutrients (Lamont et al. 2018). Several major competitive mechanisms are wildly adopted by oral bacteria to defend against eukaryotic cells and other bacterial species. One of the most sophisticated mechanisums is the type VI secretion system (T6SS), a contractile nanomachine that injects a needle into neighboring bacteria to deliver toxic proteins, known as “effectors” (Shneider et al. 2013). T6SS effector proteins are injected into neighboring cells as a “bullet,” where they target cell wall components such as peptidoglycan (Whitney et al. 2013) and membrane phospholipids (Russell et al. 2013). Interestingly, although the T6SS is well-known for targeting other bacteria, it was initially discovered for its antieukaryotic activity, disrupting host cells (Pukatzki et al. 2006). Among potential T6SS effector proteins, an ≈80-kDa phospholipase D (Tle5), using *Pseudomonas aeruginosa* as a model organism, has been shown to play a significant role in T6SS-mediated antagonism by targeting membrane phospholipids (Russell et al. 2013), and a glycoside hydrolase (Glh) of *P. aeruginosa* was found to degrade the glycan backbone of cell walls (Whitney et al. 2013).

Although the T6SS is estimated to be present in about 25% of gram-negative bacterial species (Russell et al. 2014; Cianfanelli et al. 2016; Singh and Kumari 2023), *Aggregatibacter aphrophilus* is the only known oral bacterium with a functional T6SS that targets *Aggregatibacter actinomycetemcomitans*, as we recently reported, also assessing mutants lacking the T6SS “tube” core protein, hemolysin co-regulated protein (Hcp), which were deficient in T6SS activity (Oscarsson et al. 2024a, 2024b). Comparative genome analyses have revealed the presence of T6SS in oral *Campylobacter* and *Prevotella* spp. (Ibrahim et al. 2017; Hughes Lago et al. 2024). *A. aphrophilus*, although capable of causing infectious endocarditis and brain abscesses (Kommedal et al. 2014; Nørskov-Lauritsen 2014), is generally considered a symbiont in the oral cavity and is frequently found in periodontally healthy individuals (Tempro and Slots 1986). In contrast, despite their close relationship and sharing of approximately 80% of their genetic content, *A. actinomycetemcomitans* is strongly associated with aggressive forms of periodontitis in young individuals (Belibasakis et al. 2019; Ozuna et al. 2022). Interestingly, the 2 species rarely coexist in a given oral niche, which suggests a competitive relationship (Giacomini et al. 2024). Driven by these observations, we have recently confirmed that the possession of a T6SS provides *A. aphrophilus* with a strong ecological advantage over *A. actinomycetemcomitans* when brought together in the same ecological niche. Whether the T6SS of this species can directly participate in the interaction of *A. aphrophilus* with eukaryotic cells is yet to be identified. To bridge this knowledge gap, we aimed to conduct an in vitro proteome profiling in oral epithelial cells challenged with *A. aphrophilus* alone or in competition with *A. actinomycetemcomitants* and further assessed the involvement of the T6SS effectors Tle5 and Glh by means of deletion mutans.

## Methods

### Construction of *A. aphrophilus glh* and *tle5* Mutants

*A. aphrophilus* strain HK83 (CCUG 49494), originally isolated from saliva (Nørskov-Lauritsen and Kilian 2006), was previously modified to a V factor–independent growth phenotype (Lindholm et al. 2019). BLAST searches (Altschul et al. 1997) were conducted against the whole-genome sequence of strain HK83 (GenBank accession GCA_003703745.1) to obtain the sequences of the genes encoding Tle5 and Glh, respectively. Novel mutants *A. aphrophilus tle5::spe* (HK83 *Δtle5*), *glh::kan* [Spe^r^, Km^r^](HK83 *Δglh*), and *tle5*::*spe*, *glh*::*kan* [Spe^r^, Km^r^] (HK83 *Δtle5 Δglh*) were generated in the present study using a polymerase chain reaction (PCR)–based gene replacement approach as applied to the *hcp* gene of HK83 in our recent work (i.e., strain HK83 *hcp::kan* [Km^r^]) (Oscarsson et al. 2024a, 2024b). Sequences of the oligonucleotide primers used for amplifying flanking sequences to the respective target genes for mutant construction and protein gene database entries are listed in Supplementary Table 1. Confirmation of the *glh* and *tle5* allelic replacements and the orientation of the inserted resistance cassette were done by PCR, using the same approach as described for the *hcp* gene replacement (Oscarsson et al. 2024a, 2024b). In brief, we used a target gene–specific F1 and R2 oligonucleotide primer (Supplementary Table 1), respectively, in combination with a primer specific for the appropriate antibiotic determinant, that is, spectinomycin (Spe1: 5′-CCACTCTCAACTCCTGATCC-3′) or kanamycin (H7R: 5′- GGACGGCGGCTTTGTTGAATAAATCG-3′). The *A. aphrophilus* strains were cultured at 37 °C in 5% CO_2_ on blood agar (5% defibrinated horse blood, 5 mg hemin/L, 10 mg vitamin K/L, Columbia agar base) routinely or in transformation experiments on trypticase soy broth (TSB) agar (0.1% yeast extract, 5% heat-inactivated horse serum, and 1.5% TSB agar) (Becton, Dickinson and Company). For selection, 100 μg/mL kanamycin or spectinomycin was added to the growth media when required.

### Multispecies Biofilm Formation and Harvesting

An in vitro biofilm, containing *A. actinomycetemcomitans* strain JP2 (OMZ 295), *Actinomyces oris* (OMZ 745), *Candida albicans* (OMZ 110), *Fusobacterium nucleatum subsp. nucleatum* KP-F2 (OMZ 598), *Streptococcus oralis* SK248 (OMZ 607), *Streptococcus mutans* UA159 (OMZ 918), and *V. dispar* ATCC 17748T (OMZ 493) with or without *A. aphrophilus* strains (HK83, HK83 *Δhcp*, HK83 *Δtle5 Δglh*) was cultivated on the hydroxyapatite disk and anaerobically incubated for 64 h in a growth medium consisting of 60% saliva, 10% human serum, and 30% modified fluid universal medium as previously described (Oscarsson et al. 2024a, 2024b). The number of the bacteria was quantified by colony-forming unit (CFU). Specifically, serial dilutions of suspended biofilm bacteria were prepared in 0.9% NaCl, and 50-µL aliquots were plated on selective agars: Columbia blood agar (Oxoid, + 5% whole human blood) for total CFU, *A. oris*, *A. aphrophilus*, and *V. dispar*; mitis salivarius agar (Difco) + 1 mL of 1% sodium tellurite solution) for *S. oralis*; fastidious anaerobe agar (Lab M, Potters Bar) with erythromycin (1 mg/L), vancomycin (4 mg/L), and norfloxacin (1 mg/L) for *F. nucleatum*; Biggy agar (Difco) for *Candida albicans*; and Aac-selective agar (Oxoid) + 5% horse serum, vancomycin (5 mg/L), bacitracin (50,000 International units per liter (IU/L)), and 5% human blood for *A. actinomycetemcomitans*.

### Co-culture of Epithelial Cells with *A. actinomycetemcomitans* and/or *A. aphrophilus*

Immortalized human gingival epithelium keratinocytes (HGEK-16) (Bao et al. 2014) were seeded in 24-well plates (4.5 × 10^4^ cells/well) and cultured overnight in defined keratinocyte serum-free medium (Invitrogen) at 37 °C, 5% CO_2_. After washing, cells were infected with wild-type *A. aphrophilus* HK83, *A. actinomycetemcomitans* JP2, or mutant strains (HK83 *Δhcp*, HK83 *Δtle5 Δglh*) alone or in combination (*A. aphrophilus:A. actinomycetemcomitans* in 3:1 ratio) at a multiplicity of infection of 100. Seven co-culture groups were tested, each with 6 replicates. Plates were centrifuged (5 min, 1,500 rpm) to engage bacteria and incubated for 24 h at 37 °C, 5% CO_2_.

### Protein Extraction and Cleanup

Co-cultured cells were washed with ice-cold phosphate-buffered saline (PBS) 3 times, lysed in lysis buffer from the iST sample, and then removed by cell scraper. Fifty micrograms of cured extracted proteins was collected and processed following the manufactory protocol of the in-StageTip (iST) kit (PreOmic). Extracted peptides were dried in a SpeedVac (Thermo Savant SPD121P, Thermo Scientific) and stored at −20 °C.

### Liquid Chromatography Tandem Mass Spectrometry Analysis and Protein Quantification

Peptides were analyzed using an Orbitrap Fusion mass spectrometer (Thermo Fisher Scientific), and label-free quantification was performed with Progenesis QI as described previously (Oscarsson et al. 2024a, 2024b). Protein identifications were conducted using the Mascot (version 2.4.1, Matrix Science) against an in-house database downloaded from Uniport (Supplementary Table 2). Proteins with a fold change >2 and a *t*-test *P* value <0.05 were considered regulated.

### Data Clustering and Functional Analysis

Quantified protein data were analyzed using R software (Quantable package, https://cran.r-project.org/web/packages/quantable/index.html) for unsupervised clustering and heat maps, whereas principal component analysis (PCA) was generated with *ggplot*. Regulated human proteins were further annotated using WebGestalt for gene ontology (GO) enrichment, specifically “Biological Process noRedundant” (GO-BPnR), against all identified proteins (Supplementary Table 3).

## Results

### Identification of T6SS Effectors Glh and Tle5 in *A. aphrophilus* Strain HK83

Blast search of the genome of HK83 revealed that this strain encodes a phospholipase of the Tle5 family (GenBank: RMW89110.1; database name phospholipase) with a length of 719 amino acids (Supplementary Table 1). This protein displays approximately 30% amino acid identity with the type VI secretion system-dependent phospholipase D effector PldB of *Pseudomonas aeruginosa* (GenBank: MBF3156171.1). As a hallmark of this family of proteins, the *A. aphrophilus* protein contains an HxKxxxxD amino acid motif (Uesugi and Hatanaka 2009). Blast search also revealed that strain HK83 encodes a glycoside hydrolase (Glh; GenBank: RMW80835.1; “hypothetical protein”; and GenBank WP_259642111; “glycoside hydrolase family 19 protein”), with a length of 668 amino acids. This protein exhibits partial amino acid identity (approximately 36%) to the Glh family 19 protein of *P. aeruginosa* (GeneBank: HCR1337642.1). The observation that at least the Tle5 protein of HK83 is encoded directly downstream of a gene encoding a type VI secretion-related VirG protein (type VI secretion system Vgr family protein; GenBank: WP_114975745) further prompted us to conduct genetic analysis to assess a potential role of Glh and Tle5 in the T6SS-dependent antagonism against *A. actinomycetemcomitans*. For this, derivatives with allelic replacements of these genes as single and double mutants were subsequently generated in strain HK83.

### Role of Glh and Tle5 in T6SS-Dependent Killing of *A. actinomycetemcomitans* in Multispecies Biofilms

**Figure 1.**
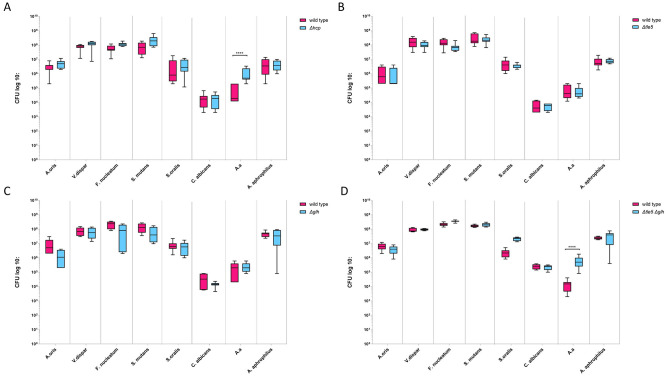
Quantitative composition of multiple-species biofilms. The relative cell abundances of all species in the multispecies biofilms containing either *A. aphrophilus* HK 83 wild type or *Δhcp* (**A**), *Δtle5* (**B**), *Δglh* (**C**), or *Δtle5 Δglh* (**D**) were measured. Numbers of each strain were counted by colony-forming units (CFUs), and data were plotted on a logarithmic scale. Asterisks (****) indicate significant differences (*P* ≤ 0.0001) between the groups. A. a., *A. actinomycetemcomitans*.

We next evaluated the T6SS-mediated competitive potency of wild-type or mutant *A. aphrophilus* HK83 strains against the *A. actinomycetemcomitans* JP2 strain within multispecies biofilms. Consistent with previous findings, the presence of wild-type *A. aphrophilus* significantly reduced the abundance of *A. actinomycetemcomitans* compared with biofilms lacking *A. aphrophilus*, a reduction that was abolished in T6SS-deficient strains (*Δhcp* mutant) (Fig. 1A). Interestingly, deletion of Tle5 (Fig. 1B) or Glh (Fig. 1C) alone in *A. aphrophilus* did not significantly diminish *A. actinomycetemcomitans* killing. However, the simultaneous deletion of both effectors led to a resurgence in *A. actinomycetemcomitans* numbers (Fig. 1D). Importantly, neither *A. aphrophilus* nor its derivative mutants significantly affected the growth of other species present in the biofilm, underscoring the specificity of the T6SS effect.

### Role T6SS-Dependent Killing of *A. actinomycetemcomitans* in Epithelial Cell Responses

**Figure 2.**
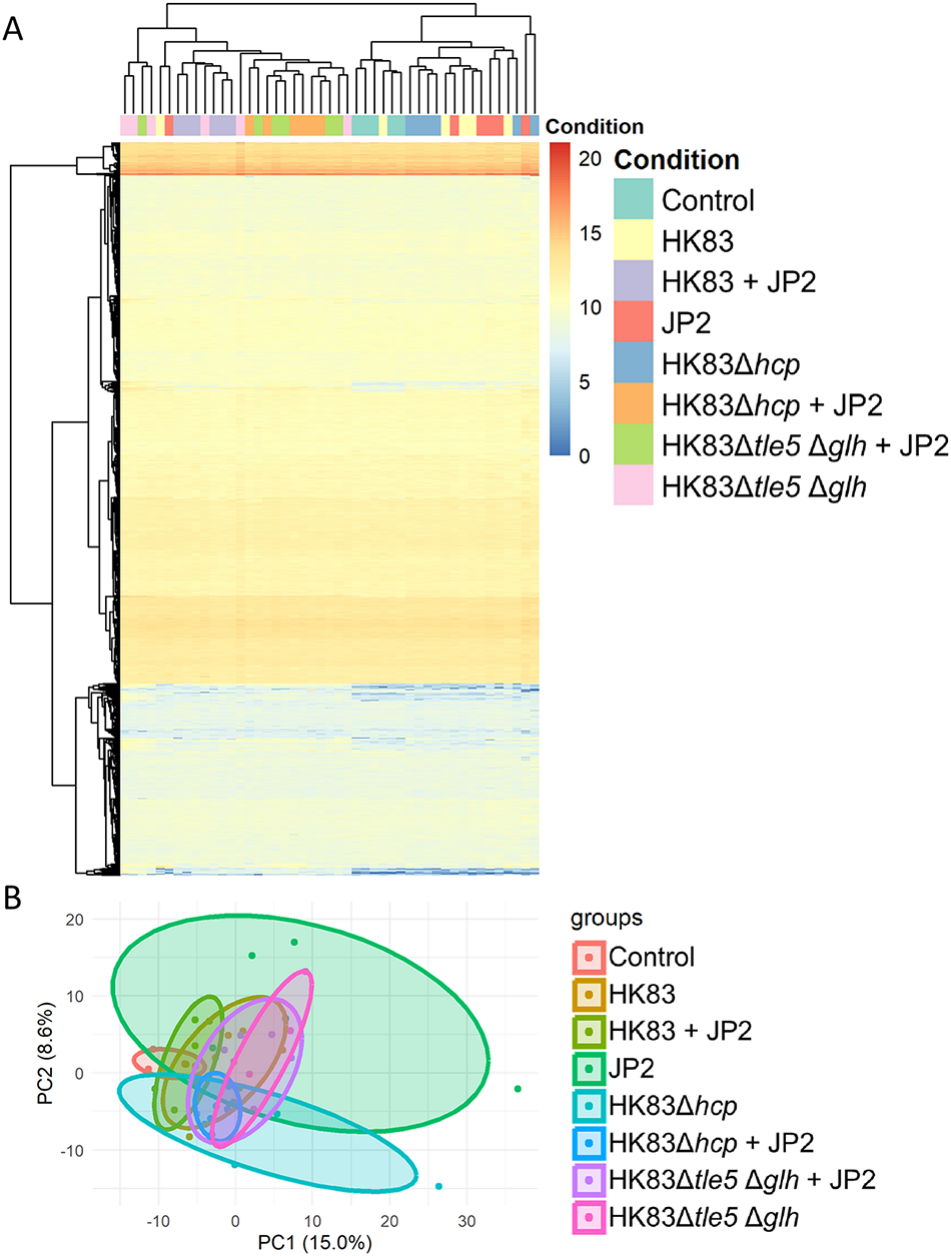
Overview of human proteins quantified from each sample. (**A**) Heat map displaying the normalized abundance of quantified proteins. The colors in the map represent the value of the hyperbolic arcsine transformed normalized abundance plus 1 for individual proteins (represented by a single row) within each experimental sample (represented by a single column). Expression values are shown on a color scale, with higher values in red and lower values in blue. The 8 groups of the immortalized epithelial cell line, either challenged with or without the bacterial strains, are as follows: control without bacterial challenge (control, *n* = 5), *A. aphrophilus* strain HK83 (HK83, *n* = 6), *A. aphrophilus* strain HK83 *Δhcp* mutant (HK83 *Δhcp*, *n* = 6), *A. aphrophilus* strain HK83 *Δtle5 Δglh* mutant (HK83 *Δtle5 Δglh*, *n* = 6), and *A. actinomycetemcomitans* strain JP2 (JP2, *n* = 6). These are color coded. (**B**) Principal component analysis (PCA) of the proteome, clustered based on the inverse hyperbolic sine transformation of the spectrum counts for the identified proteins across the 8 groups for all human proteins.

The T6SS can also deliver effectors that target several cellular processes in eukaryotic cells, thus facilitating pathogenesis. Therefore, we first sought to determine whether the individual effectors or Hcp of *A. aphrophilus* exert any effects on epithelial cells, in addition to their competitive activity toward *A. actinomycetemcomitans*. We used various strains of *A. aphrophilus* together with *A. actinomycetemcomitans* to co-infect the cells: the wild type (HK83 + JP2, *n* = 6), the T6SS-deficient strain (HK83 *Δhcp* + JP2, *n* = 6), and the double effector mutants (HK83 *Δtle5 Δglh* + JP2, *n* = 6). We also used single inoculations of *A. aphrophilus* to infect the cells: the wild type (HK83, *n* = 6), the *Δhcp* mutant (HK83 *Δhcp*, *n* = 6), the *Δtle5 Δglh* mutant (HK83 *Δtle5 Δglh*, *n* = 6), and *A. actinomycetemcomitans* JP2 (JP2, *n* = 6). Cells cultured alone without any bacterial challenge served as controls. Epithelial cells expressed 3,189 proteins with a protein false discovery rate of 0.284%. After excluding contaminants and decoys, this included 2,942 human proteins, 46 *A. aphrophilus* proteins, 125 *A. actinomycetemcomitans* proteins, and 54 proteins that could not be distinguished between the 2 bacterial species due to shared intergeneric peptides (see deposit data). Nevertheless, because it is difficult to determine whether the detected bacterial proteins originated from within the epithelial cells or were residual contaminants from the co-culture experiment (despite 3 washes with PBS to remove bacteria), only human proteins were analyzed further in this study. Initial analysis using unsupervised clustering (Fig. 2A) and PCA (Fig. 2B) based on human proteome expression failed to separate the groups. We then focused on regulated proteins (*P* < 0.05, fold change >2, Supplementary Table 4) comparing unchallenged and bacterial-challenged groups to assess inflammatory changes as well as comparing HK83-included groups with the corresponding T6SS- or dual-effector–deficient mutants to evaluate the effects of T6SS on epithelial cells. These analyses are further elaborated below.

#### Inflammatory changes in co-culture groups

**Figure 3.**
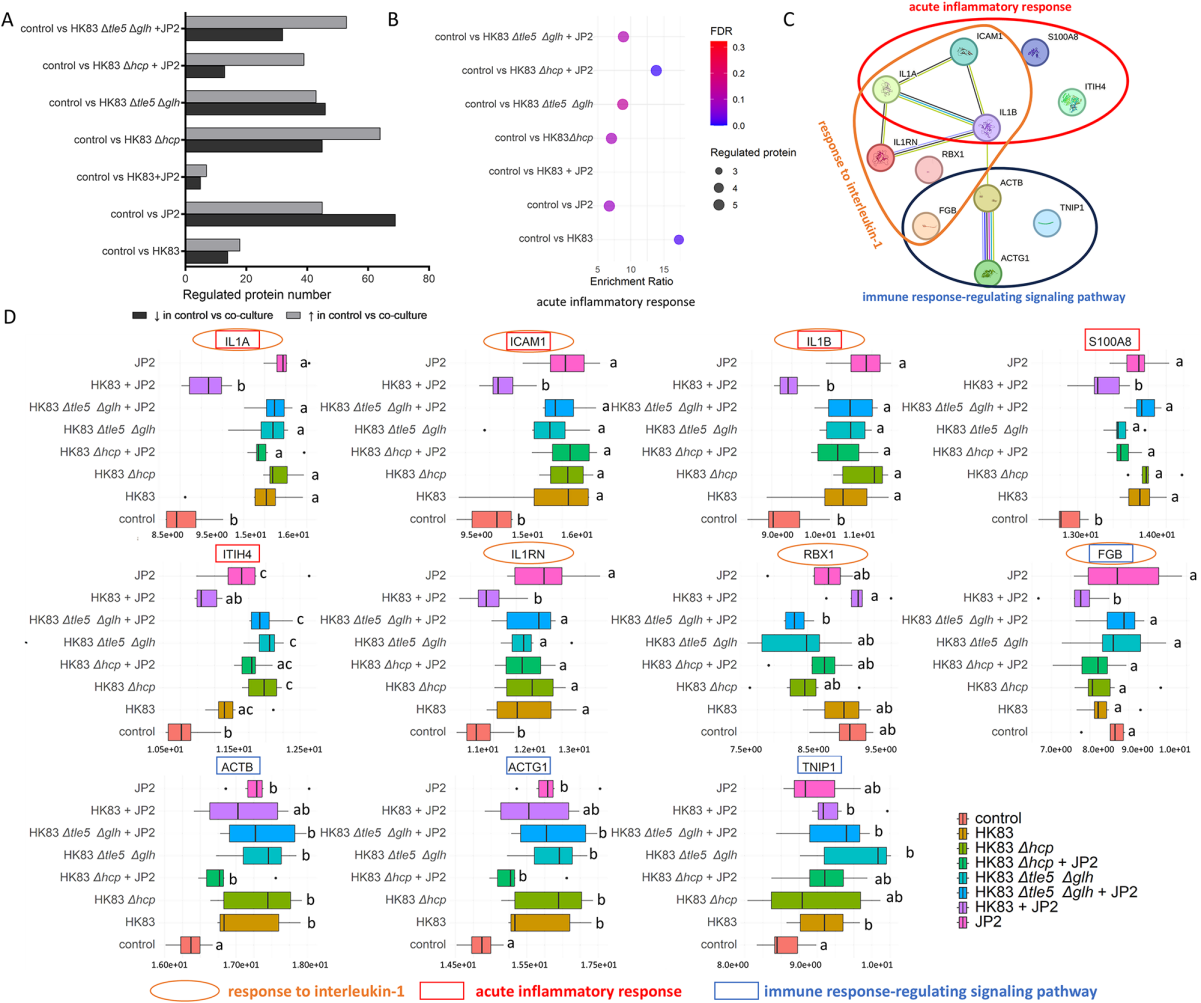
Regulated human proteins in bacterial-challenged epithelial cells compared with unchallenged cells. (**A**) Number of regulated proteins in each comparison. (**B**) Results of the “acute inflammatory response” based on overrepresentation analysis (ORA) enrichment across different comparisons (Supplementary Table 5). (**C**) Protein interactions between regulated proteins contributing to the enrichment of the “acute inflammatory response,” “response to interleukin-1,” and “immune response–regulating signaling pathway.” These interactions were established using STRING 10.5 (Supplementary Table 6), with the highest confidence score (0.9) for proteins involved in the corresponding pathways (highlighted with different circles). (**D**) Expression levels of proteins contributing to the “acute inflammatory response,” response to interleukin-1,” and “immune signaling pathways.” Proteins contributing to the enrichment of these pathways are marked with brown circles, red boxes, and blue boxes, respectively. Protein abundance is shown as hyperbolic arcsine-transformed normalized abundances. Different letters (a, b, or c) on the right of the box plot indicate significant differences (*P* < 0.05, fold change >2) between the compared groups based on the Progenesis QI exported result (Supplementary Table 4).

Compared with control, the HK83 + JP2 combination stimulated the fewest regulated human proteins (12), followed by HK83 (32), HK83 *Δhcp* + JP2 (52), HK83 *Δtle5* Δ*glh* + JP2 (85), HK83 *Δtle5* *Δglh* (89), HK83 *Δhcp* (109), and JP2 (114) (Fig. 3A). Hence, *A. actinomycetemcomitans*, but not *A. aphrophilus* alone, causes a pronounced host response, which is in turn ablated by the co-presence of *A. aphrophilus*.

The “acute inflammatory response” category appeared among the top 10 enriched GO-BPnR functions for nearly all bacterial co-infection–challenge conditions versus control, except in the HK83 + JP2 group (Fig. 3B, Supplementary Table 5). Proteins contributing to this enrichment, including ITIH4, ICAM1, IL1A, IL1B, and S100A8 (Fig. 3C), showed similar abundance in the HK83 + JP2 group compared with control (highlighted in red in Fig. 3D), while their levels were significantly higher in other bacterial challenges. Conversely, the HK83 + JP2 combination elicited the “immune response–regulating signaling pathway,” an immune-related cellular response that was not observed in among other challenge groups (Supplementary Table 5). This pathway involved proteins such as FGB, TNIP1, ACTB, and ACTG1. While most of these proteins were also upregulated in other groups (highlighted in blue, Fig. 3D), their expression levels did not rank among the top 10, due to stronger responses documented in other pathways. Hence, co-infection with HK83 + JP2 triggers a distinct immune-related cellular response, compared with the other groups. The only remaining top immune-related term, “response to interleukin-1,” emerged as an enriched one (Supplementary Table 5), with 4 of 6 contributing proteins also involved in the “acute inflammatory response” and “immune response–regulating signaling pathway” (Fig. 3C). The 2 unique proteins, IL1RN and RBX1, displayed similar expression levels in the HK83 + JP2 group and the untreated control, whereas IL1RN was markedly higher and RBX1 was significantly lower in other groups (highlighted in brown, Fig. 3D), suggesting a less affected interleukin-1 response in the HK83 + JP2 combination compared with the other groups.

In summary, both the regulated protein counts and the enrichment analyses indicate that the co-infection with HK83 + JP2 had the fewest protein expression changes on gingival epithelial cells, likely due to the ability of HK83 to kill JP2. This effect was absent with HK83 strains lacking the core tube protein Hcp or effectors Tle5 and Glh.

#### Impact of T6SS on epithelial cells

**Figure 4.**
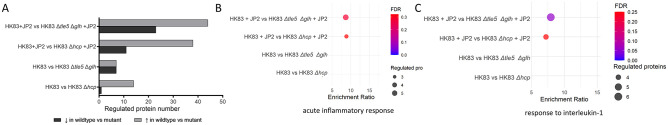
Regulated proteins in epithelial cells challenged by wild-type *A. aphrophilus* and its mutants, alone and in combination. (**A**) Number of regulated human proteins in each comparison. (**B**) Acute inflammatory response results based on overrepresentation analysis (ORA) enrichment across different comparisons (Supplementary Table 5). (**C**) Response to interleukin-1 results based on ORA enrichment across different comparisons.

In the absence of JP2, cells monoinfected with *A. aphrophilus* wild type showed minimal protein regulation compared with its mutants: 15 in HK83 versus HK83 *Δhcp* and 14 in HK83 versus HK83 *Δtle5* Δ*glh* (Fig. 4A). Overrepresentation analysis showed no enrichment for “acute inflammatory response,” “response to interleukin-1” (Fig. 4B and C), or other immune-related functions based on these proteins (Supplementary Table 5), indicating a low inflammatory-inducing potential by *A. aphrophilus*, in line with its commensal symbiont ecological profile. However, when the cells were co-infected with *A. aphrophilus* and JP2, more proteins were regulated (49 in HK83 + JP2 vs. HK83 *Δhcp* + JP2, 67 in HK83 + JP2 vs. HK83 *Δtle5* Δ*glh* + JP2) (Fig. 4A), including 4 of the 5 proteins involved in the acute inflammatory response (except from S100A8) (Fig. 4B) and all 6 proteins contributing to “response to interleukin-1” (Fig. 4C). This indicates that neither the core tube protein Hcp nor the effectors Tle5 and Glh appear to have an effect on the key inflammatory pathways of the epithelial cells.

## Discussion

Previously, we discovered that the oral symbiont *A. aphrophilus* possesses a T6SS, which it uses to eliminate its close phylogenetic relative, the oral pathobiont *A. actinomycetemcomitans* (Oscarsson et al. 2024a). In the present work, we further investigated how this interspecies competition may regulate host cell responses in the case of co-infection, highlighting further the role of the T6SS and its 2 effector molecules, Tle5 and Glh. Notably, only the simultaneous deletion of both effectors impaired the ability of *A. aphrophilus* to kill *A. actinomycetemcomitans* within a multispecies biofilm, suggesting that these proteins may act synergistically to enhance targeted bacterial killing. Using quantitative proteomics, we investigated the response of gingival epithelial cells exposed to *A. actinomycetemcomitans* and *A. aphrophilus* in detail. When co-infecting epithelial cells using either wild-type or dual-effector–deficient mutants of *A. aphrophilus* together with *A. actinomycetemcomitans JP2*, we demonstrated the absence of an acute host inflammatory response of the host cells, independently of the T6SS-dependent pathobiont killing.

*A. actinomycetemcomitans* is known to disrupt the gingival epithelial barrier via its virulence factors (Belibasakis et al. 2019) and induce the production of proinflammatory cytokines such as interleukin (IL)–1β (Uchida et al. 2001), which was also observed in our present work. In contrast, *A. aphrophilus*, which is typically considered as an oral symbiont, associated with occasional cases of endocarditis and brain abscesses (Kommedal et al. 2014; Nørskov-Lauritsen 2014), and its epithelial-level host response has not been thoroughly studied. Nevertheless, due to its close genetic relationship with *A. actinomycetemcomitans*, sharing approximately 80% gene content (Kittichotirat et al. 2016), it is not surprising that *A. aphrophilus* also elevates IL-1β. Functional analysis revealed that both bacteria promoted the enrichment of proteins involved in the acute inflammatory response, including elevating IL-1α, IL-RN, and S100A8, which are key players in the immune response during periodontitis. As innate immune effectors in experimental periodontitis (Johnstone et al. 2021), S100A8 levels were elevated in the presence of bacteria in the junctional epithelium (Nishii et al. 2013). Interestingly, 2 other cytokines regulated in our study, IL-1α (Bando et al. 2013) and IL-RN (Sorenson et al. 2012), controlled S100A8/A9-dependent keratinocyte resistance to bacterial invasion. The interaction between these elevated proteins, as shown by our protein–protein interaction analysis, suggests a dynamic host immune response triggered by the presence of both bacteria. Notably, when epithelial cells were co-infected with both bacteria, the expression of these cytokines was almost identical to the unchallenged cells, and this was abolished in T6SS- or dual-effector–deficient mutants. This suggests that the killing of *A. actinomycetemcomitans* by *A. aphrophilus* occurs in a T6SS-dependent manner, and this phenomenon ablates the deleterious effect of *A. actinomycetemcomitans* on the epithelial host response. Further analysis revealed that the T6SS of *A. aphrophilus* appeared to have a very low impact on gingival epithelial cells, with fewer than 0.5% of human proteins (none of them either with an apparent immune defense–associated function as known) being differentially regulated when comparing those co-infected with wild-type *A. aphrophilus* or their mutants. While the T6SS is well known for targeting neighboring bacterial cells (Ho et al. 2014), it can also target eukaryotic cells through the delivery of toxic effector proteins. For instance, *P. aeruginosa* utilizes the T6SS effector Tel5a (PldA) to establish persistent infections in a rat model (Wilderman et al. 2001), and *Aeromonas hydrophila* uses its T6SS to disrupt the actin cytoskeleton via the VgrG1 effector (Suarez et al. 2010). However, such cytotoxic effects do not appear to occur in *A. aphrophilus*, suggesting its T6SS is specialized for bacterial competition rather than eukaryotic cell targeting.

In the natural oral environment, both *A. aphrophilus* and *A. actinomycetemcomitans* exist in dental plaque (Bostanci et al. 2021; Manoil et al. 2024). Therefore, studying these organisms in multispecies biofilms is crucial. We previously demonstrated that *A. aphrophilus* can eliminate *A. actinomycetemcomitans* via its T6SS, a process that was abolished by deleting the gene encoding the T6SS tube protein, Hcp (Oscarsson et al. 2024a, 2024b). In the present work, we detected that simultaneous inactivation of the genes encoding the Tle5 and Glh effectors also impaired this T6SS-dependent killing effect, suggesting potential synergy. To the best of our knowledge, the only study hitherto highlighting synergistic effects among T6SS effectors involves *P. aeruginosa*, where Tse4 acts in concert with other effectors that either compromise the cell wall (Tse1 and Tse3) or inactivate intracellular electron carriers (Tse6) (LaCourse et al. 2018). Given that T6SS effectors act as the “bullets” of this secretion system, it stands to reason that deploying multiple effectors would provide a greater fitness advantage during interbacterial competition, as we observed in this study.

## Conclusion

Our present work offers further insights into the T6SS-dependent antagonistic behavior of *A. aphrophilus* toward *A. actinomycetemcomitans* within biofilms, underscoring the critical role of T6SS and its synergistic effectors in shaping bacterial dynamics. Furthermore, in a host co-infection environment, the direct impact of *A. aphrophilus* T6SS on host epithelial cells was very low, suggesting that this system may primarily mediate interbacterial interactions, rather than host pathogenic mechanisms. Understanding these mechanisms illuminates how bacterial competition queues may play on the host response, to eventually permit or prevent the downstream pathogenic effects that lead to disease. Future research can be directed to explore the broader implications of T6SS effectors in complex microbial communities and investigate whether potential effector synergy may occur in other T6SS-dependent symbiont-pathobiont interactions. This knowledge could inform the development of new therapeutic strategies targeting bacterial competition in infectious diseases.

## Author Contributions

K. Bao, contributed to data acquisition, analysis, and interpretation, drafted and critically revised the manuscript; J. Oscarsson, contributed to conception, design, data acquisition, analysis, and interpretation, drafted and critically revised the manuscript; P. Gehring, contributed to data acquisition, critically revised the manuscript; J. Grossmann, contributed to data analysis, critically revised the manuscript; G.N. Belibasakis, N. Bostanci, contributed to conception and design, drafted and critically revised the manuscript. All authors gave final approval and agree to be accountable for all aspects of the work.

## Supplemental Material

sj-docx-1-jdr-10.1177_00220345251337745 – Supplemental material for Aggregatibacteraphrophilus T6SS Effectors in Host–Bacterial Interactions Supplemental material, sj-docx-1-jdr-10.1177_00220345251337745 for Aggregatibacteraphrophilus T6SS Effectors in Host–Bacterial Interactions by K. Bao, J. Oscarsson, P. Gehring, J. Grossmann, G.N. Belibasakis and N. Bostanci in Journal of Dental Research
