# Supplementary material for: Aggregatibacter aphrophilus T6SS Effectors in Host–Bacterial Interactions
Source: J Dent Res. 2025 Jun 26;104(13):1487–94. doi: 10.1177/00220345251337745 (PMC12578953; doi:10.1177/00220345251337745)
Supplement: sj-docx-1-jdr-10.1177_00220345251337745 – Supplemental material for Aggregatibacteraphrophilus T6SS Effectors in Host–Bacterial Interactions [file sj-docx-1-jdr-10.1177_00220345251337745.docx]

***Aggregatibacter aphrohilus* T6SS Effectors in Host-Bacterial Interactions**

Kai Bao^1^, Jan Oscarsson^2^, Peter Gehring^3^, Jonas Grossmann^3,4^, Georgios N. Belibasakis^1^, Nagihan Bostanci^1^

**Affiliations:**

^1^Division of Oral Health and Periodontology, Department of Dental Medicine, Karolinska Institutet, Stockholm, Sweden

^2^Oral Microbiology, Department of Odontology, Umeå University, Umeå, Sweden

^3^Functional Genomics Center Zurich, ETH Zurich and University of Zurich, Switzerland

^4^Swiss Institute of Bioinformatics (SIB) Quartier Sorge-Batiment Amphipole, 1015 Lausanne, Switzerland

*Correspondence: Dr. Kai Bao, Division of Oral Health and Periodontology, Department of Dental Medicine, Karolinska Institutet, Stockholm, Sweden.

Assoc Prof. Jan Oscarsson, Oral Microbiology, Department of Odontology, Umeå University, Umeå, Sweden.

The abstract word count: 212

The total word count: 3140

Number of references: 35

Keywords: type VI secretion system, type VI secretion system effectors, *Aggregatibacter actinomycetemcomitans*, dental plaque, biofilm

**Supplementary Table 1:** T6SS effector proteins in the *A. aphrophilus* HK83 genome and primers for allelic replacement mutants

| **Target gene** | **Name in GenBank** | **Predicted activity** | **Oligonucleotide1 (F: forward; R: reverse)** | **PCR Product Length (bp)** |
| --- | --- | --- | --- | --- |
| *glh* | RMW80835.1 hypothetical protein DOL88_09385, partial [*Aggregatibacter aphrophilus]* | Belongs to the glycoside hydrolase family 19 (PF00182) | F1: 5’-CTTTAGTATTGGGCGGGGCAGAAC-3’ R1: 5’-CAAAACTCACTACGTCGGATCCGGTATTA-3’ | 1,252 |
|  |  |  | F2: 5’-CTCCCAATAAGGCAGGATCCGGAG-3’ R2: 5’-CTTGCACCGTCTCCCTCTTTAG-3’ | 1,263 |
| *tle5* | RMW89110.1 phospholipase [*Aggregatibacter aphrophilus]* | Belongs to the Tle superfamily, and conserved protein domain family PLN 02270 | F1: 5’-GGGTAGTGCCGGGGTATGTG-3’ R1: 5’-CTTACCTTCTGTTGTCGACATCTGTTG-3’ | 1,497 |
|  |  |  | F2: 5’-CCCACAGTAAGTCGACGTGACTAAA-3’ R2: 5’-GGCACCACCTAAATCAACACCTTC-3’ | 1,403 |

Primers introducing *Bam*HI (GGATCC) and *Sal*I (GTCGAC) restriction sites (sequences in underlined bold) as indicated

**Supplementary Table 2:** Table of Uniprot Proteome identifiers for the in-house database

| Taxonomy | NCBI taxon identifier | Nr. of database | Uniprot proteome identifier |
| --- | --- | --- | --- |
| *Aggregatibacter aphrophilus* | 732 | 4 | UP000066164;UP000253728;UP000253767;UP000003516 |
| *Aggregatibacter actinomycetemcomitans* | 714 | 6 | UP000072236;UP000005508;UP000216955;UP000019758;UP000003665;UP000006254 |
| *Homo sapiens* | 9606 | 1 | UP000005640 |
| all |  | 11 |  |

The database, released 16th January 2019, has 189,485 sequences and 62,718,866 residues.

**Supplementary Table 3:** List of background proteins used for over-representative analysis

| Nr. | geneSymbol | geneName | entrezgene |
| --- | --- | --- | --- |
| 1 | S100A8 | S100 calcium binding protein A8 | 6279 |
| 2 | CSTB | cystatin B | 1476 |
| 3 | HPX | hemopexin | 3263 |
| 4 | SH3PXD2A | SH3 and PX domains 2A | 9644 |
| 5 | HINT1 | histidine triad nucleotide binding protein 1 | 3094 |
| 6 | LRBA | LPS responsive beige-like anchor protein | 987 |
| 7 | PFKM | phosphofructokinase, muscle | 5213 |
| 8 | C7orf50 | chromosome 7 open reading frame 50 | 84310 |
| 9 | WDR33 | WD repeat domain 33 | 55339 |
| 10 | TP53BP1 | tumor protein p53 binding protein 1 | 7158 |
| 11 | FADS2 | fatty acid desaturase 2 | 9415 |
| 12 | DNMT1 | DNA methyltransferase 1 | 1786 |
| 13 | UTP14A | UTP14A, small subunit processome component | 10813 |
| 14 | NUDT5 | nudix hydrolase 5 | 11164 |
| 15 | KYNU | kynureninase | 8942 |
| 16 | KDM1A | lysine demethylase 1A | 23028 |
| 17 | CYP51A1 | cytochrome P450 family 51 subfamily A member 1 | 1595 |
| 18 | SOD2 | superoxide dismutase 2 | 6648 |
| 19 | KRR1 | KRR1, small subunit processome component homolog | 11103 |
| 20 | TAX1BP3 | Tax1 binding protein 3 | 30851 |
| 21 | RRP1B | ribosomal RNA processing 1B | 23076 |
| 22 | FNTA | farnesyltransferase, CAAX box, alpha | 2339 |
| 23 | CRABP2 | cellular retinoic acid binding protein 2 | 1382 |
| 24 | DDX56 | DEAD-box helicase 56 | 54606 |
| 25 | WDR74 | WD repeat domain 74 | 54663 |
| 26 | FKBP8 | FKBP prolyl isomerase 8 | 23770 |
| 27 | SLC38A2 | solute carrier family 38 member 2 | 54407 |
| 28 | THBS1 | thrombospondin 1 | 7057 |
| 29 | CRKL | CRK like proto-oncogene, adaptor protein | 1399 |
| 30 | ELOB | elongin B | 6923 |
| 31 | PSMA5 | proteasome subunit alpha 5 | 5686 |
| 32 | PPP2R2A | protein phosphatase 2 regulatory subunit Balpha | 5520 |
| 33 | NSA2 | NSA2, ribosome biogenesis homolog | 10412 |
| 34 | FAT2 | FAT atypical cadherin 2 | 2196 |
| 35 | SAFB2 | scaffold attachment factor B2 | 9667 |
| 36 | DIAPH3 | diaphanous related formin 3 | 81624 |
| 37 | IL1A | interleukin 1 alpha | 3552 |
| 38 | LARP7 | La ribonucleoprotein domain family member 7 | 51574 |
| 39 | CRK | CRK proto-oncogene, adaptor protein | 1398 |
| 40 | NOP16 | NOP16 nucleolar protein | 51491 |
| 41 | YWHAQ | tyrosine 3-monooxygenase/tryptophan 5-monooxygenase activation protein theta | 10971 |
| 42 | RBM19 | RNA binding motif protein 19 | 9904 |
| 43 | CBWD2 | COBW domain containing 2 | 150472 |
| 44 | TRIM21 | tripartite motif containing 21 | 6737 |
| 45 | PKM | pyruvate kinase M1/2 | 5315 |
| 46 | PSMB9 | proteasome subunit beta 9 | 5698 |
| 47 | RFC4 | replication factor C subunit 4 | 5984 |
| 48 | BST2 | bone marrow stromal cell antigen 2 | 684 |
| 49 | S100A10 | S100 calcium binding protein A10 | 6281 |
| 50 | PLA2G4A | phospholipase A2 group IVA | 5321 |
| 51 | TARS2 | threonyl-tRNA synthetase 2, mitochondrial | 80222 |
| 52 | DPP7 | dipeptidyl peptidase 7 | 29952 |
| 53 | NIFK | nucleolar protein interacting with the FHA domain of MKI67 | 84365 |
| 54 | TNKS1BP1 | tankyrase 1 binding protein 1 | 85456 |
| 55 | RPS8 | ribosomal protein S8 | 6202 |
| 56 | ZG16B | zymogen granule protein 16B | 124220 |
| 57 | NOL7 | nucleolar protein 7 | 51406 |
| 58 | AKAP2 | A-kinase anchoring protein 2 | 11217 |
| 59 | DDX24 | DEAD-box helicase 24 | 57062 |
| 60 | OSTF1 | osteoclast stimulating factor 1 | 26578 |
| 61 | MDC1 | mediator of DNA damage checkpoint 1 | 9656 |
| 62 | NSFL1C | NSFL1 cofactor | 55968 |
| 63 | RFC1 | replication factor C subunit 1 | 5981 |
| 64 | BCLAF1 | BCL2 associated transcription factor 1 | 9774 |
| 65 | IDI1 | isopentenyl-diphosphate delta isomerase 1 | 3422 |
| 66 | LARP4 | La ribonucleoprotein domain family member 4 | 113251 |
| 67 | CDKN2AIP | CDKN2A interacting protein | 55602 |
| 68 | SERPINB8 | serpin family B member 8 | 5271 |
| 69 | PFDN2 | prefoldin subunit 2 | 5202 |
| 70 | SAFB | scaffold attachment factor B | 6294 |
| 71 | NOL10 | nucleolar protein 10 | 79954 |
| 72 | SCD | stearoyl-CoA desaturase | 6319 |
| 73 | SQSTM1 | sequestosome 1 | 8878 |
| 74 | NRBF2 | nuclear receptor binding factor 2 | 29982 |
| 75 | EIF6 | eukaryotic translation initiation factor 6 | 3692 |
| 76 | ITIH4 | inter-alpha-trypsin inhibitor heavy chain family member 4 | 3700 |
| 77 | DNTTIP2 | deoxynucleotidyltransferase terminal interacting protein 2 | 30836 |
| 78 | COTL1 | coactosin like F-actin binding protein 1 | 23406 |
| 79 | SMARCA5 | SWI/SNF related, matrix associated, actin dependent regulator of chromatin, subfamily a, member 5 | 8467 |
| 80 | MPZL1 | myelin protein zero like 1 | 9019 |
| 81 | DERPC | DERPC, proline and glycine rich nuclear protein | 1.13E+08 |
| 82 | RPL7L1 | ribosomal protein L7 like 1 | 285855 |
| 83 | HSPA9 | heat shock protein family A (Hsp70) member 9 | 3313 |
| 84 | LAMA5 | laminin subunit alpha 5 | 3911 |
| 85 | MRPS23 | mitochondrial ribosomal protein S23 | 51649 |
| 86 | TRMT1L | tRNA methyltransferase 1 like | 81627 |
| 87 | CMPK1 | cytidine/uridine monophosphate kinase 1 | 51727 |
| 88 | SH3GL1 | SH3 domain containing GRB2 like 1, endophilin A2 | 6455 |
| 89 | WDR77 | WD repeat domain 77 | 79084 |
| 90 | TRAPPC3 | trafficking protein particle complex 3 | 27095 |
| 91 | ZC3H11A | zinc finger CCCH-type containing 11A | 9877 |
| 92 | TP63 | tumor protein p63 | 8626 |
| 93 | CYFIP1 | cytoplasmic FMR1 interacting protein 1 | 23191 |
| 94 | VPS28 | VPS28, ESCRT-I subunit | 51160 |
| 95 | NFU1 | NFU1 iron-sulfur cluster scaffold | 27247 |
| 96 | C11orf68 | chromosome 11 open reading frame 68 | 83638 |
| 97 | CCDC6 | coiled-coil domain containing 6 | 8030 |
| 98 | BYSL | bystin like | 705 |
| 99 | ACTG1 | actin gamma 1 | 71 |
| 100 | ACTB | actin beta | 60 |
| 101 | ENOPH1 | enolase-phosphatase 1 | 58478 |
| 102 | GBF1 | golgi brefeldin A resistant guanine nucleotide exchange factor 1 | 8729 |
| 103 | GLOD4 | glyoxalase domain containing 4 | 51031 |
| 104 | ALDH18A1 | aldehyde dehydrogenase 18 family member A1 | 5832 |
| 105 | PPP1CC | protein phosphatase 1 catalytic subunit gamma | 5501 |
| 106 | EIPR1 | EARP complex and GARP complex interacting protein 1 | 7260 |
| 107 | HSBP1 | heat shock factor binding protein 1 | 3281 |
| 108 | CDH3 | cadherin 3 | 1001 |
| 109 | CSPG4 | chondroitin sulfate proteoglycan 4 | 1464 |
| 110 | GLG1 | golgi glycoprotein 1 | 2734 |
| 111 | PCMT1 | protein-L-isoaspartate (D-aspartate) O-methyltransferase | 5110 |
| 112 | TPX2 | TPX2, microtubule nucleation factor | 22974 |
| 113 | RNF114 | ring finger protein 114 | 55905 |
| 114 | PGM3 | phosphoglucomutase 3 | 5238 |
| 115 | BMS1 | BMS1, ribosome biogenesis factor | 9790 |
| 116 | SLC1A5 | solute carrier family 1 member 5 | 6510 |
| 117 | ZMYM2 | zinc finger MYM-type containing 2 | 7750 |
| 118 | RPL17 | ribosomal protein L17 | 6139 |
| 119 | SNRPA | small nuclear ribonucleoprotein polypeptide A | 6626 |
| 120 | NDUFAF2 | NADH:ubiquinone oxidoreductase complex assembly factor 2 | 91942 |
| 121 | PTK7 | protein tyrosine kinase 7 (inactive) | 5754 |
| 122 | S100A11 | S100 calcium binding protein A11 | 6282 |
| 123 | SMC4 | structural maintenance of chromosomes 4 | 10051 |
| 124 | TP53I3 | tumor protein p53 inducible protein 3 | 9540 |
| 125 | TUFT1 | tuftelin 1 | 7286 |
| 126 | RTF2 | replication termination factor 2 | 51507 |
| 127 | SMIM20 | small integral membrane protein 20 | 389203 |
| 128 | TIMM8B | translocase of inner mitochondrial membrane 8 homolog B | 26521 |
| 129 | ABHD14B | abhydrolase domain containing 14B | 84836 |
| 130 | TANC1 | tetratricopeptide repeat, ankyrin repeat and coiled-coil containing 1 | 85461 |
| 131 | PUM3 | pumilio RNA binding family member 3 | 9933 |
| 132 | CRYAB | crystallin alpha B | 1410 |
| 133 | CDSN | corneodesmosin | 1041 |
| 134 | MRPL58 | mitochondrial ribosomal protein L58 | 3396 |
| 135 | TMED7 | transmembrane p24 trafficking protein 7 | 51014 |
| 136 | IRF2BP2 | interferon regulatory factor 2 binding protein 2 | 359948 |
| 137 | MYDGF | myeloid derived growth factor | 56005 |
| 138 | KRT1 | keratin 1 | 3848 |
| 139 | KRT10 | keratin 10 | 3858 |
| 140 | RHOC | ras homolog family member C | 389 |
| 141 | NQO1 | NAD(P)H quinone dehydrogenase 1 | 1728 |
| 142 | BSG | basigin (Ok blood group) | 682 |
| 143 | MEPCE | methylphosphate capping enzyme | 56257 |
| 144 | CFL1 | cofilin 1 | 1072 |
| 145 | PRMT5 | protein arginine methyltransferase 5 | 10419 |
| 146 | GPC1 | glypican 1 | 2817 |
| 147 | OTUD4 | OTU deubiquitinase 4 | 54726 |
| 148 | PUF60 | poly(U) binding splicing factor 60 | 22827 |
| 149 | NFKB2 | nuclear factor kappa B subunit 2 | 4791 |
| 150 | POLD2 | DNA polymerase delta 2, accessory subunit | 5425 |
| 151 | ARPC4 | actin related protein 2/3 complex subunit 4 | 10093 |
| 152 | RRS1 | ribosome biogenesis regulator homolog | 23212 |
| 153 | TUBB2A | tubulin beta 2A class IIa | 7280 |
| 154 | PFN1 | profilin 1 | 5216 |
| 155 | NUSAP1 | nucleolar and spindle associated protein 1 | 51203 |
| 156 | MTA2 | metastasis associated 1 family member 2 | 9219 |
| 157 | SMARCE1 | SWI/SNF related, matrix associated, actin dependent regulator of chromatin, subfamily e, member 1 | 6605 |
| 158 | USO1 | USO1 vesicle transport factor | 8615 |
| 159 | SEC11A | SEC11 homolog A, signal peptidase complex subunit | 23478 |
| 160 | YWHAB | tyrosine 3-monooxygenase/tryptophan 5-monooxygenase activation protein beta | 7529 |
| 161 | SMC3 | structural maintenance of chromosomes 3 | 9126 |
| 162 | SARNP | SAP domain containing ribonucleoprotein | 84324 |
| 163 | ARHGDIB | Rho GDP dissociation inhibitor beta | 397 |
| 164 | SNRPF | small nuclear ribonucleoprotein polypeptide F | 6636 |
| 165 | OTUB1 | OTU deubiquitinase, ubiquitin aldehyde binding 1 | 55611 |
| 166 | GBE1 | 1,4-alpha-glucan branching enzyme 1 | 2632 |
| 167 | YTHDF2 | YTH N6-methyladenosine RNA binding protein 2 | 51441 |
| 168 | CUTA | cutA divalent cation tolerance homolog | 51596 |
| 169 | SSR4 | signal sequence receptor subunit 4 | 6748 |
| 170 | NQO2 | N-ribosyldihydronicotinamide:quinone reductase 2 | 4835 |
| 171 | PFDN5 | prefoldin subunit 5 | 5204 |
| 172 | DCTN3 | dynactin subunit 3 | 11258 |
| 173 | RBM8A | RNA binding motif protein 8A | 9939 |
| 174 | DHRS7 | dehydrogenase/reductase 7 | 51635 |
| 175 | CAV2 | caveolin 2 | 858 |
| 176 | PES1 | pescadillo ribosomal biogenesis factor 1 | 23481 |
| 177 | PRDX1 | peroxiredoxin 1 | 5052 |
| 178 | RBM26 | RNA binding motif protein 26 | 64062 |
| 179 | GRB2 | growth factor receptor bound protein 2 | 2885 |
| 180 | CPSF7 | cleavage and polyadenylation specific factor 7 | 79869 |
| 181 | GFPT1 | glutamine--fructose-6-phosphate transaminase 1 | 2673 |
| 182 | EFHD2 | EF-hand domain family member D2 | 79180 |
| 183 | CAPG | capping actin protein, gelsolin like | 822 |
| 184 | APP | amyloid beta precursor protein | 351 |
| 185 | RDH13 | retinol dehydrogenase 13 | 112724 |
| 186 | APOOL | apolipoprotein O like | 139322 |
| 187 | NUTF2 | nuclear transport factor 2 | 10204 |
| 188 | EXOSC4 | exosome component 4 | 54512 |
| 189 | BOLA2B | bolA family member 2B | 654483 |
| 190 | PEX13 | peroxisomal biogenesis factor 13 | 5194 |
| 191 | CAPZA1 | capping actin protein of muscle Z-line subunit alpha 1 | 829 |
| 192 | TXNDC12 | thioredoxin domain containing 12 | 51060 |
| 193 | CKS1B | CDC28 protein kinase regulatory subunit 1B | 1163 |
| 194 | PGRMC1 | progesterone receptor membrane component 1 | 10857 |
| 195 | RHOA | ras homolog family member A | 387 |
| 196 | NDRG1 | N-myc downstream regulated 1 | 10397 |
| 197 | AP1S1 | adaptor related protein complex 1 subunit sigma 1 | 1174 |
| 198 | GOT1 | glutamic-oxaloacetic transaminase 1 | 2805 |
| 199 | STRN4 | striatin 4 | 29888 |
| 200 | GCA | grancalcin | 25801 |
| 201 | TNIP1 | TNFAIP3 interacting protein 1 | 10318 |
| 202 | S100P | S100 calcium binding protein P | 6286 |
| 203 | RSL1D1 | ribosomal L1 domain containing 1 | 26156 |
| 204 | CPSF3 | cleavage and polyadenylation specific factor 3 | 51692 |
| 205 | CTNNBL1 | catenin beta like 1 | 56259 |
| 206 | ICAM1 | intercellular adhesion molecule 1 | 3383 |
| 207 | SOD1 | superoxide dismutase 1 | 6647 |
| 208 | STOM | stomatin | 2040 |
| 209 | CDC34 | cell division cycle 34 | 997 |
| 210 | ADD1 | adducin 1 | 118 |
| 211 | SPCS2 | signal peptidase complex subunit 2 | 9789 |
| 212 | YWHAZ | tyrosine 3-monooxygenase/tryptophan 5-monooxygenase activation protein zeta | 7534 |
| 213 | PHLDB1 | pleckstrin homology like domain family B member 1 | 23187 |
| 214 | SRXN1 | sulfiredoxin 1 | 140809 |
| 215 | VAC14 | Vac14, PIKFYVE complex component | 55697 |
| 216 | IL1B | interleukin 1 beta | 3553 |
| 217 | NCSTN | nicastrin | 23385 |
| 218 | RTKN | rhotekin | 6242 |
| 219 | PSEN1 | presenilin 1 | 5663 |
| 220 | RAD21 | RAD21 cohesin complex component | 5885 |
| 221 | MTA1 | metastasis associated 1 | 9112 |
| 222 | RRP1 | ribosomal RNA processing 1 | 8568 |
| 223 | IL1RN | interleukin 1 receptor antagonist | 3557 |
| 224 | RRP12 | ribosomal RNA processing 12 homolog | 23223 |
| 225 | YWHAG | tyrosine 3-monooxygenase/tryptophan 5-monooxygenase activation protein gamma | 7532 |
| 226 | PSME1 | proteasome activator subunit 1 | 5720 |
| 227 | FUBP1 | far upstream element binding protein 1 | 8880 |
| 228 | BAIAP2 | BAI1 associated protein 2 | 10458 |
| 229 | CCNB1 | cyclin B1 | 891 |
| 230 | TJP1 | tight junction protein 1 | 7082 |
| 231 | EPHX1 | epoxide hydrolase 1 | 2052 |
| 232 | EIF4H | eukaryotic translation initiation factor 4H | 7458 |
| 233 | NNMT | nicotinamide N-methyltransferase | 4837 |
| 234 | GPX1 | glutathione peroxidase 1 | 2876 |
| 235 | CFDP1 | craniofacial development protein 1 | 10428 |
| 236 | DNAJC7 | DnaJ heat shock protein family (Hsp40) member C7 | 7266 |
| 237 | PSMD10 | proteasome 26S subunit, non-ATPase 10 | 5716 |
| 238 | U2SURP | U2 snRNP associated SURP domain containing | 23350 |
| 239 | TPD52L2 | TPD52 like 2 | 7165 |
| 240 | FAM83B | family with sequence similarity 83 member B | 222584 |
| 241 | POLDIP3 | DNA polymerase delta interacting protein 3 | 84271 |
| 242 | EIF5A | eukaryotic translation initiation factor 5A | 1984 |
| 243 | AACS | acetoacetyl-CoA synthetase | 65985 |
| 244 | DDX54 | DEAD-box helicase 54 | 79039 |
| 245 | SLC44A1 | solute carrier family 44 member 1 | 23446 |
| 246 | RPL6 | ribosomal protein L6 | 6128 |
| 247 | PKP1 | plakophilin 1 | 5317 |
| 248 | TRMT5 | tRNA methyltransferase 5 | 57570 |
| 249 | RAP2B | RAP2B, member of RAS oncogene family | 5912 |
| 250 | TXNRD1 | thioredoxin reductase 1 | 7296 |
| 251 | UQCRC2 | ubiquinol-cytochrome c reductase core protein 2 | 7385 |
| 252 | KRT9 | keratin 9 | 3857 |
| 253 | CEBPZ | CCAAT enhancer binding protein zeta | 10153 |
| 254 | RPLP2 | ribosomal protein lateral stalk subunit P2 | 6181 |
| 255 | GAR1 | GAR1 ribonucleoprotein | 54433 |
| 256 | OCIAD2 | OCIA domain containing 2 | 132299 |
| 257 | MGME1 | mitochondrial genome maintenance exonuclease 1 | 92667 |
| 258 | GSTO1 | glutathione S-transferase omega 1 | 9446 |
| 259 | MRPL55 | mitochondrial ribosomal protein L55 | 128308 |
| 260 | P4HB | prolyl 4-hydroxylase subunit beta | 5034 |
| 261 | PITRM1 | pitrilysin metallopeptidase 1 | 10531 |
| 262 | PDS5A | PDS5 cohesin associated factor A | 23244 |
| 263 | ATP6V1A | ATPase H+ transporting V1 subunit A | 523 |
| 264 | SF1 | splicing factor 1 | 7536 |
| 265 | PFKFB2 | 6-phosphofructo-2-kinase/fructose-2,6-biphosphatase 2 | 5208 |
| 266 | PPP2R5D | protein phosphatase 2 regulatory subunit B'delta | 5528 |
| 267 | HSPA5 | heat shock protein family A (Hsp70) member 5 | 3309 |
| 268 | SART1 | spliceosome associated factor 1, recruiter of U4/U6.U5 tri-snRNP | 9092 |
| 269 | MRPL37 | mitochondrial ribosomal protein L37 | 51253 |
| 270 | DLG1 | discs large MAGUK scaffold protein 1 | 1739 |
| 271 | POFUT1 | protein O-fucosyltransferase 1 | 23509 |
| 272 | MRPL1 | mitochondrial ribosomal protein L1 | 65008 |
| 273 | COPS4 | COP9 signalosome subunit 4 | 51138 |
| 274 | THOC2 | THO complex 2 | 57187 |
| 275 | PCYT1A | phosphate cytidylyltransferase 1, choline, alpha | 5130 |
| 276 | TPBG | trophoblast glycoprotein | 7162 |
| 277 | FXR2 | FMR1 autosomal homolog 2 | 9513 |
| 278 | S100A9 | S100 calcium binding protein A9 | 6280 |
| 279 | FTSJ3 | FtsJ RNA methyltransferase homolog 3 | 117246 |
| 280 | RAB8A | RAB8A, member RAS oncogene family | 4218 |
| 281 | SPART | spartin | 23111 |
| 282 | LMNB2 | lamin B2 | 84823 |
| 283 | LMO7 | LIM domain 7 | 4008 |
| 284 | NOP2 | NOP2 nucleolar protein | 4839 |
| 285 | GSTK1 | glutathione S-transferase kappa 1 | 373156 |
| 286 | ANXA5 | annexin A5 | 308 |
| 287 | FAF1 | Fas associated factor 1 | 11124 |
| 288 | CELF1 | CUGBP Elav-like family member 1 | 10658 |
| 289 | AK1 | adenylate kinase 1 | 203 |
| 290 | SPCS3 | signal peptidase complex subunit 3 | 60559 |
| 291 | TSFM | Ts translation elongation factor, mitochondrial | 10102 |
| 292 | PSPH | phosphoserine phosphatase | 5723 |
| 293 | ANKRD17 | ankyrin repeat domain 17 | 26057 |
| 294 | GTF2I | general transcription factor IIi | 2969 |
| 295 | GNAI3 | G protein subunit alpha i3 | 2773 |
| 296 | CALML5 | calmodulin like 5 | 51806 |
| 297 | KIAA1671 | KIAA1671 | 85379 |
| 298 | DAG1 | dystroglycan 1 | 1605 |
| 299 | C12orf29 | chromosome 12 open reading frame 29 | 91298 |
| 300 | MKI67 | marker of proliferation Ki-67 | 4288 |
| 301 | CYP2S1 | cytochrome P450 family 2 subfamily S member 1 | 29785 |
| 302 | NANS | N-acetylneuraminate synthase | 54187 |
| 303 | RPL12 | ribosomal protein L12 | 6136 |
| 304 | UCHL1 | ubiquitin C-terminal hydrolase L1 | 7345 |
| 305 | NT5DC1 | 5'-nucleotidase domain containing 1 | 221294 |
| 306 | PHF6 | PHD finger protein 6 | 84295 |
| 307 | FLYWCH2 | FLYWCH family member 2 | 114984 |
| 308 | LSM6 | LSM6 homolog, U6 small nuclear RNA and mRNA degradation associated | 11157 |
| 309 | EXOSC6 | exosome component 6 | 118460 |
| 310 | RPA2 | replication protein A2 | 6118 |
| 311 | DDX10 | DEAD-box helicase 10 | 1662 |
| 312 | AHCTF1 | AT-hook containing transcription factor 1 | 25909 |
| 313 | PEX14 | peroxisomal biogenesis factor 14 | 5195 |
| 314 | HMGN1 | high mobility group nucleosome binding domain 1 | 3150 |
| 315 | G3BP1 | G3BP stress granule assembly factor 1 | 10146 |
| 316 | GALNT6 | polypeptide N-acetylgalactosaminyltransferase 6 | 11226 |
| 317 | BRIX1 | BRX1, biogenesis of ribosomes | 55299 |
| 318 | HSPB1 | heat shock protein family B (small) member 1 | 3315 |
| 319 | PARK7 | Parkinsonism associated deglycase | 11315 |
| 320 | APEH | acylaminoacyl-peptide hydrolase | 327 |
| 321 | ANXA3 | annexin A3 | 306 |
| 322 | PPM1G | protein phosphatase, Mg2+/Mn2+ dependent 1G | 5496 |
| 323 | MMAB | metabolism of cobalamin associated B | 326625 |
| 324 | CDC20 | cell division cycle 20 | 991 |
| 325 | OSBPL8 | oxysterol binding protein like 8 | 114882 |
| 326 | NNT | nicotinamide nucleotide transhydrogenase | 23530 |
| 327 | CSNK1A1 | casein kinase 1 alpha 1 | 1452 |
| 328 | TCERG1 | transcription elongation regulator 1 | 10915 |
| 329 | NUDT15 | nudix hydrolase 15 | 55270 |
| 330 | ARL6IP5 | ADP ribosylation factor like GTPase 6 interacting protein 5 | 10550 |
| 331 | GOLPH3 | golgi phosphoprotein 3 | 64083 |
| 332 | SCARB2 | scavenger receptor class B member 2 | 950 |
| 333 | VIM | vimentin | 7431 |
| 334 | DDX17 | DEAD-box helicase 17 | 10521 |
| 335 | DNASE2 | deoxyribonuclease 2, lysosomal | 1777 |
| 336 | SSR1 | signal sequence receptor subunit 1 | 6745 |
| 337 | TAP2 | transporter 2, ATP binding cassette subfamily B member | 6891 |
| 338 | VASP | vasodilator stimulated phosphoprotein | 7408 |
| 339 | COPG1 | coatomer protein complex subunit gamma 1 | 22820 |
| 340 | GSS | glutathione synthetase | 2937 |
| 341 | PGAM1 | phosphoglycerate mutase 1 | 5223 |
| 342 | C5orf22 | chromosome 5 open reading frame 22 | 55322 |
| 343 | CD58 | CD58 molecule | 965 |
| 344 | RTN3 | reticulon 3 | 10313 |
| 345 | ETFA | electron transfer flavoprotein subunit alpha | 2108 |
| 346 | COPE | coatomer protein complex subunit epsilon | 11316 |
| 347 | ASNA1 | arsA arsenite transporter, ATP-binding, homolog 1 (bacterial) | 439 |
| 348 | SMARCC1 | SWI/SNF related, matrix associated, actin dependent regulator of chromatin subfamily c member 1 | 6599 |
| 349 | NUDT1 | nudix hydrolase 1 | 4521 |
| 350 | ACAA2 | acetyl-CoA acyltransferase 2 | 10449 |
| 351 | VRK1 | VRK serine/threonine kinase 1 | 7443 |
| 352 | DDX60 | DExD/H-box helicase 60 | 55601 |
| 353 | RAP1GDS1 | Rap1 GTPase-GDP dissociation stimulator 1 | 5910 |
| 354 | SLC30A1 | solute carrier family 30 member 1 | 7779 |
| 355 | MORF4L1 | mortality factor 4 like 1 | 10933 |
| 356 | SERPINB5 | serpin family B member 5 | 5268 |
| 357 | LRRC40 | leucine rich repeat containing 40 | 55631 |
| 358 | COX7A2L | cytochrome c oxidase subunit 7A2 like | 9167 |
| 359 | HTRA1 | HtrA serine peptidase 1 | 5654 |
| 360 | COL17A1 | collagen type XVII alpha 1 chain | 1308 |
| 361 | TAGLN | transgelin | 6876 |
| 362 | TRIR | telomerase RNA component interacting RNase | 79002 |
| 363 | TMEM87A | transmembrane protein 87A | 25963 |
| 364 | TSTD1 | thiosulfate sulfurtransferase like domain containing 1 | 1E+08 |
| 365 | RTN4IP1 | reticulon 4 interacting protein 1 | 84816 |
| 366 | CHMP5 | charged multivesicular body protein 5 | 51510 |
| 367 | DBT | dihydrolipoamide branched chain transacylase E2 | 1629 |
| 368 | FGFBP1 | fibroblast growth factor binding protein 1 | 9982 |
| 369 | GLRX3 | glutaredoxin 3 | 10539 |
| 370 | ATG5 | autophagy related 5 | 9474 |
| 371 | ADA | adenosine deaminase | 100 |
| 372 | NAXE | NAD(P)HX epimerase | 128240 |
| 373 | WRNIP1 | Werner helicase interacting protein 1 | 56897 |
| 374 | ANKLE2 | ankyrin repeat and LEM domain containing 2 | 23141 |
| 375 | TUBA4A | tubulin alpha 4a | 7277 |
| 376 | MRPS14 | mitochondrial ribosomal protein S14 | 63931 |
| 377 | LCP1 | lymphocyte cytosolic protein 1 | 3936 |
| 378 | SNRNP200 | small nuclear ribonucleoprotein U5 subunit 200 | 23020 |
| 379 | LAMP1 | lysosomal associated membrane protein 1 | 3916 |
| 380 | NUP107 | nucleoporin 107 | 57122 |
| 381 | GCLC | glutamate-cysteine ligase catalytic subunit | 2729 |
| 382 | ITPA | inosine triphosphatase | 3704 |
| 383 | DCUN1D1 | defective in cullin neddylation 1 domain containing 1 | 54165 |
| 384 | NCLN | nicalin | 56926 |
| 385 | PRDX5 | peroxiredoxin 5 | 25824 |
| 386 | PTRHD1 | peptidyl-tRNA hydrolase domain containing 1 | 391356 |
| 387 | BAG6 | BCL2 associated athanogene 6 | 7917 |
| 388 | MYO1B | myosin IB | 4430 |
| 389 | PPA1 | pyrophosphatase (inorganic) 1 | 5464 |
| 390 | ANXA7 | annexin A7 | 310 |
| 391 | ITPRID2 | ITPR interacting domain containing 2 | 6744 |
| 392 | G6PD | glucose-6-phosphate dehydrogenase | 2539 |
| 393 | DHX36 | DEAH-box helicase 36 | 170506 |
| 394 | PA2G4 | proliferation-associated 2G4 | 5036 |
| 395 | CDK4 | cyclin dependent kinase 4 | 1019 |
| 396 | RRAGA | Ras related GTP binding A | 10670 |
| 397 | DPP3 | dipeptidyl peptidase 3 | 10072 |
| 398 | RBM15 | RNA binding motif protein 15 | 64783 |
| 399 | GTPBP4 | GTP binding protein 4 | 23560 |
| 400 | EEF1G | eukaryotic translation elongation factor 1 gamma | 1937 |
| 401 | EDF1 | endothelial differentiation related factor 1 | 8721 |
| 402 | DBNL | drebrin like | 28988 |
| 403 | DSG1 | desmoglein 1 | 1828 |
| 404 | OR1M1 | olfactory receptor family 1 subfamily M member 1 | 125963 |
| 405 | RRP7A | ribosomal RNA processing 7 homolog A | 27341 |
| 406 | ANXA6 | annexin A6 | 309 |
| 407 | PIGR | polymeric immunoglobulin receptor | 5284 |
| 408 | PDIA6 | protein disulfide isomerase family A member 6 | 10130 |
| 409 | RAB7A | RAB7A, member RAS oncogene family | 7879 |
| 410 | CHP1 | calcineurin like EF-hand protein 1 | 11261 |
| 411 | DIMT1 | DIM1 dimethyladenosine transferase 1 homolog | 27292 |
| 412 | TMED1 | transmembrane p24 trafficking protein 1 | 11018 |
| 413 | PMM2 | phosphomannomutase 2 | 5373 |
| 414 | ARHGAP1 | Rho GTPase activating protein 1 | 392 |
| 415 | CSTF1 | cleavage stimulation factor subunit 1 | 1477 |
| 416 | NOL6 | nucleolar protein 6 | 65083 |
| 417 | WDR61 | WD repeat domain 61 | 80349 |
| 418 | TCP1 | t-complex 1 | 6950 |
| 419 | FAR1 | fatty acyl-CoA reductase 1 | 84188 |
| 420 | ATP5ME | ATP synthase membrane subunit e | 521 |
| 421 | PFN2 | profilin 2 | 5217 |
| 422 | CCT8 | chaperonin containing TCP1 subunit 8 | 10694 |
| 423 | CCDC47 | coiled-coil domain containing 47 | 57003 |
| 424 | LSM2 | LSM2 homolog, U6 small nuclear RNA and mRNA degradation associated | 57819 |
| 425 | RPL19 | ribosomal protein L19 | 6143 |
| 426 | PISD | phosphatidylserine decarboxylase | 23761 |
| 427 | NAT10 | N-acetyltransferase 10 | 55226 |
| 428 | RBM4 | RNA binding motif protein 4 | 5936 |
| 429 | MRPS22 | mitochondrial ribosomal protein S22 | 56945 |
| 430 | PSMC6 | proteasome 26S subunit, ATPase 6 | 5706 |
| 431 | TSTA3 | tissue specific transplantation antigen P35B | 7264 |
| 432 | RAB32 | RAB32, member RAS oncogene family | 10981 |
| 433 | ATP1A1 | ATPase Na+/K+ transporting subunit alpha 1 | 476 |
| 434 | IGFBP7 | insulin like growth factor binding protein 7 | 3490 |
| 435 | CASP4 | caspase 4 | 837 |
| 436 | SND1 | staphylococcal nuclease and tudor domain containing 1 | 27044 |
| 437 | ANXA2 | annexin A2 | 302 |
| 438 | PPP1R12A | protein phosphatase 1 regulatory subunit 12A | 4659 |
| 439 | DHFR | dihydrofolate reductase | 1719 |
| 440 | DARS2 | aspartyl-tRNA synthetase 2, mitochondrial | 55157 |
| 441 | TPD52 | tumor protein D52 | 7163 |
| 442 | SNRPB2 | small nuclear ribonucleoprotein polypeptide B2 | 6629 |
| 443 | PYGL | glycogen phosphorylase L | 5836 |
| 444 | PGD | phosphogluconate dehydrogenase | 5226 |
| 445 | ECHDC1 | ethylmalonyl-CoA decarboxylase 1 | 55862 |
| 446 | ITGA2 | integrin subunit alpha 2 | 3673 |
| 447 | UGP2 | UDP-glucose pyrophosphorylase 2 | 7360 |
| 448 | PHGDH | phosphoglycerate dehydrogenase | 26227 |
| 449 | DDX23 | DEAD-box helicase 23 | 9416 |
| 450 | PPAN-P2RY11 | PPAN-P2RY11 readthrough | 692312 |
| 451 | PTGES | prostaglandin E synthase | 9536 |
| 452 | LGALS7 | galectin 7 | 3963 |
| 453 | TSG101 | tumor susceptibility 101 | 7251 |
| 454 | SERPINB2 | serpin family B member 2 | 5055 |
| 455 | AP2A1 | adaptor related protein complex 2 subunit alpha 1 | 160 |
| 456 | HLA-A | major histocompatibility complex, class I, A | 3105 |
| 457 | FAM98B | family with sequence similarity 98 member B | 283742 |
| 458 | RPL32 | ribosomal protein L32 | 6161 |
| 459 | SNX3 | sorting nexin 3 | 8724 |
| 460 | FTH1 | ferritin heavy chain 1 | 2495 |
| 461 | GPNMB | glycoprotein nmb | 10457 |
| 462 | PTER | phosphotriesterase related | 9317 |
| 463 | IFI35 | interferon induced protein 35 | 3430 |
| 464 | PCK2 | phosphoenolpyruvate carboxykinase 2, mitochondrial | 5106 |
| 465 | YIPF3 | Yip1 domain family member 3 | 25844 |
| 466 | EIF3F | eukaryotic translation initiation factor 3 subunit F | 8665 |
| 467 | FTO | FTO, alpha-ketoglutarate dependent dioxygenase | 79068 |
| 468 | RAP1B | RAP1B, member of RAS oncogene family | 5908 |
| 469 | RACGAP1 | Rac GTPase activating protein 1 | 29127 |
| 470 | ACSL3 | acyl-CoA synthetase long chain family member 3 | 2181 |
| 471 | RPS11 | ribosomal protein S11 | 6205 |
| 472 | PRPF8 | pre-mRNA processing factor 8 | 10594 |
| 473 | APRT | adenine phosphoribosyltransferase | 353 |
| 474 | GSTP1 | glutathione S-transferase pi 1 | 2950 |
| 475 | CDCP1 | CUB domain containing protein 1 | 64866 |
| 476 | PON2 | paraoxonase 2 | 5445 |
| 477 | KRT2 | keratin 2 | 3849 |
| 478 | SNRPD2 | small nuclear ribonucleoprotein D2 polypeptide | 6633 |
| 479 | REEP5 | receptor accessory protein 5 | 7905 |
| 480 | OPA1 | OPA1, mitochondrial dynamin like GTPase | 4976 |
| 481 | CKAP4 | cytoskeleton associated protein 4 | 10970 |
| 482 | SH3GLB1 | SH3 domain containing GRB2 like, endophilin B1 | 51100 |
| 483 | DLAT | dihydrolipoamide S-acetyltransferase | 1737 |
| 484 | PDIA4 | protein disulfide isomerase family A member 4 | 9601 |
| 485 | PPP6R1 | protein phosphatase 6 regulatory subunit 1 | 22870 |
| 486 | CDK12 | cyclin dependent kinase 12 | 51755 |
| 487 | PLIN3 | perilipin 3 | 10226 |
| 488 | ITGA5 | integrin subunit alpha 5 | 3678 |
| 489 | SPTLC1 | serine palmitoyltransferase long chain base subunit 1 | 10558 |
| 490 | STRAP | serine/threonine kinase receptor associated protein | 11171 |
| 491 | ZW10 | zw10 kinetochore protein | 9183 |
| 492 | MCM5 | minichromosome maintenance complex component 5 | 4174 |
| 493 | TRIP12 | thyroid hormone receptor interactor 12 | 9320 |
| 494 | TGM1 | transglutaminase 1 | 7051 |
| 495 | SRSF5 | serine and arginine rich splicing factor 5 | 6430 |
| 496 | TOP2A | DNA topoisomerase II alpha | 7153 |
| 497 | LSR | lipolysis stimulated lipoprotein receptor | 51599 |
| 498 | EIF4G2 | eukaryotic translation initiation factor 4 gamma 2 | 1982 |
| 499 | MYO1E | myosin IE | 4643 |
| 500 | ETHE1 | ETHE1, persulfide dioxygenase | 23474 |
| 501 | GALK1 | galactokinase 1 | 2584 |
| 502 | CANX | calnexin | 821 |
| 503 | HNRNPA1P48 | heterogeneous nuclear ribonucleoprotein A1 pseudogene 48 | 642659 |
| 504 | RSF1 | remodeling and spacing factor 1 | 51773 |
| 505 | TRA2B | transformer 2 beta homolog | 6434 |
| 506 | CAPNS1 | calpain small subunit 1 | 826 |
| 507 | RAB9A | RAB9A, member RAS oncogene family | 9367 |
| 508 | CCT3 | chaperonin containing TCP1 subunit 3 | 7203 |
| 509 | DHX9 | DExH-box helicase 9 | 1660 |
| 510 | DNAJB12 | DnaJ heat shock protein family (Hsp40) member B12 | 54788 |
| 511 | SNRPD3 | small nuclear ribonucleoprotein D3 polypeptide | 6634 |
| 512 | MTCH2 | mitochondrial carrier 2 | 23788 |
| 513 | UBE2N | ubiquitin conjugating enzyme E2 N | 7334 |
| 514 | GATAD2B | GATA zinc finger domain containing 2B | 57459 |
| 515 | DNAJA3 | DnaJ heat shock protein family (Hsp40) member A3 | 9093 |
| 516 | RPS3 | ribosomal protein S3 | 6188 |
| 517 | LZIC | leucine zipper and CTNNBIP1 domain containing | 84328 |
| 518 | LIG1 | DNA ligase 1 | 3978 |
| 519 | LYN | LYN proto-oncogene, Src family tyrosine kinase | 4067 |
| 520 | GLO1 | glyoxalase I | 2739 |
| 521 | MIF | macrophage migration inhibitory factor | 4282 |
| 522 | PAF1 | PAF1 homolog, Paf1/RNA polymerase II complex component | 54623 |
| 523 | RAB29 | RAB29, member RAS oncogene family | 8934 |
| 524 | ITPR3 | inositol 1,4,5-trisphosphate receptor type 3 | 3710 |
| 525 | CD82 | CD82 molecule | 3732 |
| 526 | TNS4 | tensin 4 | 84951 |
| 527 | RABEP1 | rabaptin, RAB GTPase binding effector protein 1 | 9135 |
| 528 | CAPRIN1 | cell cycle associated protein 1 | 4076 |
| 529 | UBQLN1 | ubiquilin 1 | 29979 |
| 530 | RBP1 | retinol binding protein 1 | 5947 |
| 531 | HSPE1 | heat shock protein family E (Hsp10) member 1 | 3336 |
| 532 | SEC63 | SEC63 homolog, protein translocation regulator | 11231 |
| 533 | LYPLA2 | lysophospholipase 2 | 11313 |
| 534 | SNF8 | SNF8, ESCRT-II complex subunit | 11267 |
| 535 | CSTF2 | cleavage stimulation factor subunit 2 | 1478 |
| 536 | CLPTM1L | CLPTM1 like | 81037 |
| 537 | DDI2 | DNA damage inducible 1 homolog 2 | 84301 |
| 538 | LRRC47 | leucine rich repeat containing 47 | 57470 |
| 539 | EPS8L2 | EPS8 like 2 | 64787 |
| 540 | BICD2 | BICD cargo adaptor 2 | 23299 |
| 541 | EIF1AD | eukaryotic translation initiation factor 1A domain containing | 84285 |
| 542 | AAMP | angio associated migratory cell protein | 14 |
| 543 | ASS1 | argininosuccinate synthase 1 | 445 |
| 544 | AKR1A1 | aldo-keto reductase family 1 member A1 | 10327 |
| 545 | UBA6 | ubiquitin like modifier activating enzyme 6 | 55236 |
| 546 | PSAT1 | phosphoserine aminotransferase 1 | 29968 |
| 547 | PSMB6 | proteasome subunit beta 6 | 5694 |
| 548 | TBL3 | transducin beta like 3 | 10607 |
| 549 | SNW1 | SNW domain containing 1 | 22938 |
| 550 | IRGQ | immunity related GTPase Q | 126298 |
| 551 | JUN | Jun proto-oncogene, AP-1 transcription factor subunit | 3725 |
| 552 | PRRC2C | proline rich coiled-coil 2C | 23215 |
| 553 | LAMB1 | laminin subunit beta 1 | 3912 |
| 554 | XRN2 | 5'-3' exoribonuclease 2 | 22803 |
| 555 | CTBP2 | C-terminal binding protein 2 | 1488 |
| 556 | IDH1 | isocitrate dehydrogenase (NADP(+)) 1, cytosolic | 3417 |
| 557 | NDE1 | nudE neurodevelopment protein 1 | 54820 |
| 558 | GLDC | glycine decarboxylase | 2731 |
| 559 | LAMTOR2 | late endosomal/lysosomal adaptor, MAPK and MTOR activator 2 | 28956 |
| 560 | VPS4B | vacuolar protein sorting 4 homolog B | 9525 |
| 561 | PRPS2 | phosphoribosyl pyrophosphate synthetase 2 | 5634 |
| 562 | THRAP3 | thyroid hormone receptor associated protein 3 | 9967 |
| 563 | SCAMP1 | secretory carrier membrane protein 1 | 9522 |
| 564 | TAPBP | TAP binding protein | 6892 |
| 565 | RNMT | RNA guanine-7 methyltransferase | 8731 |
| 566 | CHORDC1 | cysteine and histidine rich domain containing 1 | 26973 |
| 567 | TYMS | thymidylate synthetase | 7298 |
| 568 | DDT | D-dopachrome tautomerase | 1652 |
| 569 | ACTC1 | actin alpha cardiac muscle 1 | 70 |
| 570 | HEATR1 | HEAT repeat containing 1 | 55127 |
| 571 | ERGIC1 | endoplasmic reticulum-golgi intermediate compartment 1 | 57222 |
| 572 | PGM2 | phosphoglucomutase 2 | 55276 |
| 573 | PPP4C | protein phosphatase 4 catalytic subunit | 5531 |
| 574 | AKR7A2 | aldo-keto reductase family 7 member A2 | 8574 |
| 575 | COPS2 | COP9 signalosome subunit 2 | 9318 |
| 576 | HNRNPU | heterogeneous nuclear ribonucleoprotein U | 3192 |
| 577 | OXSR1 | oxidative stress responsive kinase 1 | 9943 |
| 578 | REXO2 | RNA exonuclease 2 | 25996 |
| 579 | STIP1 | stress induced phosphoprotein 1 | 10963 |
| 580 | RIOK1 | RIO kinase 1 | 83732 |
| 581 | GAPVD1 | GTPase activating protein and VPS9 domains 1 | 26130 |
| 582 | RECQL | RecQ like helicase | 5965 |
| 583 | M6PR | mannose-6-phosphate receptor, cation dependent | 4074 |
| 584 | ECHDC3 | enoyl-CoA hydratase domain containing 3 | 79746 |
| 585 | ARL3 | ADP ribosylation factor like GTPase 3 | 403 |
| 586 | DDX21 | DExD-box helicase 21 | 9188 |
| 587 | GSN | gelsolin | 2934 |
| 588 | CASP14 | caspase 14 | 23581 |
| 589 | ZCCHC8 | zinc finger CCHC-type containing 8 | 55596 |
| 590 | RPS27 | ribosomal protein S27 | 6232 |
| 591 | NUBP1 | nucleotide binding protein 1 | 4682 |
| 592 | AHCYL1 | adenosylhomocysteinase like 1 | 10768 |
| 593 | SRSF7 | serine and arginine rich splicing factor 7 | 6432 |
| 594 | SEC14L2 | SEC14 like lipid binding 2 | 23541 |
| 595 | CHMP4B | charged multivesicular body protein 4B | 128866 |
| 596 | GNL1 | G protein nucleolar 1 (putative) | 2794 |
| 597 | SPECC1 | sperm antigen with calponin homology and coiled-coil domains 1 | 92521 |
| 598 | TOMM5 | translocase of outer mitochondrial membrane 5 | 401505 |
| 599 | WDR55 | WD repeat domain 55 | 54853 |
| 600 | ECPAS | Ecm29 proteasome adaptor and scaffold | 23392 |
| 601 | MRPL4 | mitochondrial ribosomal protein L4 | 51073 |
| 602 | KPNA1 | karyopherin subunit alpha 1 | 3836 |
| 603 | EHD1 | EH domain containing 1 | 10938 |
| 604 | ASPH | aspartate beta-hydroxylase | 444 |
| 605 | SMCHD1 | structural maintenance of chromosomes flexible hinge domain containing 1 | 23347 |
| 606 | API5 | apoptosis inhibitor 5 | 8539 |
| 607 | ROCK2 | Rho associated coiled-coil containing protein kinase 2 | 9475 |
| 608 | PRDX4 | peroxiredoxin 4 | 10549 |
| 609 | CYGB | cytoglobin | 114757 |
| 610 | SMTN | smoothelin | 6525 |
| 611 | PPHLN1 | periphilin 1 | 51535 |
| 612 | ELP1 | elongator complex protein 1 | 8518 |
| 613 | ATP6AP2 | ATPase H+ transporting accessory protein 2 | 10159 |
| 614 | ATG3 | autophagy related 3 | 64422 |
| 615 | CDC123 | cell division cycle 123 | 8872 |
| 616 | FOXK1 | forkhead box K1 | 221937 |
| 617 | GMFB | glia maturation factor beta | 2764 |
| 618 | SEMG2 | semenogelin 2 | 6407 |
| 619 | EPB41L2 | erythrocyte membrane protein band 4.1 like 2 | 2037 |
| 620 | SAE1 | SUMO1 activating enzyme subunit 1 | 10055 |
| 621 | P4HA1 | prolyl 4-hydroxylase subunit alpha 1 | 5033 |
| 622 | SRI | sorcin | 6717 |
| 623 | DSG3 | desmoglein 3 | 1830 |
| 624 | SEPHS1 | selenophosphate synthetase 1 | 22929 |
| 625 | GMPPA | GDP-mannose pyrophosphorylase A | 29926 |
| 626 | DLD | dihydrolipoamide dehydrogenase | 1738 |
| 627 | TFAM | transcription factor A, mitochondrial | 7019 |
| 628 | PTGFRN | prostaglandin F2 receptor inhibitor | 5738 |
| 629 | TM9SF2 | transmembrane 9 superfamily member 2 | 9375 |
| 630 | BCR | BCR, RhoGEF and GTPase activating protein | 613 |
| 631 | TMEM189-UBE2V1 | TMEM189-UBE2V1 readthrough | 387522 |
| 632 | DAPK3 | death associated protein kinase 3 | 1613 |
| 633 | PRPF19 | pre-mRNA processing factor 19 | 27339 |
| 634 | MAP1LC3B | microtubule associated protein 1 light chain 3 beta | 81631 |
| 635 | USP14 | ubiquitin specific peptidase 14 | 9097 |
| 636 | UBE2C | ubiquitin conjugating enzyme E2 C | 11065 |
| 637 | SYAP1 | synapse associated protein 1 | 94056 |
| 638 | GNS | glucosamine (N-acetyl)-6-sulfatase | 2799 |
| 639 | NEDD8-MDP1 | NEDD8-MDP1 readthrough | 1.01E+08 |
| 640 | TXNDC5 | thioredoxin domain containing 5 | 81567 |
| 641 | PCNP | PEST proteolytic signal containing nuclear protein | 57092 |
| 642 | TCEA1 | transcription elongation factor A1 | 6917 |
| 643 | ESF1 | ESF1 nucleolar pre-rRNA processing protein homolog | 51575 |
| 644 | IWS1 | IWS1, SUPT6H interacting protein | 55677 |
| 645 | HSD17B4 | hydroxysteroid 17-beta dehydrogenase 4 | 3295 |
| 646 | GMPPB | GDP-mannose pyrophosphorylase B | 29925 |
| 647 | VBP1 | VHL binding protein 1 | 7411 |
| 648 | ADAR | adenosine deaminase, RNA specific | 103 |
| 649 | CBSL | cystathionine-beta-synthase like | 1.03E+08 |
| 650 | NDUFA2 | NADH:ubiquinone oxidoreductase subunit A2 | 4695 |
| 651 | N4BP1 | NEDD4 binding protein 1 | 9683 |
| 652 | CLIC4 | chloride intracellular channel 4 | 25932 |
| 653 | NUCKS1 | nuclear casein kinase and cyclin dependent kinase substrate 1 | 64710 |
| 654 | LUC7L3 | LUC7 like 3 pre-mRNA splicing factor | 51747 |
| 655 | NAPRT | nicotinate phosphoribosyltransferase | 93100 |
| 656 | CSNK1D | casein kinase 1 delta | 1453 |
| 657 | CFL2 | cofilin 2 | 1073 |
| 658 | PRDX3 | peroxiredoxin 3 | 10935 |
| 659 | STMN1 | stathmin 1 | 3925 |
| 660 | ACO1 | aconitase 1 | 48 |
| 661 | CHERP | calcium homeostasis endoplasmic reticulum protein | 10523 |
| 662 | ACP2 | acid phosphatase 2, lysosomal | 53 |
| 663 | GPD2 | glycerol-3-phosphate dehydrogenase 2 | 2820 |
| 664 | PTMA | prothymosin alpha | 5757 |
| 665 | PACSIN3 | protein kinase C and casein kinase substrate in neurons 3 | 29763 |
| 666 | HNRNPR | heterogeneous nuclear ribonucleoprotein R | 10236 |
| 667 | ARPC5L | actin related protein 2/3 complex subunit 5 like | 81873 |
| 668 | CASP1 | caspase 1 | 834 |
| 669 | PRPF4B | pre-mRNA processing factor 4B | 8899 |
| 670 | NDFIP2 | Nedd4 family interacting protein 2 | 54602 |
| 671 | ATP2C1 | ATPase secretory pathway Ca2+ transporting 1 | 27032 |
| 672 | PPAT | phosphoribosyl pyrophosphate amidotransferase | 5471 |
| 673 | MTHFD1 | methylenetetrahydrofolate dehydrogenase, cyclohydrolase and formyltetrahydrofolate synthetase 1 | 4522 |
| 674 | MARCKSL1 | MARCKS like 1 | 65108 |
| 675 | GATAD2A | GATA zinc finger domain containing 2A | 54815 |
| 676 | TFRC | transferrin receptor | 7037 |
| 677 | SEMG1 | semenogelin 1 | 6406 |
| 678 | HEBP1 | heme binding protein 1 | 50865 |
| 679 | UBE2Z | ubiquitin conjugating enzyme E2 Z | 65264 |
| 680 | PRKACB | protein kinase cAMP-activated catalytic subunit beta | 5567 |
| 681 | ZNF622 | zinc finger protein 622 | 90441 |
| 682 | RBM12 | RNA binding motif protein 12 | 10137 |
| 683 | RNH1 | ribonuclease/angiogenin inhibitor 1 | 6050 |
| 684 | ANP32B | acidic nuclear phosphoprotein 32 family member B | 10541 |
| 685 | ANXA11 | annexin A11 | 311 |
| 686 | NUP54 | nucleoporin 54 | 53371 |
| 687 | DST | dystonin | 667 |
| 688 | CD276 | CD276 molecule | 80381 |
| 689 | CSTF2T | cleavage stimulation factor subunit 2 tau variant | 23283 |
| 690 | TM9SF4 | transmembrane 9 superfamily member 4 | 9777 |
| 691 | PAIP1 | poly(A) binding protein interacting protein 1 | 10605 |
| 692 | TRA2A | transformer 2 alpha homolog | 29896 |
| 693 | DNAJC2 | DnaJ heat shock protein family (Hsp40) member C2 | 27000 |
| 694 | PNP | purine nucleoside phosphorylase | 4860 |
| 695 | ATP5MD | ATP synthase membrane subunit DAPIT | 84833 |
| 696 | FLG2 | filaggrin family member 2 | 388698 |
| 697 | SNX5 | sorting nexin 5 | 27131 |
| 698 | SUGT1 | SGT1 homolog, MIS12 kinetochore complex assembly cochaperone | 10910 |
| 699 | RETREG3 | reticulophagy regulator family member 3 | 162427 |
| 700 | STK3 | serine/threonine kinase 3 | 6788 |
| 701 | SRM | spermidine synthase | 6723 |
| 702 | PRPF6 | pre-mRNA processing factor 6 | 24148 |
| 703 | CHTOP | chromatin target of PRMT1 | 26097 |
| 704 | HEXA | hexosaminidase subunit alpha | 3073 |
| 705 | TACO1 | translational activator of cytochrome c oxidase I | 51204 |
| 706 | HNRNPH3 | heterogeneous nuclear ribonucleoprotein H3 | 3189 |
| 707 | HSP90B1 | heat shock protein 90 beta family member 1 | 7184 |
| 708 | TMEM106B | transmembrane protein 106B | 54664 |
| 709 | LAD1 | ladinin 1 | 3898 |
| 710 | PYCR3 | pyrroline-5-carboxylate reductase 3 | 65263 |
| 711 | NDUFV2 | NADH:ubiquinone oxidoreductase core subunit V2 | 4729 |
| 712 | ADH5 | alcohol dehydrogenase 5 (class III), chi polypeptide | 128 |
| 713 | EMC7 | ER membrane protein complex subunit 7 | 56851 |
| 714 | DDX39A | DExD-box helicase 39A | 10212 |
| 715 | CIAO2B | cytosolic iron-sulfur assembly component 2B | 51647 |
| 716 | TPP2 | tripeptidyl peptidase 2 | 7174 |
| 717 | PI4K2A | phosphatidylinositol 4-kinase type 2 alpha | 55361 |
| 718 | TXN | thioredoxin | 7295 |
| 719 | NAA30 | N(alpha)-acetyltransferase 30, NatC catalytic subunit | 122830 |
| 720 | HSPA4 | heat shock protein family A (Hsp70) member 4 | 3308 |
| 721 | PDCD5 | programmed cell death 5 | 9141 |
| 722 | SNX12 | sorting nexin 12 | 29934 |
| 723 | DBI | diazepam binding inhibitor, acyl-CoA binding protein | 1622 |
| 724 | UGGT1 | UDP-glucose glycoprotein glucosyltransferase 1 | 56886 |
| 725 | ILK | integrin linked kinase | 3611 |
| 726 | INIP | INTS3 and NABP interacting protein | 58493 |
| 727 | NUP37 | nucleoporin 37 | 79023 |
| 728 | RMDN3 | regulator of microtubule dynamics 3 | 55177 |
| 729 | CHD4 | chromodomain helicase DNA binding protein 4 | 1108 |
| 730 | RCN1 | reticulocalbin 1 | 5954 |
| 731 | NCOA5 | nuclear receptor coactivator 5 | 57727 |
| 732 | ALDOA | aldolase, fructose-bisphosphate A | 226 |
| 733 | PLS3 | plastin 3 | 5358 |
| 734 | NDUFB11 | NADH:ubiquinone oxidoreductase subunit B11 | 54539 |
| 735 | TIMM10 | translocase of inner mitochondrial membrane 10 | 26519 |
| 736 | FLG | filaggrin | 2312 |
| 737 | NUDCD1 | NudC domain containing 1 | 84955 |
| 738 | MRPS9 | mitochondrial ribosomal protein S9 | 64965 |
| 739 | PDLIM1 | PDZ and LIM domain 1 | 9124 |
| 740 | PRMT1 | protein arginine methyltransferase 1 | 3276 |
| 741 | PTCD3 | pentatricopeptide repeat domain 3 | 55037 |
| 742 | S100A6 | S100 calcium binding protein A6 | 6277 |
| 743 | PTGES3 | prostaglandin E synthase 3 | 10728 |
| 744 | PRKAG1 | protein kinase AMP-activated non-catalytic subunit gamma 1 | 5571 |
| 745 | PRDX2 | peroxiredoxin 2 | 7001 |
| 746 | POLR2G | RNA polymerase II subunit G | 5436 |
| 747 | SLC4A7 | solute carrier family 4 member 7 | 9497 |
| 748 | SHMT2 | serine hydroxymethyltransferase 2 | 6472 |
| 749 | PSMA4 | proteasome subunit alpha 4 | 5685 |
| 750 | DTX3L | deltex E3 ubiquitin ligase 3L | 151636 |
| 751 | VAT1 | vesicle amine transport 1 | 10493 |
| 752 | PLPBP | pyridoxal phosphate binding protein | 11212 |
| 753 | MAOA | monoamine oxidase A | 4128 |
| 754 | GNL3 | G protein nucleolar 3 | 26354 |
| 755 | CHMP1A | charged multivesicular body protein 1A | 5119 |
| 756 | PSMB5 | proteasome subunit beta 5 | 5693 |
| 757 | MDH1 | malate dehydrogenase 1 | 4190 |
| 758 | CARS2 | cysteinyl-tRNA synthetase 2, mitochondrial | 79587 |
| 759 | ATP1B3 | ATPase Na+/K+ transporting subunit beta 3 | 483 |
| 760 | GNA13 | G protein subunit alpha 13 | 10672 |
| 761 | ALB | albumin | 213 |
| 762 | ALYREF | Aly/REF export factor | 10189 |
| 763 | FAM210A | family with sequence similarity 210 member A | 125228 |
| 764 | HSP90AB1 | heat shock protein 90 alpha family class B member 1 | 3326 |
| 765 | NMT1 | N-myristoyltransferase 1 | 4836 |
| 766 | DNAJC8 | DnaJ heat shock protein family (Hsp40) member C8 | 22826 |
| 767 | UBE2L3 | ubiquitin conjugating enzyme E2 L3 | 7332 |
| 768 | UBE2H | ubiquitin conjugating enzyme E2 H | 7328 |
| 769 | AP1B1 | adaptor related protein complex 1 subunit beta 1 | 162 |
| 770 | HNRNPA2B1 | heterogeneous nuclear ribonucleoprotein A2/B1 | 3181 |
| 771 | GRN | granulin precursor | 2896 |
| 772 | PDIA5 | protein disulfide isomerase family A member 5 | 10954 |
| 773 | ATP6V1B2 | ATPase H+ transporting V1 subunit B2 | 526 |
| 774 | AMY1A | amylase, alpha 1A (salivary) | 276 |
| 775 | SRPRA | SRP receptor subunit alpha | 6734 |
| 776 | NOL3 | nucleolar protein 3 | 8996 |
| 777 | MBNL1 | muscleblind like splicing regulator 1 | 4154 |
| 778 | PSMD9 | proteasome 26S subunit, non-ATPase 9 | 5715 |
| 779 | TMEM209 | transmembrane protein 209 | 84928 |
| 780 | HDDC2 | HD domain containing 2 | 51020 |
| 781 | RPL3 | ribosomal protein L3 | 6122 |
| 782 | TRIP4 | thyroid hormone receptor interactor 4 | 9325 |
| 783 | HADH | hydroxyacyl-CoA dehydrogenase | 3033 |
| 784 | RAD23B | RAD23 homolog B, nucleotide excision repair protein | 5887 |
| 785 | SUCLG1 | succinate-CoA ligase alpha subunit | 8802 |
| 786 | NOSIP | nitric oxide synthase interacting protein | 51070 |
| 787 | PFDN6 | prefoldin subunit 6 | 10471 |
| 788 | ALDH1A3 | aldehyde dehydrogenase 1 family member A3 | 220 |
| 789 | AFG3L2 | AFG3 like matrix AAA peptidase subunit 2 | 10939 |
| 790 | TBCA | tubulin folding cofactor A | 6902 |
| 791 | ST13 | ST13, Hsp70 interacting protein | 6767 |
| 792 | ESYT1 | extended synaptotagmin 1 | 23344 |
| 793 | CDV3 | CDV3 homolog | 55573 |
| 794 | SMC1A | structural maintenance of chromosomes 1A | 8243 |
| 795 | XAB2 | XPA binding protein 2 | 56949 |
| 796 | PCYOX1 | prenylcysteine oxidase 1 | 51449 |
| 797 | CST3 | cystatin C | 1471 |
| 798 | MRPL47 | mitochondrial ribosomal protein L47 | 57129 |
| 799 | WDR12 | WD repeat domain 12 | 55759 |
| 800 | CKAP2 | cytoskeleton associated protein 2 | 26586 |
| 801 | COMMD2 | COMM domain containing 2 | 51122 |
| 802 | XPO1 | exportin 1 | 7514 |
| 803 | TCOF1 | treacle ribosome biogenesis factor 1 | 6949 |
| 804 | ATP5F1A | ATP synthase F1 subunit alpha | 498 |
| 805 | ACBD3 | acyl-CoA binding domain containing 3 | 64746 |
| 806 | FDXR | ferredoxin reductase | 2232 |
| 807 | NIF3L1 | NGG1 interacting factor 3 like 1 | 60491 |
| 808 | SNU13 | small nuclear ribonucleoprotein 13 | 4809 |
| 809 | STX6 | syntaxin 6 | 10228 |
| 810 | JCHAIN | joining chain of multimeric IgA and IgM | 3512 |
| 811 | COG7 | component of oligomeric golgi complex 7 | 91949 |
| 812 | PBDC1 | polysaccharide biosynthesis domain containing 1 | 51260 |
| 813 | FAH | fumarylacetoacetate hydrolase | 2184 |
| 814 | AZGP1 | alpha-2-glycoprotein 1, zinc-binding | 563 |
| 815 | ADGRE5 | adhesion G protein-coupled receptor E5 | 976 |
| 816 | ENO1 | enolase 1 | 2023 |
| 817 | USP24 | ubiquitin specific peptidase 24 | 23358 |
| 818 | CARM1 | coactivator associated arginine methyltransferase 1 | 10498 |
| 819 | YWHAE | tyrosine 3-monooxygenase/tryptophan 5-monooxygenase activation protein epsilon | 7531 |
| 820 | FOXRED1 | FAD dependent oxidoreductase domain containing 1 | 55572 |
| 821 | SGTB | small glutamine rich tetratricopeptide repeat containing beta | 54557 |
| 822 | CES2 | carboxylesterase 2 | 8824 |
| 823 | PSMD14 | proteasome 26S subunit, non-ATPase 14 | 10213 |
| 824 | UBE2V2 | ubiquitin conjugating enzyme E2 V2 | 7336 |
| 825 | MX1 | MX dynamin like GTPase 1 | 4599 |
| 826 | MVD | mevalonate diphosphate decarboxylase | 4597 |
| 827 | ACTN4 | actinin alpha 4 | 81 |
| 828 | MCAM | melanoma cell adhesion molecule | 4162 |
| 829 | HPRT1 | hypoxanthine phosphoribosyltransferase 1 | 3251 |
| 830 | TMEM205 | transmembrane protein 205 | 374882 |
| 831 | USP5 | ubiquitin specific peptidase 5 | 8078 |
| 832 | PDXDC1 | pyridoxal dependent decarboxylase domain containing 1 | 23042 |
| 833 | SDE2 | SDE2 telomere maintenance homolog | 163859 |
| 834 | NUP50 | nucleoporin 50 | 10762 |
| 835 | RPRD1B | regulation of nuclear pre-mRNA domain containing 1B | 58490 |
| 836 | CYB5R3 | cytochrome b5 reductase 3 | 1727 |
| 837 | EBNA1BP2 | EBNA1 binding protein 2 | 10969 |
| 838 | NXN | nucleoredoxin | 64359 |
| 839 | MRPL45 | mitochondrial ribosomal protein L45 | 84311 |
| 840 | ASL | argininosuccinate lyase | 435 |
| 841 | RBM10 | RNA binding motif protein 10 | 8241 |
| 842 | TMED4 | transmembrane p24 trafficking protein 4 | 222068 |
| 843 | TUT7 | terminal uridylyl transferase 7 | 79670 |
| 844 | DSG2 | desmoglein 2 | 1829 |
| 845 | GNG12 | G protein subunit gamma 12 | 55970 |
| 846 | RNF20 | ring finger protein 20 | 56254 |
| 847 | UQCRQ | ubiquinol-cytochrome c reductase complex III subunit VII | 27089 |
| 848 | PTPN6 | protein tyrosine phosphatase, non-receptor type 6 | 5777 |
| 849 | GPALPP1 | GPALPP motifs containing 1 | 55425 |
| 850 | DNAJB2 | DnaJ heat shock protein family (Hsp40) member B2 | 3300 |
| 851 | RPA3 | replication protein A3 | 6119 |
| 852 | EMC1 | ER membrane protein complex subunit 1 | 23065 |
| 853 | ENSA | endosulfine alpha | 2029 |
| 854 | KIF2A | kinesin family member 2A | 3796 |
| 855 | UAP1 | UDP-N-acetylglucosamine pyrophosphorylase 1 | 6675 |
| 856 | GSDME | gasdermin E | 1687 |
| 857 | DLST | dihydrolipoamide S-succinyltransferase | 1743 |
| 858 | MBOAT7 | membrane bound O-acyltransferase domain containing 7 | 79143 |
| 859 | MMUT | methylmalonyl-CoA mutase | 4594 |
| 860 | CD109 | CD109 molecule | 135228 |
| 861 | CCT6A | chaperonin containing TCP1 subunit 6A | 908 |
| 862 | STT3A | STT3A, catalytic subunit of the oligosaccharyltransferase complex | 3703 |
| 863 | NHLRC2 | NHL repeat containing 2 | 374354 |
| 864 | VAPA | VAMP associated protein A | 9218 |
| 865 | CBX3 | chromobox 3 | 11335 |
| 866 | IRF6 | interferon regulatory factor 6 | 3664 |
| 867 | APOE | apolipoprotein E | 348 |
| 868 | SCAF4 | SR-related CTD associated factor 4 | 57466 |
| 869 | BAIAP2L1 | BAI1 associated protein 2 like 1 | 55971 |
| 870 | HSPA2 | heat shock protein family A (Hsp70) member 2 | 3306 |
| 871 | HEXB | hexosaminidase subunit beta | 3074 |
| 872 | YBX3 | Y-box binding protein 3 | 8531 |
| 873 | ARHGEF18 | Rho/Rac guanine nucleotide exchange factor 18 | 23370 |
| 874 | SMC2 | structural maintenance of chromosomes 2 | 10592 |
| 875 | RP2 | RP2, ARL3 GTPase activating protein | 6102 |
| 876 | EFTUD2 | elongation factor Tu GTP binding domain containing 2 | 9343 |
| 877 | THOC3 | THO complex 3 | 84321 |
| 878 | OAT | ornithine aminotransferase | 4942 |
| 879 | RDH11 | retinol dehydrogenase 11 | 51109 |
| 880 | HDGF | heparin binding growth factor | 3068 |
| 881 | DNPEP | aspartyl aminopeptidase | 23549 |
| 882 | DYNC1H1 | dynein cytoplasmic 1 heavy chain 1 | 1778 |
| 883 | HMGB2 | high mobility group box 2 | 3148 |
| 884 | IFIT1 | interferon induced protein with tetratricopeptide repeats 1 | 3434 |
| 885 | SEC24B | SEC24 homolog B, COPII coat complex component | 10427 |
| 886 | GTF2F1 | general transcription factor IIF subunit 1 | 2962 |
| 887 | AK2 | adenylate kinase 2 | 204 |
| 888 | FH | fumarate hydratase | 2271 |
| 889 | MAPK14 | mitogen-activated protein kinase 14 | 1432 |
| 890 | ASCC3 | activating signal cointegrator 1 complex subunit 3 | 10973 |
| 891 | HSPA14 | heat shock protein family A (Hsp70) member 14 | 51182 |
| 892 | UBTF | upstream binding transcription factor | 7343 |
| 893 | HINT2 | histidine triad nucleotide binding protein 2 | 84681 |
| 894 | SCP2 | sterol carrier protein 2 | 6342 |
| 895 | HIBADH | 3-hydroxyisobutyrate dehydrogenase | 11112 |
| 896 | IDH3A | isocitrate dehydrogenase 3 (NAD(+)) alpha | 3419 |
| 897 | IPO4 | importin 4 | 79711 |
| 898 | RBMS1 | RNA binding motif single stranded interacting protein 1 | 5937 |
| 899 | PTPRF | protein tyrosine phosphatase, receptor type F | 5792 |
| 900 | ACACA | acetyl-CoA carboxylase alpha | 31 |
| 901 | STAU1 | staufen double-stranded RNA binding protein 1 | 6780 |
| 902 | DYNLRB1 | dynein light chain roadblock-type 1 | 83658 |
| 903 | UBQLN4 | ubiquilin 4 | 56893 |
| 904 | PARP1 | poly(ADP-ribose) polymerase 1 | 142 |
| 905 | MSH2 | mutS homolog 2 | 4436 |
| 906 | HDGFL2 | HDGF like 2 | 84717 |
| 907 | SRPK2 | SRSF protein kinase 2 | 6733 |
| 908 | GGCT | gamma-glutamylcyclotransferase | 79017 |
| 909 | KDSR | 3-ketodihydrosphingosine reductase | 2531 |
| 910 | UBE2M | ubiquitin conjugating enzyme E2 M | 9040 |
| 911 | SYNE2 | spectrin repeat containing nuclear envelope protein 2 | 23224 |
| 912 | TFG | TRK-fused gene | 10342 |
| 913 | SEC22B | SEC22 homolog B, vesicle trafficking protein (gene/pseudogene) | 9554 |
| 914 | DDX5 | DEAD-box helicase 5 | 1655 |
| 915 | COX5A | cytochrome c oxidase subunit 5A | 9377 |
| 916 | IGF2BP2 | insulin like growth factor 2 mRNA binding protein 2 | 10644 |
| 917 | UFC1 | ubiquitin-fold modifier conjugating enzyme 1 | 51506 |
| 918 | FAM120A | family with sequence similarity 120A | 23196 |
| 919 | SLC16A3 | solute carrier family 16 member 3 | 9123 |
| 920 | IST1 | IST1, ESCRT-III associated factor | 9798 |
| 921 | LARP4B | La ribonucleoprotein domain family member 4B | 23185 |
| 922 | ITPK1 | inositol-tetrakisphosphate 1-kinase | 3705 |
| 923 | PPP1R8 | protein phosphatase 1 regulatory subunit 8 | 5511 |
| 924 | GINS2 | GINS complex subunit 2 | 51659 |
| 925 | CCAR1 | cell division cycle and apoptosis regulator 1 | 55749 |
| 926 | MYO18A | myosin XVIIIA | 399687 |
| 927 | NIT2 | nitrilase family member 2 | 56954 |
| 928 | SIRPA | signal regulatory protein alpha | 140885 |
| 929 | CKB | creatine kinase B | 1152 |
| 930 | RPN2 | ribophorin II | 6185 |
| 931 | MAX | MYC associated factor X | 4149 |
| 932 | B2M | beta-2-microglobulin | 567 |
| 933 | GYG1 | glycogenin 1 | 2992 |
| 934 | CBFB | core-binding factor subunit beta | 865 |
| 935 | HBB | hemoglobin subunit beta | 3043 |
| 936 | PSMB4 | proteasome subunit beta 4 | 5692 |
| 937 | MGAT1 | mannosyl (alpha-1,3-)-glycoprotein beta-1,2-N-acetylglucosaminyltransferase | 4245 |
| 938 | HSD17B12 | hydroxysteroid 17-beta dehydrogenase 12 | 51144 |
| 939 | RTRAF | RNA transcription, translation and transport factor | 51637 |
| 940 | PSME3 | proteasome activator subunit 3 | 10197 |
| 941 | HSPA1B | heat shock protein family A (Hsp70) member 1B | 3304 |
| 942 | DUS2 | dihydrouridine synthase 2 | 54920 |
| 943 | ACO2 | aconitase 2 | 50 |
| 944 | ZC3H4 | zinc finger CCCH-type containing 4 | 23211 |
| 945 | ZPR1 | ZPR1 zinc finger | 8882 |
| 946 | DSTN | destrin, actin depolymerizing factor | 11034 |
| 947 | NT5C3A | 5'-nucleotidase, cytosolic IIIA | 51251 |
| 948 | AIMP1 | aminoacyl tRNA synthetase complex interacting multifunctional protein 1 | 9255 |
| 949 | COLGALT1 | collagen beta(1-O)galactosyltransferase 1 | 79709 |
| 950 | RRP8 | ribosomal RNA processing 8 | 23378 |
| 951 | UBR4 | ubiquitin protein ligase E3 component n-recognin 4 | 23352 |
| 952 | DSC1 | desmocollin 1 | 1823 |
| 953 | JUNB | JunB proto-oncogene, AP-1 transcription factor subunit | 3726 |
| 954 | POLR2H | RNA polymerase II subunit H | 5437 |
| 955 | OTULIN | OTU deubiquitinase with linear linkage specificity | 90268 |
| 956 | GNPDA1 | glucosamine-6-phosphate deaminase 1 | 10007 |
| 957 | PSMA3 | proteasome subunit alpha 3 | 5684 |
| 958 | SPINT2 | serine peptidase inhibitor, Kunitz type 2 | 10653 |
| 959 | SMARCC2 | SWI/SNF related, matrix associated, actin dependent regulator of chromatin subfamily c member 2 | 6601 |
| 960 | NAP1L1 | nucleosome assembly protein 1 like 1 | 4673 |
| 961 | HSPD1 | heat shock protein family D (Hsp60) member 1 | 3329 |
| 962 | SLC25A24 | solute carrier family 25 member 24 | 29957 |
| 963 | DDX50 | DExD-box helicase 50 | 79009 |
| 964 | PYCR1 | pyrroline-5-carboxylate reductase 1 | 5831 |
| 965 | WDR36 | WD repeat domain 36 | 134430 |
| 966 | SSBP1 | single stranded DNA binding protein 1 | 6742 |
| 967 | UBAP2L | ubiquitin associated protein 2 like | 9898 |
| 968 | SH3BGRL3 | SH3 domain binding glutamate rich protein like 3 | 83442 |
| 969 | ATXN10 | ataxin 10 | 25814 |
| 970 | HDAC2 | histone deacetylase 2 | 3066 |
| 971 | NUP58 | nucleoporin 58 | 9818 |
| 972 | ANP32E | acidic nuclear phosphoprotein 32 family member E | 81611 |
| 973 | TAGLN2 | transgelin 2 | 8407 |
| 974 | FDPS | farnesyl diphosphate synthase | 2224 |
| 975 | MAGOHB | mago homolog B, exon junction complex subunit | 55110 |
| 976 | DEK | DEK proto-oncogene | 7913 |
| 977 | KRT78 | keratin 78 | 196374 |
| 978 | NFYC | nuclear transcription factor Y subunit gamma | 4802 |
| 979 | MYH10 | myosin heavy chain 10 | 4628 |
| 980 | RHOT2 | ras homolog family member T2 | 89941 |
| 981 | SARS2 | seryl-tRNA synthetase 2, mitochondrial | 54938 |
| 982 | STAM | signal transducing adaptor molecule | 8027 |
| 983 | YWHAH | tyrosine 3-monooxygenase/tryptophan 5-monooxygenase activation protein eta | 7533 |
| 984 | TMED10 | transmembrane p24 trafficking protein 10 | 10972 |
| 985 | JPT2 | Jupiter microtubule associated homolog 2 | 90861 |
| 986 | DKC1 | dyskerin pseudouridine synthase 1 | 1736 |
| 987 | FKBP10 | FKBP prolyl isomerase 10 | 60681 |
| 988 | LTA4H | leukotriene A4 hydrolase | 4048 |
| 989 | CDC37 | cell division cycle 37 | 11140 |
| 990 | LANCL2 | LanC like 2 | 55915 |
| 991 | SLC25A6 | solute carrier family 25 member 6 | 293 |
| 992 | MYBBP1A | MYB binding protein 1a | 10514 |
| 993 | MPHOSPH10 | M-phase phosphoprotein 10 | 10199 |
| 994 | UBE2K | ubiquitin conjugating enzyme E2 K | 3093 |
| 995 | PDCD11 | programmed cell death 11 | 22984 |
| 996 | CHMP2B | charged multivesicular body protein 2B | 25978 |
| 997 | SLC12A4 | solute carrier family 12 member 4 | 6560 |
| 998 | CDK6 | cyclin dependent kinase 6 | 1021 |
| 999 | CDC5L | cell division cycle 5 like | 988 |
| 1000 | ACP1 | acid phosphatase 1 | 52 |
| 1001 | VDAC1 | voltage dependent anion channel 1 | 7416 |
| 1002 | SRSF3 | serine and arginine rich splicing factor 3 | 6428 |
| 1003 | DNAJC13 | DnaJ heat shock protein family (Hsp40) member C13 | 23317 |
| 1004 | RANBP1 | RAN binding protein 1 | 5902 |
| 1005 | CTTNBP2NL | CTTNBP2 N-terminal like | 55917 |
| 1006 | PDCD10 | programmed cell death 10 | 11235 |
| 1007 | MYO1C | myosin IC | 4641 |
| 1008 | NUP133 | nucleoporin 133 | 55746 |
| 1009 | EIF3C | eukaryotic translation initiation factor 3 subunit C | 8663 |
| 1010 | TMSB10 | thymosin beta 10 | 9168 |
| 1011 | XPO5 | exportin 5 | 57510 |
| 1012 | EGFR | epidermal growth factor receptor | 1956 |
| 1013 | GCDH | glutaryl-CoA dehydrogenase | 2639 |
| 1014 | LAMB3 | laminin subunit beta 3 | 3914 |
| 1015 | DENR | density regulated re-initiation and release factor | 8562 |
| 1016 | SPAG7 | sperm associated antigen 7 | 9552 |
| 1017 | SORD | sorbitol dehydrogenase | 6652 |
| 1018 | FABP5 | fatty acid binding protein 5 | 2171 |
| 1019 | LGALS1 | galectin 1 | 3956 |
| 1020 | PHB | prohibitin | 5245 |
| 1021 | RPLP0 | ribosomal protein lateral stalk subunit P0 | 6175 |
| 1022 | DNM1L | dynamin 1 like | 10059 |
| 1023 | SLC9A3R1 | SLC9A3 regulator 1 | 9368 |
| 1024 | ILKAP | ILK associated serine/threonine phosphatase | 80895 |
| 1025 | OCIAD1 | OCIA domain containing 1 | 54940 |
| 1026 | RANBP2 | RAN binding protein 2 | 5903 |
| 1027 | FBXO2 | F-box protein 2 | 26232 |
| 1028 | GOLM1 | golgi membrane protein 1 | 51280 |
| 1029 | NUP205 | nucleoporin 205 | 23165 |
| 1030 | NAPG | NSF attachment protein gamma | 8774 |
| 1031 | IAH1 | isoamyl acetate hydrolyzing esterase 1 (putative) | 285148 |
| 1032 | CZIB | CXXC motif containing zinc binding protein | 54987 |
| 1033 | SDAD1 | SDA1 domain containing 1 | 55153 |
| 1034 | TBC1D2 | TBC1 domain family member 2 | 55357 |
| 1035 | CERS6 | ceramide synthase 6 | 253782 |
| 1036 | STAT1 | signal transducer and activator of transcription 1 | 6772 |
| 1037 | PIN1 | peptidylprolyl cis/trans isomerase, NIMA-interacting 1 | 5300 |
| 1038 | GPHN | gephyrin | 10243 |
| 1039 | CCT5 | chaperonin containing TCP1 subunit 5 | 22948 |
| 1040 | RAB3GAP1 | RAB3 GTPase activating protein catalytic subunit 1 | 22930 |
| 1041 | CTBP1 | C-terminal binding protein 1 | 1487 |
| 1042 | GSTM3 | glutathione S-transferase mu 3 | 2947 |
| 1043 | ANXA1 | annexin A1 | 301 |
| 1044 | IKBIP | IKBKB interacting protein | 121457 |
| 1045 | TPM1 | tropomyosin 1 | 7168 |
| 1046 | NBN | nibrin | 4683 |
| 1047 | CSTA | cystatin A | 1475 |
| 1048 | DSP | desmoplakin | 1832 |
| 1049 | TAP1 | transporter 1, ATP binding cassette subfamily B member | 6890 |
| 1050 | EIF4A3 | eukaryotic translation initiation factor 4A3 | 9775 |
| 1051 | ISY1 | ISY1 splicing factor homolog | 57461 |
| 1052 | UPP1 | uridine phosphorylase 1 | 7378 |
| 1053 | CAV1 | caveolin 1 | 857 |
| 1054 | RBM28 | RNA binding motif protein 28 | 55131 |
| 1055 | CSK | C-terminal Src kinase | 1445 |
| 1056 | CCT2 | chaperonin containing TCP1 subunit 2 | 10576 |
| 1057 | POLR2A | RNA polymerase II subunit A | 5430 |
| 1058 | ERAP1 | endoplasmic reticulum aminopeptidase 1 | 51752 |
| 1059 | GSTT2 | glutathione S-transferase theta 2 (gene/pseudogene) | 2953 |
| 1060 | SART3 | spliceosome associated factor 3, U4/U6 recycling protein | 9733 |
| 1061 | SNX9 | sorting nexin 9 | 51429 |
| 1062 | MRPL9 | mitochondrial ribosomal protein L9 | 65005 |
| 1063 | HSPA4L | heat shock protein family A (Hsp70) member 4 like | 22824 |
| 1064 | APMAP | adipocyte plasma membrane associated protein | 57136 |
| 1065 | IFIT3 | interferon induced protein with tetratricopeptide repeats 3 | 3437 |
| 1066 | PITPNA | phosphatidylinositol transfer protein alpha | 5306 |
| 1067 | SRP9 | signal recognition particle 9 | 6726 |
| 1068 | UQCRB | ubiquinol-cytochrome c reductase binding protein | 7381 |
| 1069 | SAAL1 | serum amyloid A like 1 | 113174 |
| 1070 | SLC25A5 | solute carrier family 25 member 5 | 292 |
| 1071 | FOSL2 | FOS like 2, AP-1 transcription factor subunit | 2355 |
| 1072 | UBAP2 | ubiquitin associated protein 2 | 55833 |
| 1073 | ZNF326 | zinc finger protein 326 | 284695 |
| 1074 | DCTN4 | dynactin subunit 4 | 51164 |
| 1075 | TAX1BP1 | Tax1 binding protein 1 | 8887 |
| 1076 | RNF40 | ring finger protein 40 | 9810 |
| 1077 | EPHA2 | EPH receptor A2 | 1969 |
| 1078 | SF3B2 | splicing factor 3b subunit 2 | 10992 |
| 1079 | LYZ | lysozyme | 4069 |
| 1080 | ESYT2 | extended synaptotagmin 2 | 57488 |
| 1081 | UTRN | utrophin | 7402 |
| 1082 | DDX42 | DEAD-box helicase 42 | 11325 |
| 1083 | PMPCA | peptidase, mitochondrial processing alpha subunit | 23203 |
| 1084 | SON | SON DNA binding protein | 6651 |
| 1085 | TFIP11 | tuftelin interacting protein 11 | 24144 |
| 1086 | PITPNB | phosphatidylinositol transfer protein beta | 23760 |
| 1087 | BAG3 | BCL2 associated athanogene 3 | 9531 |
| 1088 | RIC8A | RIC8 guanine nucleotide exchange factor A | 60626 |
| 1089 | SPECC1L | sperm antigen with calponin homology and coiled-coil domains 1 like | 23384 |
| 1090 | GNAI1 | G protein subunit alpha i1 | 2770 |
| 1091 | ATP5F1B | ATP synthase F1 subunit beta | 506 |
| 1092 | PFDN4 | prefoldin subunit 4 | 5203 |
| 1093 | HGH1 | HGH1 homolog | 51236 |
| 1094 | PICALM | phosphatidylinositol binding clathrin assembly protein | 8301 |
| 1095 | SDF2L1 | stromal cell derived factor 2 like 1 | 23753 |
| 1096 | DYNC1I2 | dynein cytoplasmic 1 intermediate chain 2 | 1781 |
| 1097 | S100A7 | S100 calcium binding protein A7 | 6278 |
| 1098 | DRG1 | developmentally regulated GTP binding protein 1 | 4733 |
| 1099 | QRICH1 | glutamine rich 1 | 54870 |
| 1100 | MAD2L1 | mitotic arrest deficient 2 like 1 | 4085 |
| 1101 | SLC16A1 | solute carrier family 16 member 1 | 6566 |
| 1102 | LDHA | lactate dehydrogenase A | 3939 |
| 1103 | EWSR1 | EWS RNA binding protein 1 | 2130 |
| 1104 | LSM1 | LSM1 homolog, mRNA degradation associated | 27257 |
| 1105 | MPRIP | myosin phosphatase Rho interacting protein | 23164 |
| 1106 | SNRPN | small nuclear ribonucleoprotein polypeptide N | 6638 |
| 1107 | FBXL18 | F-box and leucine rich repeat protein 18 | 80028 |
| 1108 | HMOX2 | heme oxygenase 2 | 3163 |
| 1109 | GNAI2 | G protein subunit alpha i2 | 2771 |
| 1110 | TRAP1 | TNF receptor associated protein 1 | 10131 |
| 1111 | CNIH4 | cornichon family AMPA receptor auxiliary protein 4 | 29097 |
| 1112 | SEC23B | Sec23 homolog B, coat complex II component | 10483 |
| 1113 | EIF4A2 | eukaryotic translation initiation factor 4A2 | 1974 |
| 1114 | LSM8 | LSM8 homolog, U6 small nuclear RNA associated | 51691 |
| 1115 | MRPS16 | mitochondrial ribosomal protein S16 | 51021 |
| 1116 | ERP44 | endoplasmic reticulum protein 44 | 23071 |
| 1117 | LNPEP | leucyl and cystinyl aminopeptidase | 4012 |
| 1118 | EIF5 | eukaryotic translation initiation factor 5 | 1983 |
| 1119 | EMC8 | ER membrane protein complex subunit 8 | 10328 |
| 1120 | TMBIM1 | transmembrane BAX inhibitor motif containing 1 | 64114 |
| 1121 | GRHPR | glyoxylate and hydroxypyruvate reductase | 9380 |
| 1122 | PNPO | pyridoxamine 5'-phosphate oxidase | 55163 |
| 1123 | RBMX | RNA binding motif protein X-linked | 27316 |
| 1124 | ATP6V1C1 | ATPase H+ transporting V1 subunit C1 | 528 |
| 1125 | RACK1 | receptor for activated C kinase 1 | 10399 |
| 1126 | SPATS2L | spermatogenesis associated serine rich 2 like | 26010 |
| 1127 | PDAP1 | PDGFA associated protein 1 | 11333 |
| 1128 | KPNA6 | karyopherin subunit alpha 6 | 23633 |
| 1129 | TRIP10 | thyroid hormone receptor interactor 10 | 9322 |
| 1130 | TPD52L1 | TPD52 like 1 | 7164 |
| 1131 | PPME1 | protein phosphatase methylesterase 1 | 51400 |
| 1132 | CHMP4A | charged multivesicular body protein 4A | 29082 |
| 1133 | NDUFV1 | NADH:ubiquinone oxidoreductase core subunit V1 | 4723 |
| 1134 | FLNA | filamin A | 2316 |
| 1135 | RPS7 | ribosomal protein S7 | 6201 |
| 1136 | PPP2R1A | protein phosphatase 2 scaffold subunit Aalpha | 5518 |
| 1137 | RPL35A | ribosomal protein L35a | 6165 |
| 1138 | PPIE | peptidylprolyl isomerase E | 10450 |
| 1139 | IGHG1 | immunoglobulin heavy constant gamma 1 (G1m marker) | 3500 |
| 1140 | SOAT1 | sterol O-acyltransferase 1 | 6646 |
| 1141 | CNDP2 | carnosine dipeptidase 2 | 55748 |
| 1142 | RPL8 | ribosomal protein L8 | 6132 |
| 1143 | NUDT21 | nudix hydrolase 21 | 11051 |
| 1144 | COASY | Coenzyme A synthase | 80347 |
| 1145 | ALDH9A1 | aldehyde dehydrogenase 9 family member A1 | 223 |
| 1146 | PDLIM2 | PDZ and LIM domain 2 | 64236 |
| 1147 | RAB11B | RAB11B, member RAS oncogene family | 9230 |
| 1148 | ACAT2 | acetyl-CoA acetyltransferase 2 | 39 |
| 1149 | RBM3 | RNA binding motif protein 3 | 5935 |
| 1150 | CAND1 | cullin associated and neddylation dissociated 1 | 55832 |
| 1151 | PEF1 | penta-EF-hand domain containing 1 | 553115 |
| 1152 | FAM83H | family with sequence similarity 83 member H | 286077 |
| 1153 | DDX46 | DEAD-box helicase 46 | 9879 |
| 1154 | DAD1 | defender against cell death 1 | 1603 |
| 1155 | GIGYF2 | GRB10 interacting GYF protein 2 | 26058 |
| 1156 | EIF1AX | eukaryotic translation initiation factor 1A X-linked | 1964 |
| 1157 | OXA1L | OXA1L, mitochondrial inner membrane protein | 5018 |
| 1158 | YBX1 | Y-box binding protein 1 | 4904 |
| 1159 | TMEM40 | transmembrane protein 40 | 55287 |
| 1160 | LMNB1 | lamin B1 | 4001 |
| 1161 | COX7A2 | cytochrome c oxidase subunit 7A2 | 1347 |
| 1162 | RPL31 | ribosomal protein L31 | 6160 |
| 1163 | RPL18A | ribosomal protein L18a | 6142 |
| 1164 | WDR1 | WD repeat domain 1 | 9948 |
| 1165 | POLR2C | RNA polymerase II subunit C | 5432 |
| 1166 | TGFBI | transforming growth factor beta induced | 7045 |
| 1167 | MIPEP | mitochondrial intermediate peptidase | 4285 |
| 1168 | EI24 | EI24, autophagy associated transmembrane protein | 9538 |
| 1169 | EHD4 | EH domain containing 4 | 30844 |
| 1170 | MRPL38 | mitochondrial ribosomal protein L38 | 64978 |
| 1171 | WNK1 | WNK lysine deficient protein kinase 1 | 65125 |
| 1172 | DDX58 | DExD/H-box helicase 58 | 23586 |
| 1173 | CDK9 | cyclin dependent kinase 9 | 1025 |
| 1174 | CD2BP2 | CD2 cytoplasmic tail binding protein 2 | 10421 |
| 1175 | CCDC9 | coiled-coil domain containing 9 | 26093 |
| 1176 | OGT | O-linked N-acetylglucosamine (GlcNAc) transferase | 8473 |
| 1177 | NIPSNAP1 | nipsnap homolog 1 | 8508 |
| 1178 | SEC13 | SEC13 homolog, nuclear pore and COPII coat complex component | 6396 |
| 1179 | THYN1 | thymocyte nuclear protein 1 | 29087 |
| 1180 | ZYX | zyxin | 7791 |
| 1181 | SLC25A4 | solute carrier family 25 member 4 | 291 |
| 1182 | CTSD | cathepsin D | 1509 |
| 1183 | DDX39B | DExD-box helicase 39B | 7919 |
| 1184 | PYGB | glycogen phosphorylase B | 5834 |
| 1185 | POLR2E | RNA polymerase II subunit E | 5434 |
| 1186 | PSMB7 | proteasome subunit beta 7 | 5695 |
| 1187 | ME2 | malic enzyme 2 | 4200 |
| 1188 | IPO11 | importin 11 | 51194 |
| 1189 | HBS1L | HBS1 like translational GTPase | 10767 |
| 1190 | AHCY | adenosylhomocysteinase | 191 |
| 1191 | MCM4 | minichromosome maintenance complex component 4 | 4173 |
| 1192 | TBCB | tubulin folding cofactor B | 1155 |
| 1193 | MRPL49 | mitochondrial ribosomal protein L49 | 740 |
| 1194 | TBRG4 | transforming growth factor beta regulator 4 | 9238 |
| 1195 | SEC62 | SEC62 homolog, preprotein translocation factor | 7095 |
| 1196 | DPP9 | dipeptidyl peptidase 9 | 91039 |
| 1197 | RSRC1 | arginine and serine rich coiled-coil 1 | 51319 |
| 1198 | HDHD5 | haloacid dehalogenase like hydrolase domain containing 5 | 27440 |
| 1199 | EML2 | EMAP like 2 | 24139 |
| 1200 | PACS1 | phosphofurin acidic cluster sorting protein 1 | 55690 |
| 1201 | MTHFD1L | methylenetetrahydrofolate dehydrogenase (NADP+ dependent) 1 like | 25902 |
| 1202 | ACSL4 | acyl-CoA synthetase long chain family member 4 | 2182 |
| 1203 | LUC7L2 | LUC7 like 2, pre-mRNA splicing factor | 51631 |
| 1204 | NDUFA8 | NADH:ubiquinone oxidoreductase subunit A8 | 4702 |
| 1205 | PLAA | phospholipase A2 activating protein | 9373 |
| 1206 | PPIH | peptidylprolyl isomerase H | 10465 |
| 1207 | PROSER2 | proline and serine rich 2 | 254427 |
| 1208 | NCEH1 | neutral cholesterol ester hydrolase 1 | 57552 |
| 1209 | CRNKL1 | crooked neck pre-mRNA splicing factor 1 | 51340 |
| 1210 | MYH9 | myosin heavy chain 9 | 4627 |
| 1211 | RAE1 | ribonucleic acid export 1 | 8480 |
| 1212 | PSMB1 | proteasome subunit beta 1 | 5689 |
| 1213 | ADSL | adenylosuccinate lyase | 158 |
| 1214 | TOMM34 | translocase of outer mitochondrial membrane 34 | 10953 |
| 1215 | SLC38A1 | solute carrier family 38 member 1 | 81539 |
| 1216 | NUDT9 | nudix hydrolase 9 | 53343 |
| 1217 | STAT3 | signal transducer and activator of transcription 3 | 6774 |
| 1218 | DNAJA1 | DnaJ heat shock protein family (Hsp40) member A1 | 3301 |
| 1219 | SAP30BP | SAP30 binding protein | 29115 |
| 1220 | AGA | aspartylglucosaminidase | 175 |
| 1221 | ATP6V0D1 | ATPase H+ transporting V0 subunit d1 | 9114 |
| 1222 | RPS2 | ribosomal protein S2 | 6187 |
| 1223 | ZC3H15 | zinc finger CCCH-type containing 15 | 55854 |
| 1224 | USP9X | ubiquitin specific peptidase 9 X-linked | 8239 |
| 1225 | ENDOD1 | endonuclease domain containing 1 | 23052 |
| 1226 | LRRFIP1 | LRR binding FLII interacting protein 1 | 9208 |
| 1227 | PSMB3 | proteasome subunit beta 3 | 5691 |
| 1228 | S100A16 | S100 calcium binding protein A16 | 140576 |
| 1229 | MCM7 | minichromosome maintenance complex component 7 | 4176 |
| 1230 | DHCR24 | 24-dehydrocholesterol reductase | 1718 |
| 1231 | SFN | stratifin | 2810 |
| 1232 | DSC3 | desmocollin 3 | 1825 |
| 1233 | CDK11B | cyclin dependent kinase 11B | 984 |
| 1234 | SERBP1 | SERPINE1 mRNA binding protein 1 | 26135 |
| 1235 | ATP5IF1 | ATP synthase inhibitory factor subunit 1 | 93974 |
| 1236 | HLA-B | major histocompatibility complex, class I, B | 3106 |
| 1237 | EIF4A1 | eukaryotic translation initiation factor 4A1 | 1973 |
| 1238 | ACTN1 | actinin alpha 1 | 87 |
| 1239 | NUDT16L1 | nudix hydrolase 16 like 1 | 84309 |
| 1240 | RPL18 | ribosomal protein L18 | 6141 |
| 1241 | LMAN2 | lectin, mannose binding 2 | 10960 |
| 1242 | SNAP23 | synaptosome associated protein 23 | 8773 |
| 1243 | SNX18 | sorting nexin 18 | 112574 |
| 1244 | QKI | QKI, KH domain containing RNA binding | 9444 |
| 1245 | EIF4G1 | eukaryotic translation initiation factor 4 gamma 1 | 1981 |
| 1246 | MCFD2 | multiple coagulation factor deficiency 2 | 90411 |
| 1247 | SETD7 | SET domain containing 7, histone lysine methyltransferase | 80854 |
| 1248 | TRIM29 | tripartite motif containing 29 | 23650 |
| 1249 | VPS33A | VPS33A, CORVET/HOPS core subunit | 65082 |
| 1250 | IGBP1 | immunoglobulin binding protein 1 | 3476 |
| 1251 | CISD1 | CDGSH iron sulfur domain 1 | 55847 |
| 1252 | DDB1 | damage specific DNA binding protein 1 | 1642 |
| 1253 | RAB3GAP2 | RAB3 GTPase activating non-catalytic protein subunit 2 | 25782 |
| 1254 | COPS6 | COP9 signalosome subunit 6 | 10980 |
| 1255 | ECI2 | enoyl-CoA delta isomerase 2 | 10455 |
| 1256 | MRPL28 | mitochondrial ribosomal protein L28 | 10573 |
| 1257 | BID | BH3 interacting domain death agonist | 637 |
| 1258 | FARSB | phenylalanyl-tRNA synthetase subunit beta | 10056 |
| 1259 | RNASEH2A | ribonuclease H2 subunit A | 10535 |
| 1260 | TRIM28 | tripartite motif containing 28 | 10155 |
| 1261 | BROX | BRO1 domain and CAAX motif containing | 148362 |
| 1262 | EMD | emerin | 2010 |
| 1263 | DIS3 | DIS3 homolog, exosome endoribonuclease and 3'-5' exoribonuclease | 22894 |
| 1264 | HOOK1 | hook microtubule tethering protein 1 | 51361 |
| 1265 | TOMM40 | translocase of outer mitochondrial membrane 40 | 10452 |
| 1266 | CTNNB1 | catenin beta 1 | 1499 |
| 1267 | ALDH3A2 | aldehyde dehydrogenase 3 family member A2 | 224 |
| 1268 | ARHGEF2 | Rho/Rac guanine nucleotide exchange factor 2 | 9181 |
| 1269 | AIMP2 | aminoacyl tRNA synthetase complex interacting multifunctional protein 2 | 7965 |
| 1270 | CLIC1 | chloride intracellular channel 1 | 1192 |
| 1271 | TPP1 | tripeptidyl peptidase 1 | 1200 |
| 1272 | TYMP | thymidine phosphorylase | 1890 |
| 1273 | RCC1 | regulator of chromosome condensation 1 | 1104 |
| 1274 | KPNA3 | karyopherin subunit alpha 3 | 3839 |
| 1275 | MCM3 | minichromosome maintenance complex component 3 | 4172 |
| 1276 | LMAN1 | lectin, mannose binding 1 | 3998 |
| 1277 | VIRMA | vir like m6A methyltransferase associated | 25962 |
| 1278 | CBX5 | chromobox 5 | 23468 |
| 1279 | CNN3 | calponin 3 | 1266 |
| 1280 | PBK | PDZ binding kinase | 55872 |
| 1281 | EEF1D | eukaryotic translation elongation factor 1 delta | 1936 |
| 1282 | TMEM214 | transmembrane protein 214 | 54867 |
| 1283 | ENY2 | ENY2, transcription and export complex 2 subunit | 56943 |
| 1284 | STK10 | serine/threonine kinase 10 | 6793 |
| 1285 | MYL6B | myosin light chain 6B | 140465 |
| 1286 | MARK2 | microtubule affinity regulating kinase 2 | 2011 |
| 1287 | CALU | calumenin | 813 |
| 1288 | HNRNPAB | heterogeneous nuclear ribonucleoprotein A/B | 3182 |
| 1289 | SRSF6 | serine and arginine rich splicing factor 6 | 6431 |
| 1290 | GCN1 | GCN1, eIF2 alpha kinase activator homolog | 10985 |
| 1291 | MTHFD2 | methylenetetrahydrofolate dehydrogenase (NADP+ dependent) 2, methenyltetrahydrofolate cyclohydrolase | 10797 |
| 1292 | NAA50 | N(alpha)-acetyltransferase 50, NatE catalytic subunit | 80218 |
| 1293 | PLXNB2 | plexin B2 | 23654 |
| 1294 | BLVRB | biliverdin reductase B | 645 |
| 1295 | MRPS35 | mitochondrial ribosomal protein S35 | 60488 |
| 1296 | CLIC3 | chloride intracellular channel 3 | 9022 |
| 1297 | NDUFA10 | NADH:ubiquinone oxidoreductase subunit A10 | 4705 |
| 1298 | RANBP3 | RAN binding protein 3 | 8498 |
| 1299 | GRPEL1 | GrpE like 1, mitochondrial | 80273 |
| 1300 | ATP6V1E1 | ATPase H+ transporting V1 subunit E1 | 529 |
| 1301 | CAD | carbamoyl-phosphate synthetase 2, aspartate transcarbamylase, and dihydroorotase | 790 |
| 1302 | IQGAP1 | IQ motif containing GTPase activating protein 1 | 8826 |
| 1303 | TBC1D10B | TBC1 domain family member 10B | 26000 |
| 1304 | PAFAH1B3 | platelet activating factor acetylhydrolase 1b catalytic subunit 3 | 5050 |
| 1305 | PTBP1 | polypyrimidine tract binding protein 1 | 5725 |
| 1306 | TTLL12 | tubulin tyrosine ligase like 12 | 23170 |
| 1307 | PPP2R1B | protein phosphatase 2 scaffold subunit Abeta | 5519 |
| 1308 | HSPA8 | heat shock protein family A (Hsp70) member 8 | 3312 |
| 1309 | TBC1D5 | TBC1 domain family member 5 | 9779 |
| 1310 | ARPC5 | actin related protein 2/3 complex subunit 5 | 10092 |
| 1311 | LRRC8E | leucine rich repeat containing 8 VRAC subunit E | 80131 |
| 1312 | RPL38 | ribosomal protein L38 | 6169 |
| 1313 | PSMD2 | proteasome 26S subunit, non-ATPase 2 | 5708 |
| 1314 | RAN | RAN, member RAS oncogene family | 5901 |
| 1315 | SUB1 | SUB1 homolog, transcriptional regulator | 10923 |
| 1316 | ERP29 | endoplasmic reticulum protein 29 | 10961 |
| 1317 | DHX29 | DExH-box helicase 29 | 54505 |
| 1318 | RBM39 | RNA binding motif protein 39 | 9584 |
| 1319 | MRPL39 | mitochondrial ribosomal protein L39 | 54148 |
| 1320 | ECI1 | enoyl-CoA delta isomerase 1 | 1632 |
| 1321 | NDUFS2 | NADH:ubiquinone oxidoreductase core subunit S2 | 4720 |
| 1322 | RPS9 | ribosomal protein S9 | 6203 |
| 1323 | IL18 | interleukin 18 | 3606 |
| 1324 | GRWD1 | glutamate rich WD repeat containing 1 | 83743 |
| 1325 | STK26 | serine/threonine kinase 26 | 51765 |
| 1326 | EHBP1 | EH domain binding protein 1 | 23301 |
| 1327 | SNX6 | sorting nexin 6 | 58533 |
| 1328 | SERPINH1 | serpin family H member 1 | 871 |
| 1329 | MRPS36 | mitochondrial ribosomal protein S36 | 92259 |
| 1330 | NDUFB4 | NADH:ubiquinone oxidoreductase subunit B4 | 4710 |
| 1331 | TIMM8A | translocase of inner mitochondrial membrane 8A | 1678 |
| 1332 | EIF3J | eukaryotic translation initiation factor 3 subunit J | 8669 |
| 1333 | ANLN | anillin actin binding protein | 54443 |
| 1334 | TARDBP | TAR DNA binding protein | 23435 |
| 1335 | HNRNPH1 | heterogeneous nuclear ribonucleoprotein H1 | 3187 |
| 1336 | INF2 | inverted formin, FH2 and WH2 domain containing | 64423 |
| 1337 | OPTN | optineurin | 10133 |
| 1338 | ITGB4 | integrin subunit beta 4 | 3691 |
| 1339 | STOML2 | stomatin like 2 | 30968 |
| 1340 | PSMB2 | proteasome subunit beta 2 | 5690 |
| 1341 | AP2A2 | adaptor related protein complex 2 subunit alpha 2 | 161 |
| 1342 | CLUH | clustered mitochondria homolog | 23277 |
| 1343 | NAE1 | NEDD8 activating enzyme E1 subunit 1 | 8883 |
| 1344 | RRM2 | ribonucleotide reductase regulatory subunit M2 | 6241 |
| 1345 | DCTN2 | dynactin subunit 2 | 10540 |
| 1346 | MAPRE1 | microtubule associated protein RP/EB family member 1 | 22919 |
| 1347 | THOC1 | THO complex 1 | 9984 |
| 1348 | ZC3HAV1 | zinc finger CCCH-type containing, antiviral 1 | 56829 |
| 1349 | ARGLU1 | arginine and glutamate rich 1 | 55082 |
| 1350 | RALY | RALY heterogeneous nuclear ribonucleoprotein | 22913 |
| 1351 | KRT7 | keratin 7 | 3855 |
| 1352 | IMPDH2 | inosine monophosphate dehydrogenase 2 | 3615 |
| 1353 | NUDC | nuclear distribution C, dynein complex regulator | 10726 |
| 1354 | UPF1 | UPF1, RNA helicase and ATPase | 5976 |
| 1355 | MTPN | myotrophin | 136319 |
| 1356 | TRIP11 | thyroid hormone receptor interactor 11 | 9321 |
| 1357 | STK24 | serine/threonine kinase 24 | 8428 |
| 1358 | CYB5R2 | cytochrome b5 reductase 2 | 51700 |
| 1359 | KIF5B | kinesin family member 5B | 3799 |
| 1360 | SRP19 | signal recognition particle 19 | 6728 |
| 1361 | SF3A1 | splicing factor 3a subunit 1 | 10291 |
| 1362 | NMI | N-myc and STAT interactor | 9111 |
| 1363 | CMBL | carboxymethylenebutenolidase homolog | 134147 |
| 1364 | TOMM22 | translocase of outer mitochondrial membrane 22 | 56993 |
| 1365 | NUP43 | nucleoporin 43 | 348995 |
| 1366 | CNOT3 | CCR4-NOT transcription complex subunit 3 | 4849 |
| 1367 | HEBP2 | heme binding protein 2 | 23593 |
| 1368 | CDC42 | cell division cycle 42 | 998 |
| 1369 | ZC3H14 | zinc finger CCCH-type containing 14 | 79882 |
| 1370 | XPOT | exportin for tRNA | 11260 |
| 1371 | A2M | alpha-2-macroglobulin | 2 |
| 1372 | CLTB | clathrin light chain B | 1212 |
| 1373 | CAPZB | capping actin protein of muscle Z-line subunit beta | 832 |
| 1374 | CAP1 | cyclase associated actin cytoskeleton regulatory protein 1 | 10487 |
| 1375 | ACOT9 | acyl-CoA thioesterase 9 | 23597 |
| 1376 | ADK | adenosine kinase | 132 |
| 1377 | PDLIM5 | PDZ and LIM domain 5 | 10611 |
| 1378 | MPST | mercaptopyruvate sulfurtransferase | 4357 |
| 1379 | TPT1 | tumor protein, translationally-controlled 1 | 7178 |
| 1380 | PFKP | phosphofructokinase, platelet | 5214 |
| 1381 | POLR2D | RNA polymerase II subunit D | 5433 |
| 1382 | PCBP2 | poly(rC) binding protein 2 | 5094 |
| 1383 | EIF2B3 | eukaryotic translation initiation factor 2B subunit gamma | 8891 |
| 1384 | EXOSC7 | exosome component 7 | 23016 |
| 1385 | LSM14B | LSM family member 14B | 149986 |
| 1386 | VAMP3 | vesicle associated membrane protein 3 | 9341 |
| 1387 | NAA10 | N(alpha)-acetyltransferase 10, NatA catalytic subunit | 8260 |
| 1388 | BRCC3 | BRCA1/BRCA2-containing complex subunit 3 | 79184 |
| 1389 | UCK2 | uridine-cytidine kinase 2 | 7371 |
| 1390 | IGF2BP3 | insulin like growth factor 2 mRNA binding protein 3 | 10643 |
| 1391 | MRPL11 | mitochondrial ribosomal protein L11 | 65003 |
| 1392 | DNAJC3 | DnaJ heat shock protein family (Hsp40) member C3 | 5611 |
| 1393 | SYMPK | symplekin | 8189 |
| 1394 | TOLLIP | toll interacting protein | 54472 |
| 1395 | TPI1 | triosephosphate isomerase 1 | 7167 |
| 1396 | DYNLL1 | dynein light chain LC8-type 1 | 8655 |
| 1397 | ARMC6 | armadillo repeat containing 6 | 93436 |
| 1398 | RPL36AL | ribosomal protein L36a like | 6166 |
| 1399 | XPO7 | exportin 7 | 23039 |
| 1400 | FMR1 | fragile X mental retardation 1 | 2332 |
| 1401 | METAP1 | methionyl aminopeptidase 1 | 23173 |
| 1402 | TUBG1 | tubulin gamma 1 | 7283 |
| 1403 | MRPL13 | mitochondrial ribosomal protein L13 | 28998 |
| 1404 | HLA-C | major histocompatibility complex, class I, C | 3107 |
| 1405 | ACBD5 | acyl-CoA binding domain containing 5 | 91452 |
| 1406 | NOB1 | NIN1 (RPN12) binding protein 1 homolog | 28987 |
| 1407 | GEMIN6 | gem nuclear organelle associated protein 6 | 79833 |
| 1408 | F11R | F11 receptor | 50848 |
| 1409 | GPATCH8 | G-patch domain containing 8 | 23131 |
| 1410 | NUP62 | nucleoporin 62 | 23636 |
| 1411 | SLC25A11 | solute carrier family 25 member 11 | 8402 |
| 1412 | PABPC4 | poly(A) binding protein cytoplasmic 4 | 8761 |
| 1413 | HDLBP | high density lipoprotein binding protein | 3069 |
| 1414 | RING1 | ring finger protein 1 | 6015 |
| 1415 | EPS15 | epidermal growth factor receptor pathway substrate 15 | 2060 |
| 1416 | MAP7D1 | MAP7 domain containing 1 | 55700 |
| 1417 | TOMM70 | translocase of outer mitochondrial membrane 70 | 9868 |
| 1418 | FKBP4 | FKBP prolyl isomerase 4 | 2288 |
| 1419 | SLC6A15 | solute carrier family 6 member 15 | 55117 |
| 1420 | IPO5 | importin 5 | 3843 |
| 1421 | TARBP2 | TARBP2, RISC loading complex RNA binding subunit | 6895 |
| 1422 | CPSF1 | cleavage and polyadenylation specific factor 1 | 29894 |
| 1423 | GPATCH4 | G-patch domain containing 4 | 54865 |
| 1424 | MAN2A1 | mannosidase alpha class 2A member 1 | 4124 |
| 1425 | ARHGDIA | Rho GDP dissociation inhibitor alpha | 396 |
| 1426 | MICAL3 | microtubule associated monooxygenase, calponin and LIM domain containing 3 | 57553 |
| 1427 | PDLIM4 | PDZ and LIM domain 4 | 8572 |
| 1428 | RPS28 | ribosomal protein S28 | 6234 |
| 1429 | AAMDC | adipogenesis associated Mth938 domain containing | 28971 |
| 1430 | BAG2 | BCL2 associated athanogene 2 | 9532 |
| 1431 | BRK1 | BRICK1, SCAR/WAVE actin nucleating complex subunit | 55845 |
| 1432 | HOOK3 | hook microtubule tethering protein 3 | 84376 |
| 1433 | EPN2 | epsin 2 | 22905 |
| 1434 | YKT6 | YKT6 v-SNARE homolog | 10652 |
| 1435 | ABCF3 | ATP binding cassette subfamily F member 3 | 55324 |
| 1436 | HM13 | histocompatibility minor 13 | 81502 |
| 1437 | USP7 | ubiquitin specific peptidase 7 | 7874 |
| 1438 | ECM1 | extracellular matrix protein 1 | 1893 |
| 1439 | CA2 | carbonic anhydrase 2 | 760 |
| 1440 | SDHB | succinate dehydrogenase complex iron sulfur subunit B | 6390 |
| 1441 | NDUFB3 | NADH:ubiquinone oxidoreductase subunit B3 | 4709 |
| 1442 | PHLDB2 | pleckstrin homology like domain family B member 2 | 90102 |
| 1443 | PARP14 | poly(ADP-ribose) polymerase family member 14 | 54625 |
| 1444 | PSMD11 | proteasome 26S subunit, non-ATPase 11 | 5717 |
| 1445 | HP1BP3 | heterochromatin protein 1 binding protein 3 | 50809 |
| 1446 | LIMA1 | LIM domain and actin binding 1 | 51474 |
| 1447 | RNPS1 | RNA binding protein with serine rich domain 1 | 10921 |
| 1448 | FKBP11 | FKBP prolyl isomerase 11 | 51303 |
| 1449 | ERLIN1 | ER lipid raft associated 1 | 10613 |
| 1450 | PPID | peptidylprolyl isomerase D | 5481 |
| 1451 | FXN | frataxin | 2395 |
| 1452 | MMP14 | matrix metallopeptidase 14 | 4323 |
| 1453 | DDX18 | DEAD-box helicase 18 | 8886 |
| 1454 | ATXN3 | ataxin 3 | 4287 |
| 1455 | STX8 | syntaxin 8 | 9482 |
| 1456 | SERPINE1 | serpin family E member 1 | 5054 |
| 1457 | TMEM165 | transmembrane protein 165 | 55858 |
| 1458 | TIMM13 | translocase of inner mitochondrial membrane 13 | 26517 |
| 1459 | ATIC | 5-aminoimidazole-4-carboxamide ribonucleotide formyltransferase/IMP cyclohydrolase | 471 |
| 1460 | SEC24C | SEC24 homolog C, COPII coat complex component | 9632 |
| 1461 | SOWAHC | sosondowah ankyrin repeat domain family member C | 65124 |
| 1462 | NCAPD2 | non-SMC condensin I complex subunit D2 | 9918 |
| 1463 | PABPC1 | poly(A) binding protein cytoplasmic 1 | 26986 |
| 1464 | VCP | valosin containing protein | 7415 |
| 1465 | PYCARD | PYD and CARD domain containing | 29108 |
| 1466 | CTSB | cathepsin B | 1508 |
| 1467 | TWF2 | twinfilin actin binding protein 2 | 11344 |
| 1468 | NAA15 | N(alpha)-acetyltransferase 15, NatA auxiliary subunit | 80155 |
| 1469 | ATP6V1D | ATPase H+ transporting V1 subunit D | 51382 |
| 1470 | PANK4 | pantothenate kinase 4 | 55229 |
| 1471 | CORO1B | coronin 1B | 57175 |
| 1472 | MEAK7 | MTOR associated protein, eak-7 homolog | 57707 |
| 1473 | GLS | glutaminase | 2744 |
| 1474 | SUN1 | Sad1 and UNC84 domain containing 1 | 23353 |
| 1475 | VPS29 | VPS29, retromer complex component | 51699 |
| 1476 | NUP155 | nucleoporin 155 | 9631 |
| 1477 | PNPT1 | polyribonucleotide nucleotidyltransferase 1 | 87178 |
| 1478 | FKBP2 | FKBP prolyl isomerase 2 | 2286 |
| 1479 | APOO | apolipoprotein O | 79135 |
| 1480 | LARP1 | La ribonucleoprotein domain family member 1 | 23367 |
| 1481 | DTYMK | deoxythymidylate kinase | 1841 |
| 1482 | CRLF3 | cytokine receptor like factor 3 | 51379 |
| 1483 | CHMP6 | charged multivesicular body protein 6 | 79643 |
| 1484 | CLTC | clathrin heavy chain | 1213 |
| 1485 | URI1 | URI1, prefoldin like chaperone | 8725 |
| 1486 | COPA | coatomer protein complex subunit alpha | 1314 |
| 1487 | COX4I1 | cytochrome c oxidase subunit 4I1 | 1327 |
| 1488 | PSMD13 | proteasome 26S subunit, non-ATPase 13 | 5719 |
| 1489 | ATP5PF | ATP synthase peripheral stalk subunit F6 | 522 |
| 1490 | RBBP4 | RB binding protein 4, chromatin remodeling factor | 5928 |
| 1491 | PPP4R3A | protein phosphatase 4 regulatory subunit 3A | 55671 |
| 1492 | NAPA | NSF attachment protein alpha | 8775 |
| 1493 | FLNB | filamin B | 2317 |
| 1494 | UBQLN2 | ubiquilin 2 | 29978 |
| 1495 | NECAP2 | NECAP endocytosis associated 2 | 55707 |
| 1496 | RPS13 | ribosomal protein S13 | 6207 |
| 1497 | LAS1L | LAS1 like, ribosome biogenesis factor | 81887 |
| 1498 | LGALS3 | galectin 3 | 3958 |
| 1499 | ATP5PD | ATP synthase peripheral stalk subunit d | 10476 |
| 1500 | UBA1 | ubiquitin like modifier activating enzyme 1 | 7317 |
| 1501 | NPC1 | NPC intracellular cholesterol transporter 1 | 4864 |
| 1502 | MATR3 | matrin 3 | 9782 |
| 1503 | AGRN | agrin | 375790 |
| 1504 | MTMR6 | myotubularin related protein 6 | 9107 |
| 1505 | SRRT | serrate, RNA effector molecule | 51593 |
| 1506 | PPP2CB | protein phosphatase 2 catalytic subunit beta | 5516 |
| 1507 | SYNCRIP | synaptotagmin binding cytoplasmic RNA interacting protein | 10492 |
| 1508 | CEP170B | centrosomal protein 170B | 283638 |
| 1509 | MIA2 | MIA SH3 domain ER export factor 2 | 4253 |
| 1510 | SNRPA1 | small nuclear ribonucleoprotein polypeptide A' | 6627 |
| 1511 | MAP2K1 | mitogen-activated protein kinase kinase 1 | 5604 |
| 1512 | USP10 | ubiquitin specific peptidase 10 | 9100 |
| 1513 | RTCB | RNA 2',3'-cyclic phosphate and 5'-OH ligase | 51493 |
| 1514 | ALDH16A1 | aldehyde dehydrogenase 16 family member A1 | 126133 |
| 1515 | UBE2D2 | ubiquitin conjugating enzyme E2 D2 | 7322 |
| 1516 | CSTF3 | cleavage stimulation factor subunit 3 | 1479 |
| 1517 | OGDH | oxoglutarate dehydrogenase | 4967 |
| 1518 | CYB5B | cytochrome b5 type B | 80777 |
| 1519 | SPR | sepiapterin reductase | 6697 |
| 1520 | RAB2A | RAB2A, member RAS oncogene family | 5862 |
| 1521 | MAP2K2 | mitogen-activated protein kinase kinase 2 | 5605 |
| 1522 | HCCS | holocytochrome c synthase | 3052 |
| 1523 | BRD4 | bromodomain containing 4 | 23476 |
| 1524 | NPEPPS | aminopeptidase puromycin sensitive | 9520 |
| 1525 | RAMAC | RNA guanine-7 methyltransferase activating subunit | 83640 |
| 1526 | KCTD12 | potassium channel tetramerization domain containing 12 | 115207 |
| 1527 | GORASP2 | golgi reassembly stacking protein 2 | 26003 |
| 1528 | EIF3H | eukaryotic translation initiation factor 3 subunit H | 8667 |
| 1529 | ATL3 | atlastin GTPase 3 | 25923 |
| 1530 | CD46 | CD46 molecule | 4179 |
| 1531 | UBXN1 | UBX domain protein 1 | 51035 |
| 1532 | PSAP | prosaposin | 5660 |
| 1533 | PMPCB | peptidase, mitochondrial processing beta subunit | 9512 |
| 1534 | VAPB | VAMP associated protein B and C | 9217 |
| 1535 | MRPL19 | mitochondrial ribosomal protein L19 | 9801 |
| 1536 | IPO9 | importin 9 | 55705 |
| 1537 | BCAP31 | B cell receptor associated protein 31 | 10134 |
| 1538 | TTC37 | tetratricopeptide repeat domain 37 | 9652 |
| 1539 | PDCD6IP | programmed cell death 6 interacting protein | 10015 |
| 1540 | TALDO1 | transaldolase 1 | 6888 |
| 1541 | FTL | ferritin light chain | 2512 |
| 1542 | EXOSC10 | exosome component 10 | 5394 |
| 1543 | MCMBP | minichromosome maintenance complex binding protein | 79892 |
| 1544 | PPA2 | pyrophosphatase (inorganic) 2 | 27068 |
| 1545 | NPC2 | NPC intracellular cholesterol transporter 2 | 10577 |
| 1546 | EVPL | envoplakin | 2125 |
| 1547 | RPL34 | ribosomal protein L34 | 6164 |
| 1548 | FSCN1 | fascin actin-bundling protein 1 | 6624 |
| 1549 | CALR | calreticulin | 811 |
| 1550 | TERF2IP | TERF2 interacting protein | 54386 |
| 1551 | LRRC59 | leucine rich repeat containing 59 | 55379 |
| 1552 | TRMT10C | tRNA methyltransferase 10C, mitochondrial RNase P subunit | 54931 |
| 1553 | F3 | coagulation factor III, tissue factor | 2152 |
| 1554 | SP100 | SP100 nuclear antigen | 6672 |
| 1555 | GPN1 | GPN-loop GTPase 1 | 11321 |
| 1556 | EIF2S1 | eukaryotic translation initiation factor 2 subunit alpha | 1965 |
| 1557 | NOMO1 | NODAL modulator 1 | 23420 |
| 1558 | CLPX | caseinolytic mitochondrial matrix peptidase chaperone subunit | 10845 |
| 1559 | VDAC2 | voltage dependent anion channel 2 | 7417 |
| 1560 | NOP56 | NOP56 ribonucleoprotein | 10528 |
| 1561 | ANP32A | acidic nuclear phosphoprotein 32 family member A | 8125 |
| 1562 | HUWE1 | HECT, UBA and WWE domain containing 1, E3 ubiquitin protein ligase | 10075 |
| 1563 | AHSA1 | activator of HSP90 ATPase activity 1 | 10598 |
| 1564 | SFPQ | splicing factor proline and glutamine rich | 6421 |
| 1565 | CUX1 | cut like homeobox 1 | 1523 |
| 1566 | SCRIB | scribble planar cell polarity protein | 23513 |
| 1567 | NFKB1 | nuclear factor kappa B subunit 1 | 4790 |
| 1568 | TBCD | tubulin folding cofactor D | 6904 |
| 1569 | THOC5 | THO complex 5 | 8563 |
| 1570 | RPL24 | ribosomal protein L24 | 6152 |
| 1571 | DFFA | DNA fragmentation factor subunit alpha | 1676 |
| 1572 | PM20D2 | peptidase M20 domain containing 2 | 135293 |
| 1573 | WDHD1 | WD repeat and HMG-box DNA binding protein 1 | 11169 |
| 1574 | ANXA4 | annexin A4 | 307 |
| 1575 | CTSC | cathepsin C | 1075 |
| 1576 | ADIRF | adipogenesis regulatory factor | 10974 |
| 1577 | AP1G1 | adaptor related protein complex 1 subunit gamma 1 | 164 |
| 1578 | ITCH | itchy E3 ubiquitin protein ligase | 83737 |
| 1579 | SEC23IP | SEC23 interacting protein | 11196 |
| 1580 | PITHD1 | PITH domain containing 1 | 57095 |
| 1581 | MTDH | metadherin | 92140 |
| 1582 | ST14 | suppression of tumorigenicity 14 | 6768 |
| 1583 | RPSA | ribosomal protein SA | 3921 |
| 1584 | IDH3B | isocitrate dehydrogenase 3 (NAD(+)) beta | 3420 |
| 1585 | KIAA2013 | KIAA2013 | 90231 |
| 1586 | RPL37A | ribosomal protein L37a | 6168 |
| 1587 | DYNC1LI2 | dynein cytoplasmic 1 light intermediate chain 2 | 1783 |
| 1588 | ISYNA1 | inositol-3-phosphate synthase 1 | 51477 |
| 1589 | HEXIM1 | HEXIM P-TEFb complex subunit 1 | 10614 |
| 1590 | RAB1A | RAB1A, member RAS oncogene family | 5861 |
| 1591 | CORO1C | coronin 1C | 23603 |
| 1592 | PPP6R3 | protein phosphatase 6 regulatory subunit 3 | 55291 |
| 1593 | CPSF4 | cleavage and polyadenylation specific factor 4 | 10898 |
| 1594 | KTN1 | kinectin 1 | 3895 |
| 1595 | ARPC3 | actin related protein 2/3 complex subunit 3 | 10094 |
| 1596 | SERPINB1 | serpin family B member 1 | 1992 |
| 1597 | RABGGTA | Rab geranylgeranyltransferase subunit alpha | 5875 |
| 1598 | CHTF18 | chromosome transmission fidelity factor 18 | 63922 |
| 1599 | FXR1 | FMR1 autosomal homolog 1 | 8087 |
| 1600 | MSN | moesin | 4478 |
| 1601 | AP3D1 | adaptor related protein complex 3 subunit delta 1 | 8943 |
| 1602 | PSMD5 | proteasome 26S subunit, non-ATPase 5 | 5711 |
| 1603 | CD81 | CD81 molecule | 975 |
| 1604 | RIDA | reactive intermediate imine deaminase A homolog | 10247 |
| 1605 | IARS2 | isoleucyl-tRNA synthetase 2, mitochondrial | 55699 |
| 1606 | DERA | deoxyribose-phosphate aldolase | 51071 |
| 1607 | MGST3 | microsomal glutathione S-transferase 3 | 4259 |
| 1608 | ERO1A | endoplasmic reticulum oxidoreductase 1 alpha | 30001 |
| 1609 | CRYZ | crystallin zeta | 1429 |
| 1610 | GRSF1 | G-rich RNA sequence binding factor 1 | 2926 |
| 1611 | C11orf54 | chromosome 11 open reading frame 54 | 28970 |
| 1612 | FGB | fibrinogen beta chain | 2244 |
| 1613 | SRP72 | signal recognition particle 72 | 6731 |
| 1614 | NXT1 | nuclear transport factor 2 like export factor 1 | 29107 |
| 1615 | NDUFS8 | NADH:ubiquinone oxidoreductase core subunit S8 | 4728 |
| 1616 | NUP85 | nucleoporin 85 | 79902 |
| 1617 | UQCRC1 | ubiquinol-cytochrome c reductase core protein 1 | 7384 |
| 1618 | SRSF2 | serine and arginine rich splicing factor 2 | 6427 |
| 1619 | EIF4E | eukaryotic translation initiation factor 4E | 1977 |
| 1620 | TIMM9 | translocase of inner mitochondrial membrane 9 | 26520 |
| 1621 | LAMP2 | lysosomal associated membrane protein 2 | 3920 |
| 1622 | FKBP1A | FKBP prolyl isomerase 1A | 2280 |
| 1623 | FIP1L1 | factor interacting with PAPOLA and CPSF1 | 81608 |
| 1624 | PANX1 | pannexin 1 | 24145 |
| 1625 | RSRC2 | arginine and serine rich coiled-coil 2 | 65117 |
| 1626 | GAPDH | glyceraldehyde-3-phosphate dehydrogenase | 2597 |
| 1627 | UQCRFS1 | ubiquinol-cytochrome c reductase, Rieske iron-sulfur polypeptide 1 | 7386 |
| 1628 | PAWR | pro-apoptotic WT1 regulator | 5074 |
| 1629 | KLC3 | kinesin light chain 3 | 147700 |
| 1630 | PGK1 | phosphoglycerate kinase 1 | 5230 |
| 1631 | CDC42BPB | CDC42 binding protein kinase beta | 9578 |
| 1632 | CUL3 | cullin 3 | 8452 |
| 1633 | TRIM22 | tripartite motif containing 22 | 10346 |
| 1634 | CSRP1 | cysteine and glycine rich protein 1 | 1465 |
| 1635 | OAS2 | 2'-5'-oligoadenylate synthetase 2 | 4939 |
| 1636 | TIMMDC1 | translocase of inner mitochondrial membrane domain containing 1 | 51300 |
| 1637 | UCHL3 | ubiquitin C-terminal hydrolase L3 | 7347 |
| 1638 | SRP68 | signal recognition particle 68 | 6730 |
| 1639 | SEC61A1 | Sec61 translocon alpha 1 subunit | 29927 |
| 1640 | OSBPL3 | oxysterol binding protein like 3 | 26031 |
| 1641 | PLOD1 | procollagen-lysine,2-oxoglutarate 5-dioxygenase 1 | 5351 |
| 1642 | ITGA6 | integrin subunit alpha 6 | 3655 |
| 1643 | CAVIN1 | caveolae associated protein 1 | 284119 |
| 1644 | GIPC1 | GIPC PDZ domain containing family member 1 | 10755 |
| 1645 | THEM6 | thioesterase superfamily member 6 | 51337 |
| 1646 | ELAC2 | elaC ribonuclease Z 2 | 60528 |
| 1647 | LAMTOR1 | late endosomal/lysosomal adaptor, MAPK and MTOR activator 1 | 55004 |
| 1648 | KPNA2 | karyopherin subunit alpha 2 | 3838 |
| 1649 | S100A2 | S100 calcium binding protein A2 | 6273 |
| 1650 | HSPBP1 | HSPA (Hsp70) binding protein 1 | 23640 |
| 1651 | PTPN1 | protein tyrosine phosphatase, non-receptor type 1 | 5770 |
| 1652 | EDC4 | enhancer of mRNA decapping 4 | 23644 |
| 1653 | NDUFS3 | NADH:ubiquinone oxidoreductase core subunit S3 | 4722 |
| 1654 | GOLGA3 | golgin A3 | 2802 |
| 1655 | MRPS5 | mitochondrial ribosomal protein S5 | 64969 |
| 1656 | RPL27A | ribosomal protein L27a | 6157 |
| 1657 | TKT | transketolase | 7086 |
| 1658 | KLC2 | kinesin light chain 2 | 64837 |
| 1659 | PPT1 | palmitoyl-protein thioesterase 1 | 5538 |
| 1660 | MOB1A | MOB kinase activator 1A | 55233 |
| 1661 | PCNA | proliferating cell nuclear antigen | 5111 |
| 1662 | RPL7 | ribosomal protein L7 | 6129 |
| 1663 | COPS3 | COP9 signalosome subunit 3 | 8533 |
| 1664 | AGL | amylo-alpha-1, 6-glucosidase, 4-alpha-glucanotransferase | 178 |
| 1665 | RPL27 | ribosomal protein L27 | 6155 |
| 1666 | ACTR5 | actin related protein 5 | 79913 |
| 1667 | LBR | lamin B receptor | 3930 |
| 1668 | TOR1A | torsin family 1 member A | 1861 |
| 1669 | ABCE1 | ATP binding cassette subfamily E member 1 | 6059 |
| 1670 | ZNF185 | zinc finger protein 185 with LIM domain | 7739 |
| 1671 | NCAPH | non-SMC condensin I complex subunit H | 23397 |
| 1672 | ACOT7 | acyl-CoA thioesterase 7 | 11332 |
| 1673 | IK | IK cytokine | 3550 |
| 1674 | NDUFAB1 | NADH:ubiquinone oxidoreductase subunit AB1 | 4706 |
| 1675 | SAP18 | Sin3A associated protein 18 | 10284 |
| 1676 | EIF2B4 | eukaryotic translation initiation factor 2B subunit delta | 8890 |
| 1677 | PGLS | 6-phosphogluconolactonase | 25796 |
| 1678 | TXNL1 | thioredoxin like 1 | 9352 |
| 1679 | RPL21 | ribosomal protein L21 | 6144 |
| 1680 | DNAJC9 | DnaJ heat shock protein family (Hsp40) member C9 | 23234 |
| 1681 | STX12 | syntaxin 12 | 23673 |
| 1682 | PPIC | peptidylprolyl isomerase C | 5480 |
| 1683 | MYL6 | myosin light chain 6 | 4637 |
| 1684 | MRRF | mitochondrial ribosome recycling factor | 92399 |
| 1685 | PSMC4 | proteasome 26S subunit, ATPase 4 | 5704 |
| 1686 | TJP2 | tight junction protein 2 | 9414 |
| 1687 | NDUFB9 | NADH:ubiquinone oxidoreductase subunit B9 | 4715 |
| 1688 | TXNDC17 | thioredoxin domain containing 17 | 84817 |
| 1689 | PTGS1 | prostaglandin-endoperoxide synthase 1 | 5742 |
| 1690 | CD9 | CD9 molecule | 928 |
| 1691 | CCT4 | chaperonin containing TCP1 subunit 4 | 10575 |
| 1692 | C12orf10 | chromosome 12 open reading frame 10 | 60314 |
| 1693 | PABPN1 | poly(A) binding protein nuclear 1 | 8106 |
| 1694 | PTPN11 | protein tyrosine phosphatase, non-receptor type 11 | 5781 |
| 1695 | PAPOLA | poly(A) polymerase alpha | 10914 |
| 1696 | COMMD9 | COMM domain containing 9 | 29099 |
| 1697 | SDHA | succinate dehydrogenase complex flavoprotein subunit A | 6389 |
| 1698 | RELA | RELA proto-oncogene, NF-kB subunit | 5970 |
| 1699 | AKR1B1 | aldo-keto reductase family 1 member B | 231 |
| 1700 | AASDHPPT | aminoadipate-semialdehyde dehydrogenase-phosphopantetheinyl transferase | 60496 |
| 1701 | RPL5 | ribosomal protein L5 | 6125 |
| 1702 | S100A13 | S100 calcium binding protein A13 | 6284 |
| 1703 | NDUFB5 | NADH:ubiquinone oxidoreductase subunit B5 | 4711 |
| 1704 | NDUFA7 | NADH:ubiquinone oxidoreductase subunit A7 | 4701 |
| 1705 | PRRC2A | proline rich coiled-coil 2A | 7916 |
| 1706 | VPS26A | VPS26, retromer complex component A | 9559 |
| 1707 | PYM1 | PYM homolog 1, exon junction complex associated factor | 84305 |
| 1708 | NHP2 | NHP2 ribonucleoprotein | 55651 |
| 1709 | TOP1 | DNA topoisomerase I | 7150 |
| 1710 | HSD17B8 | hydroxysteroid 17-beta dehydrogenase 8 | 7923 |
| 1711 | MRPS27 | mitochondrial ribosomal protein S27 | 23107 |
| 1712 | FHL3 | four and a half LIM domains 3 | 2275 |
| 1713 | LAMC2 | laminin subunit gamma 2 | 3918 |
| 1714 | SLC25A1 | solute carrier family 25 member 1 | 6576 |
| 1715 | IGF2R | insulin like growth factor 2 receptor | 3482 |
| 1716 | EZR | ezrin | 7430 |
| 1717 | GPX4 | glutathione peroxidase 4 | 2879 |
| 1718 | EIF3D | eukaryotic translation initiation factor 3 subunit D | 8664 |
| 1719 | PHB2 | prohibitin 2 | 11331 |
| 1720 | MX2 | MX dynamin like GTPase 2 | 4600 |
| 1721 | ABRACL | ABRA C-terminal like | 58527 |
| 1722 | GCLM | glutamate-cysteine ligase modifier subunit | 2730 |
| 1723 | GBA | glucosylceramidase beta | 2629 |
| 1724 | POR | cytochrome p450 oxidoreductase | 5447 |
| 1725 | C20orf27 | chromosome 20 open reading frame 27 | 54976 |
| 1726 | SCYL1 | SCY1 like pseudokinase 1 | 57410 |
| 1727 | ARHGEF1 | Rho guanine nucleotide exchange factor 1 | 9138 |
| 1728 | HADHA | hydroxyacyl-CoA dehydrogenase trifunctional multienzyme complex subunit alpha | 3030 |
| 1729 | PFAS | phosphoribosylformylglycinamidine synthase | 5198 |
| 1730 | GNL2 | G protein nucleolar 2 | 29889 |
| 1731 | BTF3 | basic transcription factor 3 | 689 |
| 1732 | ACLY | ATP citrate lyase | 47 |
| 1733 | RCOR3 | REST corepressor 3 | 55758 |
| 1734 | MCM6 | minichromosome maintenance complex component 6 | 4175 |
| 1735 | TXLNA | taxilin alpha | 200081 |
| 1736 | LGALS3BP | galectin 3 binding protein | 3959 |
| 1737 | TPM2 | tropomyosin 2 | 7169 |
| 1738 | PPCS | phosphopantothenoylcysteine synthetase | 79717 |
| 1739 | HACD3 | 3-hydroxyacyl-CoA dehydratase 3 | 51495 |
| 1740 | FN1 | fibronectin 1 | 2335 |
| 1741 | SEC61B | Sec61 translocon beta subunit | 10952 |
| 1742 | BAX | BCL2 associated X, apoptosis regulator | 581 |
| 1743 | MMP9 | matrix metallopeptidase 9 | 4318 |
| 1744 | CORO1A | coronin 1A | 11151 |
| 1745 | PPIL3 | peptidylprolyl isomerase like 3 | 53938 |
| 1746 | ACOT2 | acyl-CoA thioesterase 2 | 10965 |
| 1747 | CCDC86 | coiled-coil domain containing 86 | 79080 |
| 1748 | WDR44 | WD repeat domain 44 | 54521 |
| 1749 | PRKAR1A | protein kinase cAMP-dependent type I regulatory subunit alpha | 5573 |
| 1750 | UBR7 | ubiquitin protein ligase E3 component n-recognin 7 (putative) | 55148 |
| 1751 | AP2M1 | adaptor related protein complex 2 subunit mu 1 | 1173 |
| 1752 | LEMD3 | LEM domain containing 3 | 23592 |
| 1753 | MRPL51 | mitochondrial ribosomal protein L51 | 51258 |
| 1754 | EIF2AK2 | eukaryotic translation initiation factor 2 alpha kinase 2 | 5610 |
| 1755 | TMCO1 | transmembrane and coiled-coil domains 1 | 54499 |
| 1756 | EIF2S3 | eukaryotic translation initiation factor 2 subunit gamma | 1968 |
| 1757 | SPAG9 | sperm associated antigen 9 | 9043 |
| 1758 | AP3S1 | adaptor related protein complex 3 subunit sigma 1 | 1176 |
| 1759 | LRRC8A | leucine rich repeat containing 8 VRAC subunit A | 56262 |
| 1760 | BCL10 | BCL10, immune signaling adaptor | 8915 |
| 1761 | TTC1 | tetratricopeptide repeat domain 1 | 7265 |
| 1762 | DDX27 | DEAD-box helicase 27 | 55661 |
| 1763 | NASP | nuclear autoantigenic sperm protein | 4678 |
| 1764 | CRTAP | cartilage associated protein | 10491 |
| 1765 | RPL14 | ribosomal protein L14 | 9045 |
| 1766 | TOR1AIP1 | torsin 1A interacting protein 1 | 26092 |
| 1767 | ARL8A | ADP ribosylation factor like GTPase 8A | 127829 |
| 1768 | SRSF9 | serine and arginine rich splicing factor 9 | 8683 |
| 1769 | NUDCD2 | NudC domain containing 2 | 134492 |
| 1770 | PML | promyelocytic leukemia | 5371 |
| 1771 | EIF2S2 | eukaryotic translation initiation factor 2 subunit beta | 8894 |
| 1772 | DPYSL5 | dihydropyrimidinase like 5 | 56896 |
| 1773 | CYB5R1 | cytochrome b5 reductase 1 | 51706 |
| 1774 | TOR1AIP2 | torsin 1A interacting protein 2 | 163590 |
| 1775 | NBR1 | NBR1, autophagy cargo receptor | 4077 |
| 1776 | EIF3L | eukaryotic translation initiation factor 3 subunit L | 51386 |
| 1777 | TMX1 | thioredoxin related transmembrane protein 1 | 81542 |
| 1778 | PXDN | peroxidasin | 7837 |
| 1779 | NSF | N-ethylmaleimide sensitive factor, vesicle fusing ATPase | 4905 |
| 1780 | EEF1B2 | eukaryotic translation elongation factor 1 beta 2 | 1933 |
| 1781 | TSNAX | translin associated factor X | 7257 |
| 1782 | PTPN12 | protein tyrosine phosphatase, non-receptor type 12 | 5782 |
| 1783 | GOLGA2 | golgin A2 | 2801 |
| 1784 | CD44 | CD44 molecule (Indian blood group) | 960 |
| 1785 | DDX1 | DEAD-box helicase 1 | 1653 |
| 1786 | TATDN1 | TatD DNase domain containing 1 | 83940 |
| 1787 | RAB3D | RAB3D, member RAS oncogene family | 9545 |
| 1788 | OLA1 | Obg like ATPase 1 | 29789 |
| 1789 | SEC16A | SEC16 homolog A, endoplasmic reticulum export factor | 9919 |
| 1790 | U2AF1 | U2 small nuclear RNA auxiliary factor 1 | 7307 |
| 1791 | BUB3 | BUB3, mitotic checkpoint protein | 9184 |
| 1792 | BZW2 | basic leucine zipper and W2 domains 2 | 28969 |
| 1793 | GMPS | guanine monophosphate synthase | 8833 |
| 1794 | RPL10 | ribosomal protein L10 | 6134 |
| 1795 | TSC22D4 | TSC22 domain family member 4 | 81628 |
| 1796 | FNDC3A | fibronectin type III domain containing 3A | 22862 |
| 1797 | ERGIC3 | ERGIC and golgi 3 | 51614 |
| 1798 | LYAR | Ly1 antibody reactive | 55646 |
| 1799 | ATP5PB | ATP synthase peripheral stalk-membrane subunit b | 515 |
| 1800 | RPS6 | ribosomal protein S6 | 6194 |
| 1801 | STT3B | STT3B, catalytic subunit of the oligosaccharyltransferase complex | 201595 |
| 1802 | TUBGCP2 | tubulin gamma complex associated protein 2 | 10844 |
| 1803 | SH3GLB2 | SH3 domain containing GRB2 like, endophilin B2 | 56904 |
| 1804 | METTL3 | methyltransferase like 3 | 56339 |
| 1805 | NOP58 | NOP58 ribonucleoprotein | 51602 |
| 1806 | SLC2A1 | solute carrier family 2 member 1 | 6513 |
| 1807 | CCDC50 | coiled-coil domain containing 50 | 152137 |
| 1808 | RPS4X | ribosomal protein S4 X-linked | 6191 |
| 1809 | AKR1C2 | aldo-keto reductase family 1 member C2 | 1646 |
| 1810 | LIG3 | DNA ligase 3 | 3980 |
| 1811 | SDCBP | syndecan binding protein | 6386 |
| 1812 | HNRNPUL2 | heterogeneous nuclear ribonucleoprotein U like 2 | 221092 |
| 1813 | SNX2 | sorting nexin 2 | 6643 |
| 1814 | NDRG3 | NDRG family member 3 | 57446 |
| 1815 | HNRNPA0 | heterogeneous nuclear ribonucleoprotein A0 | 10949 |
| 1816 | PDXK | pyridoxal kinase | 8566 |
| 1817 | ISG15 | ISG15 ubiquitin-like modifier | 9636 |
| 1818 | SLC35B2 | solute carrier family 35 member B2 | 347734 |
| 1819 | PAK1 | p21 (RAC1) activated kinase 1 | 5058 |
| 1820 | FUBP3 | far upstream element binding protein 3 | 8939 |
| 1821 | RDH14 | retinol dehydrogenase 14 | 57665 |
| 1822 | ECH1 | enoyl-CoA hydratase 1 | 1891 |
| 1823 | PIGT | phosphatidylinositol glycan anchor biosynthesis class T | 51604 |
| 1824 | SRP14 | signal recognition particle 14 | 6727 |
| 1825 | NFS1 | NFS1, cysteine desulfurase | 9054 |
| 1826 | ARCN1 | archain 1 | 372 |
| 1827 | ARFIP2 | ADP ribosylation factor interacting protein 2 | 23647 |
| 1828 | RBM25 | RNA binding motif protein 25 | 58517 |
| 1829 | COPB1 | coatomer protein complex subunit beta 1 | 1315 |
| 1830 | KRT14 | keratin 14 | 3861 |
| 1831 | MSH6 | mutS homolog 6 | 2956 |
| 1832 | SCPEP1 | serine carboxypeptidase 1 | 59342 |
| 1833 | ATP1B1 | ATPase Na+/K+ transporting subunit beta 1 | 481 |
| 1834 | SNAP29 | synaptosome associated protein 29 | 9342 |
| 1835 | CD3EAP | CD3e molecule associated protein | 10849 |
| 1836 | KIF1C | kinesin family member 1C | 10749 |
| 1837 | PPIA | peptidylprolyl isomerase A | 5478 |
| 1838 | PREB | prolactin regulatory element binding | 10113 |
| 1839 | NIPSNAP2 | nipsnap homolog 2 | 2631 |
| 1840 | SHMT1 | serine hydroxymethyltransferase 1 | 6470 |
| 1841 | AP3B1 | adaptor related protein complex 3 subunit beta 1 | 8546 |
| 1842 | RPS15A | ribosomal protein S15a | 6210 |
| 1843 | CTSZ | cathepsin Z | 1522 |
| 1844 | DOCK6 | dedicator of cytokinesis 6 | 57572 |
| 1845 | CPT2 | carnitine palmitoyltransferase 2 | 1376 |
| 1846 | LDHB | lactate dehydrogenase B | 3945 |
| 1847 | GNG5 | G protein subunit gamma 5 | 2787 |
| 1848 | EIF4EBP1 | eukaryotic translation initiation factor 4E binding protein 1 | 1978 |
| 1849 | SUPT5H | SPT5 homolog, DSIF elongation factor subunit | 6829 |
| 1850 | DCPS | decapping enzyme, scavenger | 28960 |
| 1851 | MRPS18B | mitochondrial ribosomal protein S18B | 28973 |
| 1852 | TBL2 | transducin beta like 2 | 26608 |
| 1853 | PDCD4 | programmed cell death 4 | 27250 |
| 1854 | ATXN2L | ataxin 2 like | 11273 |
| 1855 | MIA3 | MIA SH3 domain ER export factor 3 | 375056 |
| 1856 | DAGLB | diacylglycerol lipase beta | 221955 |
| 1857 | NUP214 | nucleoporin 214 | 8021 |
| 1858 | ABHD14A-ACY1 | ABHD14A-ACY1 readthrough | 1.01E+08 |
| 1859 | PXN | paxillin | 5829 |
| 1860 | TXN2 | thioredoxin 2 | 25828 |
| 1861 | MARCKS | myristoylated alanine rich protein kinase C substrate | 4082 |
| 1862 | FLII | FLII, actin remodeling protein | 2314 |
| 1863 | TRIM32 | tripartite motif containing 32 | 22954 |
| 1864 | ARFIP1 | ADP ribosylation factor interacting protein 1 | 27236 |
| 1865 | ARG1 | arginase 1 | 383 |
| 1866 | HELZ2 | helicase with zinc finger 2 | 85441 |
| 1867 | OGFR | opioid growth factor receptor | 11054 |
| 1868 | PSMA1 | proteasome subunit alpha 1 | 5682 |
| 1869 | STX5 | syntaxin 5 | 6811 |
| 1870 | PEA15 | proliferation and apoptosis adaptor protein 15 | 8682 |
| 1871 | USP39 | ubiquitin specific peptidase 39 | 10713 |
| 1872 | HK1 | hexokinase 1 | 3098 |
| 1873 | RILPL1 | Rab interacting lysosomal protein like 1 | 353116 |
| 1874 | PSMA2 | proteasome subunit alpha 2 | 5683 |
| 1875 | CYB5A | cytochrome b5 type A | 1528 |
| 1876 | CCNDBP1 | cyclin D1 binding protein 1 | 23582 |
| 1877 | DNAJA2 | DnaJ heat shock protein family (Hsp40) member A2 | 10294 |
| 1878 | EXOC8 | exocyst complex component 8 | 149371 |
| 1879 | GFM1 | G elongation factor mitochondrial 1 | 85476 |
| 1880 | MANF | mesencephalic astrocyte derived neurotrophic factor | 7873 |
| 1881 | MRPL24 | mitochondrial ribosomal protein L24 | 79590 |
| 1882 | NUCB1 | nucleobindin 1 | 4924 |
| 1883 | BOP1 | BOP1 ribosomal biogenesis factor | 23246 |
| 1884 | NUDCD3 | NudC domain containing 3 | 23386 |
| 1885 | GDI2 | GDP dissociation inhibitor 2 | 2665 |
| 1886 | AFDN | afadin, adherens junction formation factor | 4301 |
| 1887 | HNRNPH2 | heterogeneous nuclear ribonucleoprotein H2 | 3188 |
| 1888 | TUBB3 | tubulin beta 3 class III | 10381 |
| 1889 | HDAC1 | histone deacetylase 1 | 3065 |
| 1890 | DHX15 | DEAH-box helicase 15 | 1665 |
| 1891 | AARS2 | alanyl-tRNA synthetase 2, mitochondrial | 57505 |
| 1892 | MOV10 | Mov10 RISC complex RNA helicase | 4343 |
| 1893 | TPMT | thiopurine S-methyltransferase | 7172 |
| 1894 | ERLIN2 | ER lipid raft associated 2 | 11160 |
| 1895 | SRRM1 | serine and arginine repetitive matrix 1 | 10250 |
| 1896 | UBXN7 | UBX domain protein 7 | 26043 |
| 1897 | SOX15 | SRY-box 15 | 6665 |
| 1898 | IFIH1 | interferon induced with helicase C domain 1 | 64135 |
| 1899 | KHSRP | KH-type splicing regulatory protein | 8570 |
| 1900 | RRAS2 | RAS related 2 | 22800 |
| 1901 | PFDN1 | prefoldin subunit 1 | 5201 |
| 1902 | COMMD3 | COMM domain containing 3 | 23412 |
| 1903 | PTGES2 | prostaglandin E synthase 2 | 80142 |
| 1904 | RAD23A | RAD23 homolog A, nucleotide excision repair protein | 5886 |
| 1905 | PSIP1 | PC4 and SFRS1 interacting protein 1 | 11168 |
| 1906 | BCAM | basal cell adhesion molecule (Lutheran blood group) | 4059 |
| 1907 | CYCS | cytochrome c, somatic | 54205 |
| 1908 | CALD1 | caldesmon 1 | 800 |
| 1909 | PRXL2A | peroxiredoxin like 2A | 84293 |
| 1910 | KRT5 | keratin 5 | 3852 |
| 1911 | NAP1L4 | nucleosome assembly protein 1 like 4 | 4676 |
| 1912 | HMGA2 | high mobility group AT-hook 2 | 8091 |
| 1913 | GSR | glutathione-disulfide reductase | 2936 |
| 1914 | HSDL2 | hydroxysteroid dehydrogenase like 2 | 84263 |
| 1915 | CUL4B | cullin 4B | 8450 |
| 1916 | SPTBN1 | spectrin beta, non-erythrocytic 1 | 6711 |
| 1917 | HBA1 | hemoglobin subunit alpha 1 | 3039 |
| 1918 | SLC25A13 | solute carrier family 25 member 13 | 10165 |
| 1919 | ESD | esterase D | 2098 |
| 1920 | SF3B4 | splicing factor 3b subunit 4 | 10262 |
| 1921 | SMS | spermine synthase | 6611 |
| 1922 | THOC6 | THO complex 6 | 79228 |
| 1923 | STXBP2 | syntaxin binding protein 2 | 6813 |
| 1924 | NXF1 | nuclear RNA export factor 1 | 10482 |
| 1925 | MOCS2 | molybdenum cofactor synthesis 2 | 4338 |
| 1926 | ACTR3 | actin related protein 3 | 10096 |
| 1927 | RPL28 | ribosomal protein L28 | 6158 |
| 1928 | TUFM | Tu translation elongation factor, mitochondrial | 7284 |
| 1929 | ARPP19 | cAMP regulated phosphoprotein 19 | 10776 |
| 1930 | GOLIM4 | golgi integral membrane protein 4 | 27333 |
| 1931 | DYNC1LI1 | dynein cytoplasmic 1 light intermediate chain 1 | 51143 |
| 1932 | SELENOF | selenoprotein F | 9403 |
| 1933 | AATF | apoptosis antagonizing transcription factor | 26574 |
| 1934 | PRDX6 | peroxiredoxin 6 | 9588 |
| 1935 | CTNNA1 | catenin alpha 1 | 1495 |
| 1936 | KRT18 | keratin 18 | 3875 |
| 1937 | SMARCA4 | SWI/SNF related, matrix associated, actin dependent regulator of chromatin, subfamily a, member 4 | 6597 |
| 1938 | CALCOCO2 | calcium binding and coiled-coil domain 2 | 10241 |
| 1939 | DNAJB11 | DnaJ heat shock protein family (Hsp40) member B11 | 51726 |
| 1940 | EMC3 | ER membrane protein complex subunit 3 | 55831 |
| 1941 | SF3B6 | splicing factor 3b subunit 6 | 51639 |
| 1942 | UBA2 | ubiquitin like modifier activating enzyme 2 | 10054 |
| 1943 | RBM17 | RNA binding motif protein 17 | 84991 |
| 1944 | RPL23A | ribosomal protein L23a | 6147 |
| 1945 | SET | SET nuclear proto-oncogene | 6418 |
| 1946 | GPRC5A | G protein-coupled receptor class C group 5 member A | 9052 |
| 1947 | EIF3M | eukaryotic translation initiation factor 3 subunit M | 10480 |
| 1948 | RPS16 | ribosomal protein S16 | 6217 |
| 1949 | MRPL46 | mitochondrial ribosomal protein L46 | 26589 |
| 1950 | C5orf51 | chromosome 5 open reading frame 51 | 285636 |
| 1951 | LSS | lanosterol synthase | 4047 |
| 1952 | RCL1 | RNA terminal phosphate cyclase like 1 | 10171 |
| 1953 | VCL | vinculin | 7414 |
| 1954 | GPKOW | G-patch domain and KOW motifs | 27238 |
| 1955 | CTNND1 | catenin delta 1 | 1500 |
| 1956 | HGS | hepatocyte growth factor-regulated tyrosine kinase substrate | 9146 |
| 1957 | SPTAN1 | spectrin alpha, non-erythrocytic 1 | 6709 |
| 1958 | NSUN2 | NOP2/Sun RNA methyltransferase family member 2 | 54888 |
| 1959 | PDHB | pyruvate dehydrogenase E1 beta subunit | 5162 |
| 1960 | MVP | major vault protein | 9961 |
| 1961 | IRF3 | interferon regulatory factor 3 | 3661 |
| 1962 | HNRNPUL1 | heterogeneous nuclear ribonucleoprotein U like 1 | 11100 |
| 1963 | TWF1 | twinfilin actin binding protein 1 | 5756 |
| 1964 | TXNDC9 | thioredoxin domain containing 9 | 10190 |
| 1965 | PRPF3 | pre-mRNA processing factor 3 | 9129 |
| 1966 | NPTN | neuroplastin | 27020 |
| 1967 | TCIRG1 | T cell immune regulator 1, ATPase H+ transporting V0 subunit a3 | 10312 |
| 1968 | RPRD1A | regulation of nuclear pre-mRNA domain containing 1A | 55197 |
| 1969 | POLDIP2 | DNA polymerase delta interacting protein 2 | 26073 |
| 1970 | DVL2 | dishevelled segment polarity protein 2 | 1856 |
| 1971 | GM2A | GM2 ganglioside activator | 2760 |
| 1972 | LRPAP1 | LDL receptor related protein associated protein 1 | 4043 |
| 1973 | EXOC5 | exocyst complex component 5 | 10640 |
| 1974 | CMAS | cytidine monophosphate N-acetylneuraminic acid synthetase | 55907 |
| 1975 | RAB21 | RAB21, member RAS oncogene family | 23011 |
| 1976 | DBN1 | drebrin 1 | 1627 |
| 1977 | SMNDC1 | survival motor neuron domain containing 1 | 10285 |
| 1978 | CACYBP | calcyclin binding protein | 27101 |
| 1979 | UBLCP1 | ubiquitin like domain containing CTD phosphatase 1 | 134510 |
| 1980 | UQCRH | ubiquinol-cytochrome c reductase hinge protein | 7388 |
| 1981 | AKR1C3 | aldo-keto reductase family 1 member C3 | 8644 |
| 1982 | CD47 | CD47 molecule | 961 |
| 1983 | RPL15 | ribosomal protein L15 | 6138 |
| 1984 | PPIL4 | peptidylprolyl isomerase like 4 | 85313 |
| 1985 | PSME2 | proteasome activator subunit 2 | 5721 |
| 1986 | DAP3 | death associated protein 3 | 7818 |
| 1987 | NUP93 | nucleoporin 93 | 9688 |
| 1988 | RALB | RAS like proto-oncogene B | 5899 |
| 1989 | ITGB1 | integrin subunit beta 1 | 3688 |
| 1990 | NACA | nascent polypeptide associated complex subunit alpha | 4666 |
| 1991 | IL36G | interleukin 36 gamma | 56300 |
| 1992 | RPS19 | ribosomal protein S19 | 6223 |
| 1993 | SHTN1 | shootin 1 | 57698 |
| 1994 | RPL7A | ribosomal protein L7a | 6130 |
| 1995 | RPS5 | ribosomal protein S5 | 6193 |
| 1996 | ETFB | electron transfer flavoprotein subunit beta | 2109 |
| 1997 | NOC2L | NOC2 like nucleolar associated transcriptional repressor | 26155 |
| 1998 | ATP13A1 | ATPase 13A1 | 57130 |
| 1999 | POLR2B | RNA polymerase II subunit B | 5431 |
| 2000 | KIF23 | kinesin family member 23 | 9493 |
| 2001 | NUP153 | nucleoporin 153 | 9972 |
| 2002 | RAB1B | RAB1B, member RAS oncogene family | 81876 |
| 2003 | PSME4 | proteasome activator subunit 4 | 23198 |
| 2004 | GSTZ1 | glutathione S-transferase zeta 1 | 2954 |
| 2005 | ABI1 | abl interactor 1 | 10006 |
| 2006 | PIH1D1 | PIH1 domain containing 1 | 55011 |
| 2007 | ARL2BP | ADP ribosylation factor like GTPase 2 binding protein | 23568 |
| 2008 | TMOD3 | tropomodulin 3 | 29766 |
| 2009 | HNRNPA3 | heterogeneous nuclear ribonucleoprotein A3 | 220988 |
| 2010 | PHPT1 | phosphohistidine phosphatase 1 | 29085 |
| 2011 | KMT2C | lysine methyltransferase 2C | 58508 |
| 2012 | THOP1 | thimet oligopeptidase 1 | 7064 |
| 2013 | SPTBN2 | spectrin beta, non-erythrocytic 2 | 6712 |
| 2014 | SRPRB | SRP receptor subunit beta | 58477 |
| 2015 | CBX1 | chromobox 1 | 10951 |
| 2016 | NUMB | NUMB, endocytic adaptor protein | 8650 |
| 2017 | UMPS | uridine monophosphate synthetase | 7372 |
| 2018 | PLCG1 | phospholipase C gamma 1 | 5335 |
| 2019 | GNAS | GNAS complex locus | 2778 |
| 2020 | FEN1 | flap structure-specific endonuclease 1 | 2237 |
| 2021 | YAP1 | Yes associated protein 1 | 10413 |
| 2022 | ABRAXAS2 | abraxas 2, BRISC complex subunit | 23172 |
| 2023 | OTUD7A | OTU deubiquitinase 7A | 161725 |
| 2024 | EHD2 | EH domain containing 2 | 30846 |
| 2025 | MDH2 | malate dehydrogenase 2 | 4191 |
| 2026 | FAM169A | family with sequence similarity 169 member A | 26049 |
| 2027 | CTR9 | CTR9 homolog, Paf1/RNA polymerase II complex component | 9646 |
| 2028 | PSMC2 | proteasome 26S subunit, ATPase 2 | 5701 |
| 2029 | ABCF2 | ATP binding cassette subfamily F member 2 | 10061 |
| 2030 | PDCD6 | programmed cell death 6 | 10016 |
| 2031 | TM9SF3 | transmembrane 9 superfamily member 3 | 56889 |
| 2032 | ECE1 | endothelin converting enzyme 1 | 1889 |
| 2033 | ASAH1 | N-acylsphingosine amidohydrolase 1 | 427 |
| 2034 | LEMD2 | LEM domain containing 2 | 221496 |
| 2035 | UBE2G1 | ubiquitin conjugating enzyme E2 G1 | 7326 |
| 2036 | HAX1 | HCLS1 associated protein X-1 | 10456 |
| 2037 | SRP54 | signal recognition particle 54 | 6729 |
| 2038 | DHCR7 | 7-dehydrocholesterol reductase | 1717 |
| 2039 | MSLN | mesothelin | 10232 |
| 2040 | TLN1 | talin 1 | 7094 |
| 2041 | MLEC | malectin | 9761 |
| 2042 | LZTFL1 | leucine zipper transcription factor like 1 | 54585 |
| 2043 | HRNR | hornerin | 388697 |
| 2044 | WDR18 | WD repeat domain 18 | 57418 |
| 2045 | SNX1 | sorting nexin 1 | 6642 |
| 2046 | STK39 | serine/threonine kinase 39 | 27347 |
| 2047 | RBX1 | ring-box 1 | 9978 |
| 2048 | PLCB3 | phospholipase C beta 3 | 5331 |
| 2049 | CSNK2B | casein kinase 2 beta | 1460 |
| 2050 | PLAU | plasminogen activator, urokinase | 5328 |
| 2051 | PSPC1 | paraspeckle component 1 | 55269 |
| 2052 | KHDRBS1 | KH RNA binding domain containing, signal transduction associated 1 | 10657 |
| 2053 | PDCL3 | phosducin like 3 | 79031 |
| 2054 | MYOF | myoferlin | 26509 |
| 2055 | SERPINB3 | serpin family B member 3 | 6317 |
| 2056 | DHRS7B | dehydrogenase/reductase 7B | 25979 |
| 2057 | EIF1 | eukaryotic translation initiation factor 1 | 10209 |
| 2058 | UFL1 | UFM1 specific ligase 1 | 23376 |
| 2059 | PDHA1 | pyruvate dehydrogenase E1 alpha 1 subunit | 5160 |
| 2060 | OSGEP | O-sialoglycoprotein endopeptidase | 55644 |
| 2061 | PFKL | phosphofructokinase, liver type | 5211 |
| 2062 | RPS20 | ribosomal protein S20 | 6224 |
| 2063 | FDFT1 | farnesyl-diphosphate farnesyltransferase 1 | 2222 |
| 2064 | ATP5PO | ATP synthase peripheral stalk subunit OSCP | 539 |
| 2065 | RDX | radixin | 5962 |
| 2066 | RBBP6 | RB binding protein 6, ubiquitin ligase | 5930 |
| 2067 | POLR1C | RNA polymerase I and III subunit C | 9533 |
| 2068 | RPS26 | ribosomal protein S26 | 6231 |
| 2069 | PSMD6 | proteasome 26S subunit, non-ATPase 6 | 9861 |
| 2070 | EIF3B | eukaryotic translation initiation factor 3 subunit B | 8662 |
| 2071 | PTGR1 | prostaglandin reductase 1 | 22949 |
| 2072 | HOMER3 | homer scaffold protein 3 | 9454 |
| 2073 | SLC44A2 | solute carrier family 44 member 2 | 57153 |
| 2074 | USP15 | ubiquitin specific peptidase 15 | 9958 |
| 2075 | CUL5 | cullin 5 | 8065 |
| 2076 | WASF2 | WAS protein family member 2 | 10163 |
| 2077 | SEC23A | Sec23 homolog A, coat complex II component | 10484 |
| 2078 | RPAP3 | RNA polymerase II associated protein 3 | 79657 |
| 2079 | LYST | lysosomal trafficking regulator | 1130 |
| 2080 | CSDE1 | cold shock domain containing E1 | 7812 |
| 2081 | B4GALT1 | beta-1,4-galactosyltransferase 1 | 2683 |
| 2082 | PPP1R7 | protein phosphatase 1 regulatory subunit 7 | 5510 |
| 2083 | RABEP2 | rabaptin, RAB GTPase binding effector protein 2 | 79874 |
| 2084 | RAB12 | RAB12, member RAS oncogene family | 201475 |
| 2085 | PCYT2 | phosphate cytidylyltransferase 2, ethanolamine | 5833 |
| 2086 | GANAB | glucosidase II alpha subunit | 23193 |
| 2087 | BAZ1B | bromodomain adjacent to zinc finger domain 1B | 9031 |
| 2088 | SLAIN2 | SLAIN motif family member 2 | 57606 |
| 2089 | ATAD1 | ATPase family, AAA domain containing 1 | 84896 |
| 2090 | PIGS | phosphatidylinositol glycan anchor biosynthesis class S | 94005 |
| 2091 | CHCHD3 | coiled-coil-helix-coiled-coil-helix domain containing 3 | 54927 |
| 2092 | MTREX | Mtr4 exosome RNA helicase | 23517 |
| 2093 | CCT7 | chaperonin containing TCP1 subunit 7 | 10574 |
| 2094 | KRT4 | keratin 4 | 3851 |
| 2095 | DGKA | diacylglycerol kinase alpha | 1606 |
| 2096 | CCAR2 | cell cycle and apoptosis regulator 2 | 57805 |
| 2097 | SMU1 | SMU1, DNA replication regulator and spliceosomal factor | 55234 |
| 2098 | EIF3A | eukaryotic translation initiation factor 3 subunit A | 8661 |
| 2099 | PRPF4 | pre-mRNA processing factor 4 | 9128 |
| 2100 | GUSB | glucuronidase beta | 2990 |
| 2101 | XRCC5 | X-ray repair cross complementing 5 | 7520 |
| 2102 | RABGAP1 | RAB GTPase activating protein 1 | 23637 |
| 2103 | RPN1 | ribophorin I | 6184 |
| 2104 | MYO1D | myosin ID | 4642 |
| 2105 | TMEM43 | transmembrane protein 43 | 79188 |
| 2106 | TMPO | thymopoietin | 7112 |
| 2107 | RTCA | RNA 3'-terminal phosphate cyclase | 8634 |
| 2108 | SNRPD1 | small nuclear ribonucleoprotein D1 polypeptide | 6632 |
| 2109 | CTTN | cortactin | 2017 |
| 2110 | TAOK3 | TAO kinase 3 | 51347 |
| 2111 | SLC25A12 | solute carrier family 25 member 12 | 8604 |
| 2112 | EPS15L1 | epidermal growth factor receptor pathway substrate 15 like 1 | 58513 |
| 2113 | RPS18 | ribosomal protein S18 | 6222 |
| 2114 | EMG1 | EMG1, N1-specific pseudouridine methyltransferase | 10436 |
| 2115 | SGPL1 | sphingosine-1-phosphate lyase 1 | 8879 |
| 2116 | VPS35 | VPS35, retromer complex component | 55737 |
| 2117 | PPP3CA | protein phosphatase 3 catalytic subunit alpha | 5530 |
| 2118 | CAPN1 | calpain 1 | 823 |
| 2119 | MAPK3 | mitogen-activated protein kinase 3 | 5595 |
| 2120 | KRT6A | keratin 6A | 3853 |
| 2121 | DECR1 | 2,4-dienoyl-CoA reductase 1 | 1666 |
| 2122 | TMED5 | transmembrane p24 trafficking protein 5 | 50999 |
| 2123 | CLINT1 | clathrin interactor 1 | 9685 |
| 2124 | ORMDL2 | ORMDL sphingolipid biosynthesis regulator 2 | 29095 |
| 2125 | RAI14 | retinoic acid induced 14 | 26064 |
| 2126 | CETN2 | centrin 2 | 1069 |
| 2127 | SF3B1 | splicing factor 3b subunit 1 | 23451 |
| 2128 | KCTD5 | potassium channel tetramerization domain containing 5 | 54442 |
| 2129 | EIF2B1 | eukaryotic translation initiation factor 2B subunit alpha | 1967 |
| 2130 | DIAPH1 | diaphanous related formin 1 | 1729 |
| 2131 | ITGAV | integrin subunit alpha V | 3685 |
| 2132 | RTN4 | reticulon 4 | 57142 |
| 2133 | RNF126 | ring finger protein 126 | 55658 |
| 2134 | ETF1 | eukaryotic translation termination factor 1 | 2107 |
| 2135 | CAPN2 | calpain 2 | 824 |
| 2136 | CPSF6 | cleavage and polyadenylation specific factor 6 | 11052 |
| 2137 | BCL2L13 | BCL2 like 13 | 23786 |
| 2138 | RPL30 | ribosomal protein L30 | 6156 |
| 2139 | VPS25 | vacuolar protein sorting 25 homolog | 84313 |
| 2140 | CTPS1 | CTP synthase 1 | 1503 |
| 2141 | UBE2L6 | ubiquitin conjugating enzyme E2 L6 | 9246 |
| 2142 | AGFG1 | ArfGAP with FG repeats 1 | 3267 |
| 2143 | CCDC43 | coiled-coil domain containing 43 | 124808 |
| 2144 | MYL12A | myosin light chain 12A | 10627 |
| 2145 | UFD1 | ubiquitin recognition factor in ER associated degradation 1 | 7353 |
| 2146 | IFITM3 | interferon induced transmembrane protein 3 | 10410 |
| 2147 | NCBP1 | nuclear cap binding protein subunit 1 | 4686 |
| 2148 | HSD17B10 | hydroxysteroid 17-beta dehydrogenase 10 | 3028 |
| 2149 | GCSH | glycine cleavage system protein H | 2653 |
| 2150 | TMEM126A | transmembrane protein 126A | 84233 |
| 2151 | PSMC1 | proteasome 26S subunit, ATPase 1 | 5700 |
| 2152 | HSP90AA1 | heat shock protein 90 alpha family class A member 1 | 3320 |
| 2153 | SNCA | synuclein alpha | 6622 |
| 2154 | PSMD3 | proteasome 26S subunit, non-ATPase 3 | 5709 |
| 2155 | NIP7 | NIP7, nucleolar pre-rRNA processing protein | 51388 |
| 2156 | SRSF4 | serine and arginine rich splicing factor 4 | 6429 |
| 2157 | CD63 | CD63 molecule | 967 |
| 2158 | OGA | O-GlcNAcase | 10724 |
| 2159 | ACSF2 | acyl-CoA synthetase family member 2 | 80221 |
| 2160 | FAM162A | family with sequence similarity 162 member A | 26355 |
| 2161 | CS | citrate synthase | 1431 |
| 2162 | GINS4 | GINS complex subunit 4 | 84296 |
| 2163 | AP3M1 | adaptor related protein complex 3 subunit mu 1 | 26985 |
| 2164 | PLOD3 | procollagen-lysine,2-oxoglutarate 5-dioxygenase 3 | 8985 |
| 2165 | NSDHL | NAD(P) dependent steroid dehydrogenase-like | 50814 |
| 2166 | TRIP13 | thyroid hormone receptor interactor 13 | 9319 |
| 2167 | ACTR1A | actin related protein 1A | 10121 |
| 2168 | DCTN1 | dynactin subunit 1 | 1639 |
| 2169 | PKP3 | plakophilin 3 | 11187 |
| 2170 | DOCK7 | dedicator of cytokinesis 7 | 85440 |
| 2171 | ALDOC | aldolase, fructose-bisphosphate C | 230 |
| 2172 | RFC3 | replication factor C subunit 3 | 5983 |
| 2173 | U2AF2 | U2 small nuclear RNA auxiliary factor 2 | 11338 |
| 2174 | SUPT6H | SPT6 homolog, histone chaperone | 6830 |
| 2175 | CALML3 | calmodulin like 3 | 810 |
| 2176 | SLC20A2 | solute carrier family 20 member 2 | 6575 |
| 2177 | TBCE | tubulin folding cofactor E | 6905 |
| 2178 | POP1 | POP1 homolog, ribonuclease P/MRP subunit | 10940 |
| 2179 | ME1 | malic enzyme 1 | 4199 |
| 2180 | PPP1R18 | protein phosphatase 1 regulatory subunit 18 | 170954 |
| 2181 | DMAC2 | distal membrane arm assembly complex 2 | 55101 |
| 2182 | FARSA | phenylalanyl-tRNA synthetase subunit alpha | 2193 |
| 2183 | AFAP1L2 | actin filament associated protein 1 like 2 | 84632 |
| 2184 | MCM2 | minichromosome maintenance complex component 2 | 4171 |
| 2185 | SF3B3 | splicing factor 3b subunit 3 | 23450 |
| 2186 | TRADD | TNFRSF1A associated via death domain | 8717 |
| 2187 | TSR1 | TSR1, ribosome maturation factor | 55720 |
| 2188 | RPS6KA1 | ribosomal protein S6 kinase A1 | 6195 |
| 2189 | DNAJB1 | DnaJ heat shock protein family (Hsp40) member B1 | 3337 |
| 2190 | PKN2 | protein kinase N2 | 5586 |
| 2191 | FLOT1 | flotillin 1 | 10211 |
| 2192 | ENAH | ENAH, actin regulator | 55740 |
| 2193 | HNRNPDL | heterogeneous nuclear ribonucleoprotein D like | 9987 |
| 2194 | EFNB1 | ephrin B1 | 1947 |
| 2195 | SF3A3 | splicing factor 3a subunit 3 | 10946 |
| 2196 | UFSP2 | UFM1 specific peptidase 2 | 55325 |
| 2197 | RPL11 | ribosomal protein L11 | 6135 |
| 2198 | KPNA4 | karyopherin subunit alpha 4 | 3840 |
| 2199 | TAF15 | TATA-box binding protein associated factor 15 | 8148 |
| 2200 | EFL1 | elongation factor like GTPase 1 | 79631 |
| 2201 | UROD | uroporphyrinogen decarboxylase | 7389 |
| 2202 | EXOC1 | exocyst complex component 1 | 55763 |
| 2203 | RAB14 | RAB14, member RAS oncogene family | 51552 |
| 2204 | LLGL1 | LLGL scribble cell polarity complex component 1 | 3996 |
| 2205 | PTBP3 | polypyrimidine tract binding protein 3 | 9991 |
| 2206 | TMEM192 | transmembrane protein 192 | 201931 |
| 2207 | CIRBP | cold inducible RNA binding protein | 1153 |
| 2208 | ZNF207 | zinc finger protein 207 | 7756 |
| 2209 | GINS3 | GINS complex subunit 3 | 64785 |
| 2210 | KIF4A | kinesin family member 4A | 24137 |
| 2211 | RAB10 | RAB10, member RAS oncogene family | 10890 |
| 2212 | GALE | UDP-galactose-4-epimerase | 2582 |
| 2213 | SLC25A3 | solute carrier family 25 member 3 | 5250 |
| 2214 | SERINC1 | serine incorporator 1 | 57515 |
| 2215 | ATP2A2 | ATPase sarcoplasmic/endoplasmic reticulum Ca2+ transporting 2 | 488 |
| 2216 | NAGK | N-acetylglucosamine kinase | 55577 |
| 2217 | CSRP2 | cysteine and glycine rich protein 2 | 1466 |
| 2218 | KRT17 | keratin 17 | 3872 |
| 2219 | LCLAT1 | lysocardiolipin acyltransferase 1 | 253558 |
| 2220 | SPINT1 | serine peptidase inhibitor, Kunitz type 1 | 6692 |
| 2221 | NME1-NME2 | NME1-NME2 readthrough | 654364 |
| 2222 | IMPA2 | inositol monophosphatase 2 | 3613 |
| 2223 | VDAC3 | voltage dependent anion channel 3 | 7419 |
| 2224 | PSMD8 | proteasome 26S subunit, non-ATPase 8 | 5714 |
| 2225 | LMNA | lamin A/C | 4000 |
| 2226 | SNCG | synuclein gamma | 6623 |
| 2227 | LAMA3 | laminin subunit alpha 3 | 3909 |
| 2228 | PDIA3 | protein disulfide isomerase family A member 3 | 2923 |
| 2229 | NPM1 | nucleophosmin 1 | 4869 |
| 2230 | PRPS1 | phosphoribosyl pyrophosphate synthetase 1 | 5631 |
| 2231 | RETSAT | retinol saturase | 54884 |
| 2232 | GOLGB1 | golgin B1 | 2804 |
| 2233 | NUP88 | nucleoporin 88 | 4927 |
| 2234 | HNRNPC | heterogeneous nuclear ribonucleoprotein C (C1/C2) | 3183 |
| 2235 | KRT8 | keratin 8 | 3856 |
| 2236 | RPS14 | ribosomal protein S14 | 6208 |
| 2237 | POLE3 | DNA polymerase epsilon 3, accessory subunit | 54107 |
| 2238 | NEDD1 | neural precursor cell expressed, developmentally down-regulated 1 | 121441 |
| 2239 | CRIP2 | cysteine rich protein 2 | 1397 |
| 2240 | LDLR | low density lipoprotein receptor | 3949 |
| 2241 | BCAT2 | branched chain amino acid transaminase 2 | 587 |
| 2242 | GLUD1 | glutamate dehydrogenase 1 | 2746 |
| 2243 | CPOX | coproporphyrinogen oxidase | 1371 |
| 2244 | LAMTOR5 | late endosomal/lysosomal adaptor, MAPK and MTOR activator 5 | 10542 |
| 2245 | APEX1 | apurinic/apyrimidinic endodeoxyribonuclease 1 | 328 |
| 2246 | PLEK2 | pleckstrin 2 | 26499 |
| 2247 | ILF2 | interleukin enhancer binding factor 2 | 3608 |
| 2248 | CDH1 | cadherin 1 | 999 |
| 2249 | SUPT16H | SPT16 homolog, facilitates chromatin remodeling subunit | 11198 |
| 2250 | SERPINB6 | serpin family B member 6 | 5269 |
| 2251 | PPM1A | protein phosphatase, Mg2+/Mn2+ dependent 1A | 5494 |
| 2252 | PYCR2 | pyrroline-5-carboxylate reductase 2 | 29920 |
| 2253 | ATP2B4 | ATPase plasma membrane Ca2+ transporting 4 | 493 |
| 2254 | TBC1D15 | TBC1 domain family member 15 | 64786 |
| 2255 | EPPK1 | epiplakin 1 | 83481 |
| 2256 | CCDC58 | coiled-coil domain containing 58 | 131076 |
| 2257 | UAP1L1 | UDP-N-acetylglucosamine pyrophosphorylase 1 like 1 | 91373 |
| 2258 | RUVBL1 | RuvB like AAA ATPase 1 | 8607 |
| 2259 | CKAP5 | cytoskeleton associated protein 5 | 9793 |
| 2260 | CWC15 | CWC15 spliceosome associated protein homolog | 51503 |
| 2261 | FNBP1L | formin binding protein 1 like | 54874 |
| 2262 | HNRNPF | heterogeneous nuclear ribonucleoprotein F | 3185 |
| 2263 | ABCD3 | ATP binding cassette subfamily D member 3 | 5825 |
| 2264 | NDUFA9 | NADH:ubiquinone oxidoreductase subunit A9 | 4704 |
| 2265 | AK4 | adenylate kinase 4 | 205 |
| 2266 | CARHSP1 | calcium regulated heat stable protein 1 | 23589 |
| 2267 | ERC1 | ELKS/RAB6-interacting/CAST family member 1 | 23085 |
| 2268 | SCRN1 | secernin 1 | 9805 |
| 2269 | PRMT3 | protein arginine methyltransferase 3 | 10196 |
| 2270 | SLK | STE20 like kinase | 9748 |
| 2271 | RPL10A | ribosomal protein L10a | 4736 |
| 2272 | PNKP | polynucleotide kinase 3'-phosphatase | 11284 |
| 2273 | CUL2 | cullin 2 | 8453 |
| 2274 | CDKN2AIPNL | CDKN2A interacting protein N-terminal like | 91368 |
| 2275 | YY1 | YY1 transcription factor | 7528 |
| 2276 | RTL9 | retrotransposon Gag like 9 | 57529 |
| 2277 | ECHS1 | enoyl-CoA hydratase, short chain 1 | 1892 |
| 2278 | ARHGEF7 | Rho guanine nucleotide exchange factor 7 | 8874 |
| 2279 | ALG5 | ALG5, dolichyl-phosphate beta-glucosyltransferase | 29880 |
| 2280 | PAICS | phosphoribosylaminoimidazole carboxylase and phosphoribosylaminoimidazolesuccinocarboxamide synthase | 10606 |
| 2281 | XRCC1 | X-ray repair cross complementing 1 | 7515 |
| 2282 | TRIP6 | thyroid hormone receptor interactor 6 | 7205 |
| 2283 | TNC | tenascin C | 3371 |
| 2284 | MRPL30 | mitochondrial ribosomal protein L30 | 51263 |
| 2285 | RPS3A | ribosomal protein S3A | 6189 |
| 2286 | SPCS1 | signal peptidase complex subunit 1 | 28972 |
| 2287 | EIF3K | eukaryotic translation initiation factor 3 subunit K | 27335 |
| 2288 | ACOT13 | acyl-CoA thioesterase 13 | 55856 |
| 2289 | NELFA | negative elongation factor complex member A | 7469 |
| 2290 | GAA | glucosidase alpha, acid | 2548 |
| 2291 | ACTL6A | actin like 6A | 86 |
| 2292 | RBBP9 | RB binding protein 9, serine hydrolase | 10741 |
| 2293 | UBE2R2 | ubiquitin conjugating enzyme E2 R2 | 54926 |
| 2294 | VSNL1 | visinin like 1 | 7447 |
| 2295 | TECR | trans-2,3-enoyl-CoA reductase | 9524 |
| 2296 | HIP1R | huntingtin interacting protein 1 related | 9026 |
| 2297 | LAMC1 | laminin subunit gamma 1 | 3915 |
| 2298 | SLC9A3R2 | SLC9A3 regulator 2 | 9351 |
| 2299 | NEK9 | NIMA related kinase 9 | 91754 |
| 2300 | CNP | 2',3'-cyclic nucleotide 3' phosphodiesterase | 1267 |
| 2301 | PPL | periplakin | 5493 |
| 2302 | PLRG1 | pleiotropic regulator 1 | 5356 |
| 2303 | ADAM9 | ADAM metallopeptidase domain 9 | 8754 |
| 2304 | SDC4 | syndecan 4 | 6385 |
| 2305 | MAGT1 | magnesium transporter 1 | 84061 |
| 2306 | PIP4K2C | phosphatidylinositol-5-phosphate 4-kinase type 2 gamma | 79837 |
| 2307 | CLPTM1 | CLPTM1, transmembrane protein | 1209 |
| 2308 | RUVBL2 | RuvB like AAA ATPase 2 | 10856 |
| 2309 | ATOX1 | antioxidant 1 copper chaperone | 475 |
| 2310 | RPL23 | ribosomal protein L23 | 9349 |
| 2311 | RAB6A | RAB6A, member RAS oncogene family | 5870 |
| 2312 | MTAP | methylthioadenosine phosphorylase | 4507 |
| 2313 | BANF1 | barrier to autointegration factor 1 | 8815 |
| 2314 | SH3BGRL | SH3 domain binding glutamate rich protein like | 6451 |
| 2315 | ATP6V1F | ATPase H+ transporting V1 subunit F | 9296 |
| 2316 | ELAVL1 | ELAV like RNA binding protein 1 | 1994 |
| 2317 | DPYSL2 | dihydropyrimidinase like 2 | 1808 |
| 2318 | MAP2K3 | mitogen-activated protein kinase kinase 3 | 5606 |
| 2319 | PRPSAP1 | phosphoribosyl pyrophosphate synthetase associated protein 1 | 5635 |
| 2320 | PAFAH1B1 | platelet activating factor acetylhydrolase 1b regulatory subunit 1 | 5048 |
| 2321 | LASP1 | LIM and SH3 protein 1 | 3927 |
| 2322 | PSMC3 | proteasome 26S subunit, ATPase 3 | 5702 |
| 2323 | FKBP9 | FKBP prolyl isomerase 9 | 11328 |
| 2324 | GTF2F2 | general transcription factor IIF subunit 2 | 2963 |
| 2325 | GOT2 | glutamic-oxaloacetic transaminase 2 | 2806 |
| 2326 | SIGMAR1 | sigma non-opioid intracellular receptor 1 | 10280 |
| 2327 | ILF3 | interleukin enhancer binding factor 3 | 3609 |
| 2328 | ASNS | asparagine synthetase (glutamine-hydrolyzing) | 440 |
| 2329 | ZFR | zinc finger RNA binding protein | 51663 |
| 2330 | FLOT2 | flotillin 2 | 2319 |
| 2331 | ALDH7A1 | aldehyde dehydrogenase 7 family member A1 | 501 |
| 2332 | TIPRL | TOR signaling pathway regulator | 261726 |
| 2333 | YTHDF3 | YTH N6-methyladenosine RNA binding protein 3 | 253943 |
| 2334 | TBL1XR1 | transducin beta like 1 X-linked receptor 1 | 79718 |
| 2335 | CAT | catalase | 847 |
| 2336 | MRTO4 | MRT4 homolog, ribosome maturation factor | 51154 |
| 2337 | ARFGAP2 | ADP ribosylation factor GTPase activating protein 2 | 84364 |
| 2338 | ROCK1 | Rho associated coiled-coil containing protein kinase 1 | 6093 |
| 2339 | RWDD1 | RWD domain containing 1 | 51389 |
| 2340 | NUDT2 | nudix hydrolase 2 | 318 |
| 2341 | C1QBP | complement C1q binding protein | 708 |
| 2342 | SFXN1 | sideroflexin 1 | 94081 |
| 2343 | LAP3 | leucine aminopeptidase 3 | 51056 |
| 2344 | PGAM5 | PGAM family member 5, mitochondrial serine/threonine protein phosphatase | 192111 |
| 2345 | ABCF1 | ATP binding cassette subfamily F member 1 | 23 |
| 2346 | AUP1 | AUP1, lipid droplet regulating VLDL assembly factor | 550 |
| 2347 | CSNK2A1 | casein kinase 2 alpha 1 | 1457 |
| 2348 | SRRM2 | serine/arginine repetitive matrix 2 | 23524 |
| 2349 | DCK | deoxycytidine kinase | 1633 |
| 2350 | DOCK9 | dedicator of cytokinesis 9 | 23348 |
| 2351 | SQLE | squalene epoxidase | 6713 |
| 2352 | SHC1 | SHC adaptor protein 1 | 6464 |
| 2353 | CREB1 | cAMP responsive element binding protein 1 | 1385 |
| 2354 | LETM1 | leucine zipper and EF-hand containing transmembrane protein 1 | 3954 |
| 2355 | GART | phosphoribosylglycinamide formyltransferase, phosphoribosylglycinamide synthetase, phosphoribosylaminoimidazole synthetase | 2618 |
| 2356 | SGTA | small glutamine rich tetratricopeptide repeat containing alpha | 6449 |
| 2357 | EIF3I | eukaryotic translation initiation factor 3 subunit I | 8668 |
| 2358 | NDUFB10 | NADH:ubiquinone oxidoreductase subunit B10 | 4716 |
| 2359 | HNRNPD | heterogeneous nuclear ribonucleoprotein D | 3184 |
| 2360 | YLPM1 | YLP motif containing 1 | 56252 |
| 2361 | GSK3B | glycogen synthase kinase 3 beta | 2932 |
| 2362 | RFC2 | replication factor C subunit 2 | 5982 |
| 2363 | RPS27A | ribosomal protein S27a | 6233 |
| 2364 | ARPC2 | actin related protein 2/3 complex subunit 2 | 10109 |
| 2365 | LNPK | lunapark, ER junction formation factor | 80856 |
| 2366 | ALCAM | activated leukocyte cell adhesion molecule | 214 |
| 2367 | CCDC124 | coiled-coil domain containing 124 | 115098 |
| 2368 | IKBKG | inhibitor of nuclear factor kappa B kinase subunit gamma | 8517 |
| 2369 | TMED2 | transmembrane p24 trafficking protein 2 | 10959 |
| 2370 | NCBP2 | nuclear cap binding protein subunit 2 | 22916 |
| 2371 | WBP11 | WW domain binding protein 11 | 51729 |
| 2372 | NDUFS7 | NADH:ubiquinone oxidoreductase core subunit S7 | 374291 |
| 2373 | DHX38 | DEAH-box helicase 38 | 9785 |
| 2374 | MAP4 | microtubule associated protein 4 | 4134 |
| 2375 | ITGA3 | integrin subunit alpha 3 | 3675 |
| 2376 | KRT16 | keratin 16 | 3868 |
| 2377 | ARF4 | ADP ribosylation factor 4 | 378 |
| 2378 | SLC7A5 | solute carrier family 7 member 5 | 8140 |
| 2379 | SSH3 | slingshot protein phosphatase 3 | 54961 |
| 2380 | PC | pyruvate carboxylase | 5091 |
| 2381 | SWAP70 | switching B cell complex subunit SWAP70 | 23075 |
| 2382 | LIPA | lipase A, lysosomal acid type | 3988 |
| 2383 | EEF2 | eukaryotic translation elongation factor 2 | 1938 |
| 2384 | EBP | EBP, cholestenol delta-isomerase | 10682 |
| 2385 | CAPZA2 | capping actin protein of muscle Z-line subunit alpha 2 | 830 |
| 2386 | ZMPSTE24 | zinc metallopeptidase STE24 | 10269 |
| 2387 | UCHL5 | ubiquitin C-terminal hydrolase L5 | 51377 |
| 2388 | RAB5B | RAB5B, member RAS oncogene family | 5869 |
| 2389 | ACADVL | acyl-CoA dehydrogenase very long chain | 37 |
| 2390 | FAM83G | family with sequence similarity 83 member G | 644815 |
| 2391 | COPB2 | coatomer protein complex subunit beta 2 | 9276 |
| 2392 | ACAT1 | acetyl-CoA acetyltransferase 1 | 38 |
| 2393 | ARFGEF2 | ADP ribosylation factor guanine nucleotide exchange factor 2 | 10564 |
| 2394 | RIOX1 | ribosomal oxygenase 1 | 79697 |
| 2395 | GNAQ | G protein subunit alpha q | 2776 |
| 2396 | TACC3 | transforming acidic coiled-coil containing protein 3 | 10460 |
| 2397 | MAIP1 | matrix AAA peptidase interacting protein 1 | 79568 |
| 2398 | RPL4 | ribosomal protein L4 | 6124 |
| 2399 | CBL | Cbl proto-oncogene | 867 |
| 2400 | RPL13 | ribosomal protein L13 | 6137 |
| 2401 | PSMD4 | proteasome 26S subunit, non-ATPase 4 | 5710 |
| 2402 | COPS7A | COP9 signalosome subunit 7A | 50813 |
| 2403 | CHMP2A | charged multivesicular body protein 2A | 27243 |
| 2404 | RAB31 | RAB31, member RAS oncogene family | 11031 |
| 2405 | MRE11 | MRE11 homolog, double strand break repair nuclease | 4361 |
| 2406 | AP1G2 | adaptor related protein complex 1 subunit gamma 2 | 8906 |
| 2407 | FAM177A1 | family with sequence similarity 177 member A1 | 283635 |
| 2408 | PACSIN2 | protein kinase C and casein kinase substrate in neurons 2 | 11252 |
| 2409 | PAPSS1 | 3'-phosphoadenosine 5'-phosphosulfate synthase 1 | 9061 |
| 2410 | OAS3 | 2'-5'-oligoadenylate synthetase 3 | 4940 |
| 2411 | ARF1 | ADP ribosylation factor 1 | 375 |
| 2412 | MAVS | mitochondrial antiviral signaling protein | 57506 |
| 2413 | DDX3X | DEAD-box helicase 3 X-linked | 1654 |
| 2414 | ACADM | acyl-CoA dehydrogenase medium chain | 34 |
| 2415 | TACSTD2 | tumor associated calcium signal transducer 2 | 4070 |
| 2416 | CKMT1A | creatine kinase, mitochondrial 1A | 548596 |
| 2417 | PMVK | phosphomevalonate kinase | 10654 |
| 2418 | PSMD1 | proteasome 26S subunit, non-ATPase 1 | 5707 |
| 2419 | ESRP1 | epithelial splicing regulatory protein 1 | 54845 |
| 2420 | RBM14 | RNA binding motif protein 14 | 10432 |
| 2421 | RPS10 | ribosomal protein S10 | 6204 |
| 2422 | GALNT3 | polypeptide N-acetylgalactosaminyltransferase 3 | 2591 |
| 2423 | GPS1 | G protein pathway suppressor 1 | 2873 |
| 2424 | PEBP1 | phosphatidylethanolamine binding protein 1 | 5037 |
| 2425 | PRKAB1 | protein kinase AMP-activated non-catalytic subunit beta 1 | 5564 |
| 2426 | KRT3 | keratin 3 | 3850 |
| 2427 | SLIRP | SRA stem-loop interacting RNA binding protein | 81892 |
| 2428 | RPL22 | ribosomal protein L22 | 6146 |
| 2429 | MAT2B | methionine adenosyltransferase 2B | 27430 |
| 2430 | ALDH4A1 | aldehyde dehydrogenase 4 family member A1 | 8659 |
| 2431 | RHOG | ras homolog family member G | 391 |
| 2432 | ATP2B1 | ATPase plasma membrane Ca2+ transporting 1 | 490 |
| 2433 | LRRFIP2 | LRR binding FLII interacting protein 2 | 9209 |
| 2434 | ACSS2 | acyl-CoA synthetase short chain family member 2 | 55902 |
| 2435 | RNPEP | arginyl aminopeptidase | 6051 |
| 2436 | LACTB2 | lactamase beta 2 | 51110 |
| 2437 | HMGCS1 | 3-hydroxy-3-methylglutaryl-CoA synthase 1 | 3157 |
| 2438 | CNOT1 | CCR4-NOT transcription complex subunit 1 | 23019 |
| 2439 | ALDH1B1 | aldehyde dehydrogenase 1 family member B1 | 219 |
| 2440 | SUCLA2 | succinate-CoA ligase ADP-forming beta subunit | 8803 |
| 2441 | PPWD1 | peptidylprolyl isomerase domain and WD repeat containing 1 | 23398 |
| 2442 | OXCT1 | 3-oxoacid CoA-transferase 1 | 5019 |
| 2443 | PAK2 | p21 (RAC1) activated kinase 2 | 5062 |
| 2444 | ADAM17 | ADAM metallopeptidase domain 17 | 6868 |
| 2445 | NDUFS4 | NADH:ubiquinone oxidoreductase subunit S4 | 4724 |
| 2446 | LPCAT1 | lysophosphatidylcholine acyltransferase 1 | 79888 |
| 2447 | ARFGAP1 | ADP ribosylation factor GTPase activating protein 1 | 55738 |
| 2448 | SF3B5 | splicing factor 3b subunit 5 | 83443 |
| 2449 | PIP | prolactin induced protein | 5304 |
| 2450 | EEF1A1 | eukaryotic translation elongation factor 1 alpha 1 | 1915 |
| 2451 | DPM1 | dolichyl-phosphate mannosyltransferase subunit 1, catalytic | 8813 |
| 2452 | CDK1 | cyclin dependent kinase 1 | 983 |
| 2453 | CISD2 | CDGSH iron sulfur domain 2 | 493856 |
| 2454 | RIPK1 | receptor interacting serine/threonine kinase 1 | 8737 |
| 2455 | NRBP1 | nuclear receptor binding protein 1 | 29959 |
| 2456 | HNRNPA1 | heterogeneous nuclear ribonucleoprotein A1 | 3178 |
| 2457 | RBBP7 | RB binding protein 7, chromatin remodeling factor | 5931 |
| 2458 | STRN | striatin | 6801 |
| 2459 | CPNE1 | copine 1 | 8904 |
| 2460 | TMX2 | thioredoxin related transmembrane protein 2 | 51075 |
| 2461 | CAB39 | calcium binding protein 39 | 51719 |
| 2462 | RAB18 | RAB18, member RAS oncogene family | 22931 |
| 2463 | CPNE3 | copine 3 | 8895 |
| 2464 | METAP2 | methionyl aminopeptidase 2 | 10988 |
| 2465 | GPX8 | glutathione peroxidase 8 (putative) | 493869 |
| 2466 | RTF1 | RTF1 homolog, Paf1/RNA polymerase II complex component | 23168 |
| 2467 | PCBP1 | poly(rC) binding protein 1 | 5093 |
| 2468 | CIAO1 | cytosolic iron-sulfur assembly component 1 | 9391 |
| 2469 | ATP5F1D | ATP synthase F1 subunit delta | 513 |
| 2470 | CNN2 | calponin 2 | 1265 |
| 2471 | IMPA1 | inositol monophosphatase 1 | 3612 |
| 2472 | GGPS1 | geranylgeranyl diphosphate synthase 1 | 9453 |
| 2473 | IPO7 | importin 7 | 10527 |
| 2474 | NELFB | negative elongation factor complex member B | 25920 |
| 2475 | RPS25 | ribosomal protein S25 | 6230 |
| 2476 | NAMPT | nicotinamide phosphoribosyltransferase | 10135 |
| 2477 | S100A14 | S100 calcium binding protein A14 | 57402 |
| 2478 | MAPKAPK3 | mitogen-activated protein kinase-activated protein kinase 3 | 7867 |
| 2479 | SCARB1 | scavenger receptor class B member 1 | 949 |
| 2480 | TMX3 | thioredoxin related transmembrane protein 3 | 54495 |
| 2481 | RPL13A | ribosomal protein L13a | 23521 |
| 2482 | HPCAL1 | hippocalcin like 1 | 3241 |
| 2483 | APLP2 | amyloid beta precursor like protein 2 | 334 |
| 2484 | KIAA1217 | KIAA1217 | 56243 |
| 2485 | LIN7C | lin-7 homolog C, crumbs cell polarity complex component | 55327 |
| 2486 | HMGB3 | high mobility group box 3 | 3149 |
| 2487 | DDRGK1 | DDRGK domain containing 1 | 65992 |
| 2488 | EIF3G | eukaryotic translation initiation factor 3 subunit G | 8666 |
| 2489 | PPP5C | protein phosphatase 5 catalytic subunit | 5536 |
| 2490 | HCFC1 | host cell factor C1 | 3054 |
| 2491 | RABGEF1 | RAB guanine nucleotide exchange factor 1 | 27342 |
| 2492 | CD2AP | CD2 associated protein | 23607 |
| 2493 | CIAPIN1 | cytokine induced apoptosis inhibitor 1 | 57019 |
| 2494 | RAB35 | RAB35, member RAS oncogene family | 11021 |
| 2495 | SRPK1 | SRSF protein kinase 1 | 6732 |
| 2496 | KRT73 | keratin 73 | 319101 |
| 2497 | P4HA2 | prolyl 4-hydroxylase subunit alpha 2 | 8974 |
| 2498 | MFAP1 | microfibril associated protein 1 | 4236 |
| 2499 | RBM22 | RNA binding motif protein 22 | 55696 |
| 2500 | MYO6 | myosin VI | 4646 |
| 2501 | ZGPAT | zinc finger CCCH-type and G-patch domain containing | 84619 |
| 2502 | UBE2I | ubiquitin conjugating enzyme E2 I | 7329 |
| 2503 | RAB43 | RAB43, member RAS oncogene family | 339122 |
| 2504 | NOLC1 | nucleolar and coiled-body phosphoprotein 1 | 9221 |
| 2505 | PTRH2 | peptidyl-tRNA hydrolase 2 | 51651 |
| 2506 | POU2F1 | POU class 2 homeobox 1 | 5451 |
| 2507 | NDUFS6 | NADH:ubiquinone oxidoreductase subunit S6 | 4726 |
| 2508 | TMED9 | transmembrane p24 trafficking protein 9 | 54732 |
| 2509 | BOLA1 | bolA family member 1 | 51027 |
| 2510 | PPP1R13L | protein phosphatase 1 regulatory subunit 13 like | 10848 |
| 2511 | UBA5 | ubiquitin like modifier activating enzyme 5 | 79876 |
| 2512 | SBSN | suprabasin | 374897 |
| 2513 | GEMIN4 | gem nuclear organelle associated protein 4 | 50628 |
| 2514 | RPS6KA3 | ribosomal protein S6 kinase A3 | 6197 |
| 2515 | ZRANB2 | zinc finger RANBP2-type containing 2 | 9406 |
| 2516 | PPIL1 | peptidylprolyl isomerase like 1 | 51645 |
| 2517 | DEGS1 | delta 4-desaturase, sphingolipid 1 | 8560 |
| 2518 | SLC3A2 | solute carrier family 3 member 2 | 6520 |
| 2519 | TUBA1C | tubulin alpha 1c | 84790 |
| 2520 | MCU | mitochondrial calcium uniporter | 90550 |
| 2521 | FUS | FUS RNA binding protein | 2521 |
| 2522 | UFM1 | ubiquitin fold modifier 1 | 51569 |
| 2523 | DDX6 | DEAD-box helicase 6 | 1656 |
| 2524 | NMNAT1 | nicotinamide nucleotide adenylyltransferase 1 | 64802 |
| 2525 | MRPS7 | mitochondrial ribosomal protein S7 | 51081 |
| 2526 | DNAJC10 | DnaJ heat shock protein family (Hsp40) member C10 | 54431 |
| 2527 | PDS5B | PDS5 cohesin associated factor B | 23047 |
| 2528 | STXBP3 | syntaxin binding protein 3 | 6814 |
| 2529 | NUP35 | nucleoporin 35 | 129401 |
| 2530 | TUBB | tubulin beta class I | 203068 |
| 2531 | PEX19 | peroxisomal biogenesis factor 19 | 5824 |
| 2532 | RPS12 | ribosomal protein S12 | 6206 |
| 2533 | ATP6V1H | ATPase H+ transporting V1 subunit H | 51606 |
| 2534 | RPL9 | ribosomal protein L9 | 6133 |
| 2535 | NCL | nucleolin | 4691 |
| 2536 | LPP | LIM domain containing preferred translocation partner in lipoma | 4026 |
| 2537 | PSMA7 | proteasome subunit alpha 7 | 5688 |
| 2538 | ZNF638 | zinc finger protein 638 | 27332 |
| 2539 | WTAP | WT1 associated protein | 9589 |
| 2540 | SLC39A10 | solute carrier family 39 member 10 | 57181 |
| 2541 | TIMM44 | translocase of inner mitochondrial membrane 44 | 10469 |
| 2542 | VTA1 | vesicle trafficking 1 | 51534 |
| 2543 | NIPSNAP3A | nipsnap homolog 3A | 25934 |
| 2544 | BCCIP | BRCA2 and CDKN1A interacting protein | 56647 |
| 2545 | PGP | phosphoglycolate phosphatase | 283871 |
| 2546 | TNPO1 | transportin 1 | 3842 |
| 2547 | STX18 | syntaxin 18 | 53407 |
| 2548 | UBA3 | ubiquitin like modifier activating enzyme 3 | 9039 |
| 2549 | GYS1 | glycogen synthase 1 | 2997 |
| 2550 | IGKC | immunoglobulin kappa constant | 3514 |
| 2551 | AIFM1 | apoptosis inducing factor mitochondria associated 1 | 9131 |
| 2552 | RAB5C | RAB5C, member RAS oncogene family | 5878 |
| 2553 | AP2B1 | adaptor related protein complex 2 subunit beta 1 | 163 |
| 2554 | KPNB1 | karyopherin subunit beta 1 | 3837 |
| 2555 | NUFIP2 | nuclear FMR1 interacting protein 2 | 57532 |
| 2556 | AIP | aryl hydrocarbon receptor interacting protein | 9049 |
| 2557 | ALDH2 | aldehyde dehydrogenase 2 family member | 217 |
| 2558 | FAF2 | Fas associated factor family member 2 | 23197 |
| 2559 | PGRMC2 | progesterone receptor membrane component 2 | 10424 |
| 2560 | MRPL15 | mitochondrial ribosomal protein L15 | 29088 |
| 2561 | ACAA1 | acetyl-CoA acyltransferase 1 | 30 |
| 2562 | FBL | fibrillarin | 2091 |
| 2563 | PSMA6 | proteasome subunit alpha 6 | 5687 |
| 2564 | TES | testin LIM domain protein | 26136 |
| 2565 | PLOD2 | procollagen-lysine,2-oxoglutarate 5-dioxygenase 2 | 5352 |
| 2566 | PPIB | peptidylprolyl isomerase B | 5479 |
| 2567 | ATP5F1C | ATP synthase F1 subunit gamma | 509 |
| 2568 | NPLOC4 | NPL4 homolog, ubiquitin recognition factor | 55666 |
| 2569 | NDUFS1 | NADH:ubiquinone oxidoreductase core subunit S1 | 4719 |
| 2570 | MCCC2 | methylcrotonoyl-CoA carboxylase 2 | 64087 |
| 2571 | HLA-F | major histocompatibility complex, class I, F | 3134 |
| 2572 | FKBP3 | FKBP prolyl isomerase 3 | 2287 |
| 2573 | BCAS2 | BCAS2, pre-mRNA processing factor | 10286 |
| 2574 | LUZP1 | leucine zipper protein 1 | 7798 |
| 2575 | PPIG | peptidylprolyl isomerase G | 9360 |
| 2576 | RGS10 | regulator of G protein signaling 10 | 6001 |
| 2577 | IMMT | inner membrane mitochondrial protein | 10989 |
| 2578 | AKAP8L | A-kinase anchoring protein 8 like | 26993 |
| 2579 | BLMH | bleomycin hydrolase | 642 |
| 2580 | SSRP1 | structure specific recognition protein 1 | 6749 |
| 2581 | AGPS | alkylglycerone phosphate synthase | 8540 |
| 2582 | SNRNP70 | small nuclear ribonucleoprotein U1 subunit 70 | 6625 |
| 2583 | MRI1 | methylthioribose-1-phosphate isomerase 1 | 84245 |
| 2584 | HSPH1 | heat shock protein family H (Hsp110) member 1 | 10808 |
| 2585 | GTF3C5 | general transcription factor IIIC subunit 5 | 9328 |
| 2586 | UTP15 | UTP15, small subunit processome component | 84135 |
| 2587 | PDLIM7 | PDZ and LIM domain 7 | 9260 |
| 2588 | TRMT1 | tRNA methyltransferase 1 | 55621 |
| 2589 | SRSF11 | serine and arginine rich splicing factor 11 | 9295 |
| 2590 | RMDN1 | regulator of microtubule dynamics 1 | 51115 |
| 2591 | GSPT1 | G1 to S phase transition 1 | 2935 |
| 2592 | OSBP | oxysterol binding protein | 5007 |
| 2593 | RCC2 | regulator of chromosome condensation 2 | 55920 |
| 2594 | PELP1 | proline, glutamate and leucine rich protein 1 | 27043 |
| 2595 | PDE12 | phosphodiesterase 12 | 201626 |
| 2596 | NLE1 | notchless homolog 1 | 54475 |
| 2597 | GNB2 | G protein subunit beta 2 | 2783 |
| 2598 | PSMD12 | proteasome 26S subunit, non-ATPase 12 | 5718 |
| 2599 | ERMP1 | endoplasmic reticulum metallopeptidase 1 | 79956 |
| 2600 | SKP1 | S-phase kinase associated protein 1 | 6500 |
| 2601 | DHTKD1 | dehydrogenase E1 and transketolase domain containing 1 | 55526 |
| 2602 | SUMF2 | sulfatase modifying factor 2 | 25870 |
| 2603 | ZNF598 | zinc finger protein 598 | 90850 |
| 2604 | AAR2 | AAR2 splicing factor homolog | 25980 |
| 2605 | ARPC1B | actin related protein 2/3 complex subunit 1B | 10095 |
| 2606 | SF3A2 | splicing factor 3a subunit 2 | 8175 |
| 2607 | ACIN1 | apoptotic chromatin condensation inducer 1 | 22985 |
| 2608 | STUB1 | STIP1 homology and U-box containing protein 1 | 10273 |
| 2609 | BTF3L4 | basic transcription factor 3 like 4 | 91408 |
| 2610 | WDR75 | WD repeat domain 75 | 84128 |
| 2611 | MBP | myelin basic protein | 4155 |
| 2612 | WASH2P | WAS protein family homolog 2, pseudogene | 375260 |
| 2613 | PNN | pinin, desmosome associated protein | 5411 |
| 2614 | AHNAK | AHNAK nucleoprotein | 79026 |
| 2615 | PPP4R1 | protein phosphatase 4 regulatory subunit 1 | 9989 |
| 2616 | ERAP2 | endoplasmic reticulum aminopeptidase 2 | 64167 |
| 2617 | EXOC4 | exocyst complex component 4 | 60412 |
| 2618 | HYOU1 | hypoxia up-regulated 1 | 10525 |
| 2619 | GALNT2 | polypeptide N-acetylgalactosaminyltransferase 2 | 2590 |
| 2620 | MCTS2P | malignant T cell amplified sequence 2, pseudogene | 1E+08 |
| 2621 | IFI16 | interferon gamma inducible protein 16 | 3428 |
| 2622 | CSE1L | chromosome segregation 1 like | 1434 |
| 2623 | LSM3 | LSM3 homolog, U6 small nuclear RNA and mRNA degradation associated | 27258 |
| 2624 | LRPPRC | leucine rich pentatricopeptide repeat containing | 10128 |
| 2625 | HMBS | hydroxymethylbilane synthase | 3145 |
| 2626 | RPL29 | ribosomal protein L29 | 6159 |
| 2627 | HTATIP2 | HIV-1 Tat interactive protein 2 | 10553 |
| 2628 | THUMPD1 | THUMP domain containing 1 | 55623 |
| 2629 | CLTA | clathrin light chain A | 1211 |
| 2630 | SRC | SRC proto-oncogene, non-receptor tyrosine kinase | 6714 |
| 2631 | HTRA2 | HtrA serine peptidase 2 | 27429 |
| 2632 | STX4 | syntaxin 4 | 6810 |
| 2633 | NT5C | 5', 3'-nucleotidase, cytosolic | 30833 |
| 2634 | PRKDC | protein kinase, DNA-activated, catalytic subunit | 5591 |
| 2635 | VAMP8 | vesicle associated membrane protein 8 | 8673 |
| 2636 | UNC45A | unc-45 myosin chaperone A | 55898 |
| 2637 | RAB23 | RAB23, member RAS oncogene family | 51715 |
| 2638 | DDOST | dolichyl-diphosphooligosaccharide--protein glycosyltransferase non-catalytic subunit | 1650 |
| 2639 | WBP4 | WW domain binding protein 4 | 11193 |
| 2640 | HMGB1 | high mobility group box 1 | 3146 |
| 2641 | CERS2 | ceramide synthase 2 | 29956 |
| 2642 | COMT | catechol-O-methyltransferase | 1312 |
| 2643 | XPNPEP1 | X-prolyl aminopeptidase 1 | 7511 |
| 2644 | TPM4 | tropomyosin 4 | 7171 |
| 2645 | TSN | translin | 7247 |
| 2646 | SDC1 | syndecan 1 | 6382 |
| 2647 | BASP1 | brain abundant membrane attached signal protein 1 | 10409 |
| 2648 | EIF3E | eukaryotic translation initiation factor 3 subunit E | 3646 |
| 2649 | TRMT6 | tRNA methyltransferase 6 | 51605 |
| 2650 | COPS8 | COP9 signalosome subunit 8 | 10920 |
| 2651 | CPD | carboxypeptidase D | 1362 |
| 2652 | TPR | translocated promoter region, nuclear basket protein | 7175 |
| 2653 | SCFD1 | sec1 family domain containing 1 | 23256 |
| 2654 | NUP98 | nucleoporin 98 | 4928 |
| 2655 | PRKAA1 | protein kinase AMP-activated catalytic subunit alpha 1 | 5562 |
| 2656 | STAM2 | signal transducing adaptor molecule 2 | 10254 |
| 2657 | CDKN2A | cyclin dependent kinase inhibitor 2A | 1029 |
| 2658 | RO60 | Ro60, Y RNA binding protein | 6738 |
| 2659 | DLGAP4 | DLG associated protein 4 | 22839 |
| 2660 | HERC4 | HECT and RLD domain containing E3 ubiquitin protein ligase 4 | 26091 |
| 2661 | EIF2A | eukaryotic translation initiation factor 2A | 83939 |
| 2662 | CAVIN3 | caveolae associated protein 3 | 112464 |
| 2663 | STRN3 | striatin 3 | 29966 |
| 2664 | UQCC2 | ubiquinol-cytochrome c reductase complex assembly factor 2 | 84300 |
| 2665 | STAT6 | signal transducer and activator of transcription 6 | 6778 |
| 2666 | STIM1 | stromal interaction molecule 1 | 6786 |
| 2667 | SNRPE | small nuclear ribonucleoprotein polypeptide E | 6635 |
| 2668 | MESD | mesoderm development LRP chaperone | 23184 |
| 2669 | LONP1 | lon peptidase 1, mitochondrial | 9361 |
| 2670 | KIF2C | kinesin family member 2C | 11004 |
| 2671 | PPP3R1 | protein phosphatase 3 regulatory subunit B, alpha | 5534 |
| 2672 | TUBB6 | tubulin beta 6 class V | 84617 |
| 2673 | JUP | junction plakoglobin | 3728 |
| 2674 | MTCH1 | mitochondrial carrier 1 | 23787 |
| 2675 | ACTR2 | actin related protein 2 | 10097 |
| 2676 | NT5E | 5'-nucleotidase ecto | 4907 |
| 2677 | PHF5A | PHD finger protein 5A | 84844 |
| 2678 | TMSB4X | thymosin beta 4 X-linked | 7114 |
| 2679 | PAFAH1B2 | platelet activating factor acetylhydrolase 1b catalytic subunit 2 | 5049 |
| 2680 | ATP6AP1 | ATPase H+ transporting accessory protein 1 | 537 |
| 2681 | COX5B | cytochrome c oxidase subunit 5B | 1329 |
| 2682 | EIF4B | eukaryotic translation initiation factor 4B | 1975 |
| 2683 | ARF6 | ADP ribosylation factor 6 | 382 |
| 2684 | ADNP | activity dependent neuroprotector homeobox | 23394 |
| 2685 | ISOC1 | isochorismatase domain containing 1 | 51015 |
| 2686 | RFC5 | replication factor C subunit 5 | 5985 |
| 2687 | FRYL | FRY like transcription coactivator | 285527 |
| 2688 | SAR1B | secretion associated Ras related GTPase 1B | 51128 |
| 2689 | STX7 | syntaxin 7 | 8417 |
| 2690 | AP1M1 | adaptor related protein complex 1 subunit mu 1 | 8907 |
| 2691 | ITGB6 | integrin subunit beta 6 | 3694 |
| 2692 | BZW1 | basic leucine zipper and W2 domains 1 | 9689 |
| 2693 | MVK | mevalonate kinase | 4598 |
| 2694 | SIRT5 | sirtuin 5 | 23408 |
| 2695 | HNRNPK | heterogeneous nuclear ribonucleoprotein K | 3190 |
| 2696 | SRSF10 | serine and arginine rich splicing factor 10 | 10772 |
| 2697 | IVL | involucrin | 3713 |
| 2698 | PPFIBP1 | PPFIA binding protein 1 | 8496 |
| 2699 | UGDH | UDP-glucose 6-dehydrogenase | 7358 |
| 2700 | MMS19 | MMS19 homolog, cytosolic iron-sulfur assembly component | 64210 |
| 2701 | SRA1 | steroid receptor RNA activator 1 | 10011 |
| 2702 | PRKCSH | protein kinase C substrate 80K-H | 5589 |
| 2703 | MAT2A | methionine adenosyltransferase 2A | 4144 |
| 2704 | GNB1 | G protein subunit beta 1 | 2782 |
| 2705 | FAM50A | family with sequence similarity 50 member A | 9130 |
| 2706 | HIBCH | 3-hydroxyisobutyryl-CoA hydrolase | 26275 |
| 2707 | OSBPL9 | oxysterol binding protein like 9 | 114883 |
| 2708 | TJAP1 | tight junction associated protein 1 | 93643 |
| 2709 | RHEB | Ras homolog, mTORC1 binding | 6009 |
| 2710 | FERMT1 | fermitin family member 1 | 55612 |
| 2711 | CLNS1A | chloride nucleotide-sensitive channel 1A | 1207 |
| 2712 | COMMD8 | COMM domain containing 8 | 54951 |
| 2713 | NDUFA5 | NADH:ubiquinone oxidoreductase subunit A5 | 4698 |
| 2714 | CDK5RAP3 | CDK5 regulatory subunit associated protein 3 | 80279 |
| 2715 | MRPL43 | mitochondrial ribosomal protein L43 | 84545 |
| 2716 | SUCLG2 | succinate-CoA ligase GDP-forming beta subunit | 8801 |
| 2717 | LPCAT2 | lysophosphatidylcholine acyltransferase 2 | 54947 |
| 2718 | CPT1A | carnitine palmitoyltransferase 1A | 1374 |
| 2719 | CAMSAP2 | calmodulin regulated spectrin associated protein family member 2 | 23271 |
| 2720 | EEF1E1 | eukaryotic translation elongation factor 1 epsilon 1 | 9521 |
| 2721 | SEC31A | SEC31 homolog A, COPII coat complex component | 22872 |
| 2722 | FKBP5 | FKBP prolyl isomerase 5 | 2289 |
| 2723 | RANGAP1 | Ran GTPase activating protein 1 | 5905 |
| 2724 | PPP1R2 | protein phosphatase 1 regulatory inhibitor subunit 2 | 5504 |
| 2725 | EEA1 | early endosome antigen 1 | 8411 |
| 2726 | DIP2B | disco interacting protein 2 homolog B | 57609 |
| 2727 | RRAS | RAS related | 6237 |
| 2728 | ATP5MPL | ATP synthase membrane subunit 6.8PL | 9556 |
| 2729 | PPP4R2 | protein phosphatase 4 regulatory subunit 2 | 151987 |
| 2730 | SAMHD1 | SAM and HD domain containing deoxynucleoside triphosphate triphosphohydrolase 1 | 25939 |
| 2731 | DDX20 | DEAD-box helicase 20 | 11218 |
| 2732 | SUMO1 | small ubiquitin-like modifier 1 | 7341 |
| 2733 | ISCU | iron-sulfur cluster assembly enzyme | 23479 |
| 2734 | CCDC22 | coiled-coil domain containing 22 | 28952 |
| 2735 | DUT | deoxyuridine triphosphatase | 1854 |
| 2736 | MAPK1 | mitogen-activated protein kinase 1 | 5594 |
| 2737 | P3H1 | prolyl 3-hydroxylase 1 | 64175 |
| 2738 | RPS29 | ribosomal protein S29 | 6235 |
| 2739 | TRMT112 | tRNA methyltransferase subunit 11-2 | 51504 |
| 2740 | ADI1 | acireductone dioxygenase 1 | 55256 |
| 2741 | EIF5B | eukaryotic translation initiation factor 5B | 9669 |
| 2742 | PURA | purine rich element binding protein A | 5813 |
| 2743 | SIN3A | SIN3 transcription regulator family member A | 25942 |
| 2744 | CSNK2A2 | casein kinase 2 alpha 2 | 1459 |
| 2745 | FAM160B1 | family with sequence similarity 160 member B1 | 57700 |
| 2746 | RPA1 | replication protein A1 | 6117 |
| 2747 | PALLD | palladin, cytoskeletal associated protein | 23022 |
| 2748 | CALM1 | calmodulin 1 | 801 |
| 2749 | ERH | ERH, mRNA splicing and mitosis factor | 2079 |
| 2750 | CYC1 | cytochrome c1 | 1537 |
| 2751 | SLFN5 | schlafen family member 5 | 162394 |
| 2752 | CORO2A | coronin 2A | 7464 |
| 2753 | MPI | mannose phosphate isomerase | 4351 |
| 2754 | HADHB | hydroxyacyl-CoA dehydrogenase trifunctional multienzyme complex subunit beta | 3032 |
| 2755 | FASN | fatty acid synthase | 2194 |
| 2756 | XRCC6 | X-ray repair cross complementing 6 | 2547 |
| 2757 | PLEC | plectin | 5339 |
| 2758 | PSMC5 | proteasome 26S subunit, ATPase 5 | 5705 |
| 2759 | PKP2 | plakophilin 2 | 5318 |
| 2760 | LSM4 | LSM4 homolog, U6 small nuclear RNA and mRNA degradation associated | 25804 |
| 2761 | IDH2 | isocitrate dehydrogenase (NADP(+)) 2, mitochondrial | 3418 |
| 2762 | RCN2 | reticulocalbin 2 | 5955 |
| 2763 | GABARAPL2 | GABA type A receptor associated protein like 2 | 11345 |
| 2764 | AHNAK2 | AHNAK nucleoprotein 2 | 113146 |
| 2765 | NDUFA4 | NDUFA4, mitochondrial complex associated | 4697 |
| 2766 | ELOC | elongin C | 6921 |
| 2767 | WASHC2C | WASH complex subunit 2C | 253725 |
| 2768 | GNA11 | G protein subunit alpha 11 | 2767 |
| 2769 | DNM2 | dynamin 2 | 1785 |
| 2770 | TOM1 | target of myb1 membrane trafficking protein | 10043 |
| 2771 | RPL35 | ribosomal protein L35 | 11224 |
| 2772 | DTD1 | D-tyrosyl-tRNA deacylase 1 | 92675 |
| 2773 | RICTOR | RPTOR independent companion of MTOR complex 2 | 253260 |
| 2774 | GGH | gamma-glutamyl hydrolase | 8836 |
| 2775 | FAM3C | family with sequence similarity 3 member C | 10447 |
| 2776 | SSB | Sjogren syndrome antigen B | 6741 |
| 2777 | PGM1 | phosphoglucomutase 1 | 5236 |
| 2778 | HNRNPM | heterogeneous nuclear ribonucleoprotein M | 4670 |
| 2779 | PSMD7 | proteasome 26S subunit, non-ATPase 7 | 5713 |
| 2780 | BET1 | Bet1 golgi vesicular membrane trafficking protein | 10282 |
| 2781 | GTF2B | general transcription factor IIB | 2959 |
| 2782 | HMGA1 | high mobility group AT-hook 1 | 3159 |
| 2783 | WASHC3 | WASH complex subunit 3 | 51019 |
| 2784 | PLXNA1 | plexin A1 | 5361 |
| 2785 | SLC22A5 | solute carrier family 22 member 5 | 6584 |
| 2786 | NPM3 | nucleophosmin/nucleoplasmin 3 | 10360 |
| 2787 | PREP | prolyl endopeptidase | 5550 |
| 2788 | SQOR | sulfide quinone oxidoreductase | 58472 |
| 2789 | RPS15 | ribosomal protein S15 | 6209 |
| 2790 | CDK2 | cyclin dependent kinase 2 | 1017 |
| 2791 | CRYBG1 | crystallin beta-gamma domain containing 1 | 202 |
| 2792 | CUL1 | cullin 1 | 8454 |
| 2793 | MCRIP1 | MAPK regulated corepressor interacting protein 1 | 348262 |
| 2794 | IDH3G | isocitrate dehydrogenase 3 (NAD(+)) gamma | 3421 |
| 2795 | RRM1 | ribonucleotide reductase catalytic subunit M1 | 6240 |
| 2796 | GDI1 | GDP dissociation inhibitor 1 | 2664 |
| 2797 | GLA | galactosidase alpha | 2717 |
| 2798 | DCD | dermcidin | 117159 |
| 2799 | RRAGC | Ras related GTP binding C | 64121 |
| 2800 | CWC27 | CWC27 spliceosome associated protein homolog | 10283 |
| 2801 | JPT1 | Jupiter microtubule associated homolog 1 | 51155 |
| 2802 | CAMK2D | calcium/calmodulin dependent protein kinase II delta | 817 |
| 2803 | DR1 | down-regulator of transcription 1 | 1810 |
| 2804 | CBR1 | carbonyl reductase 1 | 873 |
| 2805 | CARMIL1 | capping protein regulator and myosin 1 linker 1 | 55604 |
| 2806 | MAP7 | microtubule associated protein 7 | 9053 |
| 2807 | RPS21 | ribosomal protein S21 | 6227 |
| 2808 | COPS5 | COP9 signalosome subunit 5 | 10987 |
| 2809 | NONO | non-POU domain containing octamer binding | 4841 |
| 2810 | CC2D1A | coiled-coil and C2 domain containing 1A | 54862 |
| 2811 | AK6 | adenylate kinase 6 | 1.02E+08 |
| 2812 | ELMO3 | engulfment and cell motility 3 | 79767 |
| 2813 | FLNC | filamin C | 2318 |
| 2814 | SNRPC | small nuclear ribonucleoprotein polypeptide C | 6631 |
| 2815 | NENF | neudesin neurotrophic factor | 29937 |
| 2816 | EML4 | EMAP like 4 | 27436 |
| 2817 | ACAD9 | acyl-CoA dehydrogenase family member 9 | 28976 |
| 2818 | G3BP2 | G3BP stress granule assembly factor 2 | 9908 |
| 2819 | REPS1 | RALBP1 associated Eps domain containing 1 | 85021 |
| 2820 | DAZAP1 | DAZ associated protein 1 | 26528 |
| 2821 | MASTL | microtubule associated serine/threonine kinase like | 84930 |
| 2822 | SBDS | SBDS, ribosome maturation factor | 51119 |
| 2823 | PRPF40A | pre-mRNA processing factor 40 homolog A | 55660 |
| 2824 | CD99 | CD99 molecule (Xg blood group) | 4267 |
| 2825 | PDHX | pyruvate dehydrogenase complex component X | 8050 |
| 2826 | DHRS4 | dehydrogenase/reductase 4 | 10901 |
| 2827 | DCTPP1 | dCTP pyrophosphatase 1 | 79077 |
| 2828 | POLR1D | RNA polymerase I and III subunit D | 51082 |
| 2829 | AK3 | adenylate kinase 3 | 50808 |
| 2830 | ARF5 | ADP ribosylation factor 5 | 381 |
| 2831 | TRIM25 | tripartite motif containing 25 | 7706 |
| 2832 | STAMBP | STAM binding protein | 10617 |
| 2833 | SNTB2 | syntrophin beta 2 | 6645 |
| 2834 | RER1 | retention in endoplasmic reticulum sorting receptor 1 | 11079 |
| 2835 | SCAMP3 | secretory carrier membrane protein 3 | 10067 |
| 2836 | TIAL1 | TIA1 cytotoxic granule associated RNA binding protein like 1 | 7073 |
| 2837 | SRSF1 | serine and arginine rich splicing factor 1 | 6426 |
| 2838 | RRBP1 | ribosome binding protein 1 | 6238 |
| 2839 | WDR5 | WD repeat domain 5 | 11091 |
| 2840 | NEDD4L | neural precursor cell expressed, developmentally down-regulated 4-like, E3 ubiquitin protein ligase | 23327 |
| 2841 | NLN | neurolysin | 57486 |
| 2842 | RPL36 | ribosomal protein L36 | 25873 |
| 2843 | FAHD2A | fumarylacetoacetate hydrolase domain containing 2A | 51011 |
| 2844 | NCKAP1 | NCK associated protein 1 | 10787 |
| 2845 | RPE | ribulose-5-phosphate-3-epimerase | 6120 |
| 2846 | RAC1 | Rac family small GTPase 1 | 5879 |
| 2847 | NTMT1 | N-terminal Xaa-Pro-Lys N-methyltransferase 1 | 28989 |

**Supplementary Table 4:** List of regulated human proteins

| Control VS HK83 | | | |
| --- | --- | --- | --- |
| TOP accession | Anova (p) | Max fold change | Highest mean condition |
| S10A8_HUMAN | 0.00 | 2.89 | HK83 |
| HEMO_HUMAN | 0.00 | 2.09 | HK83 |
| H0Y507_HUMAN | 0.00 | 2.02 | Control |
| FADS2_HUMAN | 0.00 | 2.66 | Control |
| DNMT1_HUMAN | 0.00 | 2.07 | Control |
| UT14A_HUMAN | 0.00 | 3.48 | Control |
| SODM_HUMAN | 0.00 | 2.63 | HK83 |
| KRR1_HUMAN | 0.00 | 2.22 | Control |
| DDX56_HUMAN | 0.00 | 2.26 | Control |
| TSP1_HUMAN | 0.00 | 2.96 | Control |
| NSA2_HUMAN | 0.01 | 2.62 | Control |
| FAT2_HUMAN | 0.01 | 3.02 | Control |
| DIAP3_HUMAN | 0.01 | 2.05 | Control |
| IL1A_HUMAN | 0.01 | 6.43 | HK83 |
| RBM19_HUMAN | 0.01 | 2.68 | Control |
| A0A0C4DGN4_HUMAN | 0.01 | 16.36 | Control |
| RFC1_HUMAN | 0.01 | 2.53 | Control |
| TRM1L_HUMAN | 0.01 | 2.12 | Control |
| P63_HUMAN | 0.02 | 2.22 | Control |
| ACTG_HUMAN | 0.02 | 2.49 | HK83 |
| ACTB_HUMAN | 0.02 | 2.49 | HK83 |
| CADH3_HUMAN | 0.02 | 2.08 | Control |
| CRYAB_HUMAN | 0.02 | 2.33 | HK83 |
| CDSN_HUMAN | 0.02 | 7.61 | HK83 |
| sp\|P04264\|K2C1_HUMAN | 0.02 | 2.42 | HK83 |
| sp\|P13645\|K1C10_HUMAN | 0.02 | 2.13 | HK83 |
| NFKB2_HUMAN | 0.03 | 2.01 | HK83 |
| NUSAP_HUMAN | 0.03 | 2.13 | Control |
| A0A087WUX8_HUMAN | 0.04 | 2.01 | Control |
| ICAM1_HUMAN | 0.04 | 4.45 | HK83 |
| IL1B_HUMAN | 0.04 | 4.50 | HK83 |
| IL1RA_HUMAN | 0.04 | 3.08 | HK83 |
|  |  |  |  |
| Control VS JP2 | | | |
| TOP accession | Anova (p) | Max fold change | Highest mean condition |
| S10A8_HUMAN | 0.00 | 2.79 | JP2 |
| CYTB_HUMAN | 0.00 | 2.14 | JP2 |
| HEMO_HUMAN | 0.01 | 2.42 | JP2 |
| H0Y507_HUMAN | 0.00 | 2.07 | Control |
| FADS2_HUMAN | 0.00 | 3.49 | Control |
| UT14A_HUMAN | 0.00 | 5.41 | Control |
| KYNU_HUMAN | 0.00 | 2.67 | JP2 |
| SODM_HUMAN | 0.00 | 3.19 | JP2 |
| DDX56_HUMAN | 0.00 | 3.39 | Control |
| TSP1_HUMAN | 0.01 | 2.56 | Control |
| PSA5_HUMAN | 0.00 | 2.17 | JP2 |
| NSA2_HUMAN | 0.00 | 3.24 | Control |
| FAT2_HUMAN | 0.01 | 4.20 | Control |
| DIAP3_HUMAN | 0.00 | 4.00 | Control |
| IL1A_HUMAN | 0.00 | 9.33 | JP2 |
| RBM19_HUMAN | 0.00 | 9.62 | Control |
| RO52_HUMAN | 0.00 | 2.04 | JP2 |
| SYTM_HUMAN | 0.00 | 2.04 | Control |
| MK67I_HUMAN | 0.00 | 2.13 | Control |
| A0A0C4DGN4_HUMAN | 0.00 | 22.46 | Control |
| DDX24_HUMAN | 0.01 | 2.13 | Control |
| IDI1_HUMAN | 0.03 | 2.02 | JP2 |
| LARP4_HUMAN | 0.01 | 2.01 | Control |
| ACOD_HUMAN | 0.00 | 2.18 | Control |
| B7ZKJ8_HUMAN | 0.01 | 2.62 | JP2 |
| RL7L_HUMAN | 0.00 | 2.05 | Control |
| TRM1L_HUMAN | 0.00 | 3.55 | Control |
| P63_HUMAN | 0.00 | 2.77 | Control |
| C9J8Q1_HUMAN | 0.01 | 2.67 | Control |
| BYST_HUMAN | 0.00 | 2.90 | Control |
| ACTG_HUMAN | 0.00 | 2.77 | JP2 |
| ACTB_HUMAN | 0.00 | 2.77 | JP2 |
| TUFT1_HUMAN | 0.00 | 2.48 | JP2 |
| CRYAB_HUMAN | 0.00 | 3.51 | JP2 |
| CDSN_HUMAN | 0.04 | 4.27 | JP2 |
| A0A087WUV8_HUMAN | 0.04 | 2.16 | JP2 |
| MEPCE_HUMAN | 0.00 | 2.29 | Control |
| NFKB2_HUMAN | 0.00 | 3.41 | JP2 |
| 1433B_HUMAN | 0.01 | 2.16 | JP2 |
| C9IZG4_HUMAN | 0.03 | 2.10 | JP2 |
| SSRD_HUMAN | 0.01 | 2.53 | JP2 |
| A0A2R8YGH5_HUMAN | 0.01 | 2.60 | Control |
| S100P_HUMAN | 0.01 | 2.05 | JP2 |
| A0A087WUB9_HUMAN | 0.00 | 2.69 | Control |
| ICAM1_HUMAN | 0.00 | 4.92 | JP2 |
| PHLB1_HUMAN | 0.00 | 2.07 | Control |
| IL1B_HUMAN | 0.00 | 6.44 | JP2 |
| RTKN_HUMAN | 0.00 | 2.12 | JP2 |
| E7ES96_HUMAN | 0.00 | 2.29 | JP2 |
| IL1RA_HUMAN | 0.01 | 4.70 | JP2 |
| PKP1_HUMAN | 0.00 | 2.24 | Control |
| A0A087WSW9_HUMAN | 0.00 | 2.61 | JP2 |
| PDIA1_HUMAN | 0.02 | 2.63 | JP2 |
| CSN4_HUMAN | 0.00 | 2.09 | JP2 |
| TPBG_HUMAN | 0.00 | 2.29 | JP2 |
| S10A9_HUMAN | 0.00 | 2.67 | JP2 |
| DAG1_HUMAN | 0.01 | 2.75 | Control |
| CP2S1_HUMAN | 0.02 | 2.35 | JP2 |
| A0A0D9SGE8_HUMAN | 0.00 | 3.35 | Control |
| DDX10_HUMAN | 0.00 | 2.13 | Control |
| SMRC1_HUMAN | 0.00 | 2.46 | Control |
| TAGL_HUMAN | 0.01 | 2.55 | JP2 |
| FGFP1_HUMAN | 0.02 | 2.07 | Control |
| RRP7A_HUMAN | 0.00 | 3.65 | Control |
| PAI2_HUMAN | 0.01 | 3.92 | JP2 |
| A0A3B3ISS6_HUMAN | 0.01 | 2.68 | JP2 |
| CDCP1_HUMAN | 0.04 | 2.21 | JP2 |
| sp\|P35908\|K22E_HUMAN | 0.03 | 2.12 | JP2 |
| REEP5_HUMAN | 0.02 | 2.27 | Control |
| PDIA4_HUMAN | 0.00 | 2.02 | JP2 |
| ITA5_HUMAN | 0.00 | 2.07 | JP2 |
| LYN_HUMAN | 0.04 | 2.51 | JP2 |
| CAPR1_HUMAN | 0.00 | 2.19 | Control |
| A0A024R571_HUMAN | 0.03 | 2.14 | JP2 |
| E9PQY3_HUMAN | 0.00 | 2.23 | JP2 |
| RMD3_HUMAN | 0.01 | 2.02 | JP2 |
| NDUBB_HUMAN | 0.00 | 2.17 | Control |
| sp\|P02768\|ALBU_HUMAN | 0.04 | 3.77 | JP2 |
| H3BM67_HUMAN | 0.00 | 2.98 | Control |
| PFD6_HUMAN | 0.02 | 2.52 | JP2 |
| 1433E_HUMAN | 0.00 | 2.28 | JP2 |
| IRF6_HUMAN | 0.03 | 2.28 | JP2 |
| SFR15_HUMAN | 0.01 | 3.75 | Control |
| DLRB1_HUMAN | 0.00 | 2.07 | JP2 |
| CDC37_HUMAN | 0.01 | 2.11 | JP2 |
| GCDH_HUMAN | 0.04 | 2.14 | JP2 |
| LAMB3_HUMAN | 0.03 | 2.58 | JP2 |
| B8ZZD4_HUMAN | 0.00 | 2.22 | Control |
| EPHA2_HUMAN | 0.05 | 2.00 | JP2 |
| E9PQP1_HUMAN | 0.02 | 2.17 | JP2 |
| TB10B_HUMAN | 0.03 | 4.03 | Control |
| SPRE_HUMAN | 0.03 | 2.65 | JP2 |
| LRC59_HUMAN | 0.01 | 2.31 | JP2 |
| A0A087X117_HUMAN | 0.01 | 2.44 | JP2 |
| A0A087WT99_HUMAN | 0.01 | 2.65 | Control |
| SRP72_HUMAN | 0.00 | 2.05 | JP2 |
| D6RBW1_HUMAN | 0.05 | 2.04 | JP2 |
| LAMP2_HUMAN | 0.00 | 2.72 | JP2 |
| NDUA7_HUMAN | 0.03 | 2.61 | Control |
| LAMC2_HUMAN | 0.02 | 2.04 | JP2 |
| CCD86_HUMAN | 0.00 | 2.91 | Control |
| F2Z2Y4_HUMAN | 0.02 | 2.40 | JP2 |
| STX5_HUMAN | 0.03 | 2.20 | JP2 |
| NUDC3_HUMAN | 0.02 | 2.06 | Control |
| sp\|P02538\|K2C6A_HUMAN | 0.00 | 2.13 | JP2 |
| K7EIY6_HUMAN | 0.03 | 2.50 | Control |
| TMED2_HUMAN | 0.01 | 2.16 | JP2 |
| sp\|P12035\|K2C3_HUMAN | 0.00 | 2.38 | JP2 |
| H0Y614_HUMAN | 0.03 | 2.33 | JP2 |
| THUM1_HUMAN | 0.01 | 2.25 | Control |
| A0A0C4DGS1_HUMAN | 0.03 | 2.16 | JP2 |
| XPP1_HUMAN | 0.04 | 2.44 | JP2 |
| RRAS_HUMAN | 0.01 | 2.03 | JP2 |
| F16B1_HUMAN | 0.00 | 2.38 | Control |
|  |  |  |  |
| Control VS HK83 Δhcp | | | |
| TOP accession | Anova (p) | Max fold change | Highest mean condition |
| S10A8_HUMAN | 0.00 | 3.25 | HK83 Δhcp_test |
| CYTB_HUMAN | 0.00 | 2.36 | HK83 Δhcp_test |
| HEMO_HUMAN | 0.00 | 2.53 | HK83 Δhcp_test |
| WDR33_HUMAN | 0.00 | 2.40 | Control |
| FADS2_HUMAN | 0.00 | 5.49 | Control |
| DNMT1_HUMAN | 0.00 | 3.04 | Control |
| UT14A_HUMAN | 0.00 | 5.18 | Control |
| KYNU_HUMAN | 0.00 | 2.26 | HK83 Δhcp_test |
| A0A0C4DFL7_HUMAN | 0.00 | 3.21 | Control |
| SODM_HUMAN | 0.00 | 3.31 | HK83 Δhcp_test |
| KRR1_HUMAN | 0.01 | 2.08 | Control |
| TX1B3_HUMAN | 0.01 | 2.28 | HK83 Δhcp_test |
| RABP2_HUMAN | 0.00 | 2.15 | HK83 Δhcp_test |
| DDX56_HUMAN | 0.00 | 3.38 | Control |
| S38A2_HUMAN | 0.00 | 2.11 | HK83 Δhcp_test |
| NSA2_HUMAN | 0.00 | 3.69 | Control |
| FAT2_HUMAN | 0.01 | 2.38 | Control |
| IL1A_HUMAN | 0.00 | 8.40 | HK83 Δhcp_test |
| LARP7_HUMAN | 0.00 | 2.11 | Control |
| CRK_HUMAN | 0.00 | 2.04 | HK83 Δhcp_test |
| RBM19_HUMAN | 0.03 | 3.06 | Control |
| SYTM_HUMAN | 0.01 | 2.10 | Control |
| DPP2_HUMAN | 0.01 | 2.11 | HK83 Δhcp_test |
| IMUP_HUMAN | 0.03 | 2.05 | HK83 Δhcp_test |
| DDX24_HUMAN | 0.01 | 3.32 | Control |
| RFC1_HUMAN | 0.00 | 2.83 | Control |
| CARF_HUMAN | 0.00 | 2.04 | Control |
| ACOD_HUMAN | 0.00 | 2.13 | Control |
| IF6_HUMAN | 0.01 | 2.06 | HK83 Δhcp_test |
| B7ZKJ8_HUMAN | 0.00 | 3.03 | HK83 Δhcp_test |
| TRM1L_HUMAN | 0.00 | 2.91 | Control |
| P63_HUMAN | 0.00 | 3.16 | Control |
| E9PM90_HUMAN | 0.04 | 2.20 | HK83 Δhcp_test |
| BYST_HUMAN | 0.00 | 4.02 | Control |
| ACTG_HUMAN | 0.01 | 2.91 | HK83 Δhcp_test |
| ACTB_HUMAN | 0.01 | 2.91 | HK83 Δhcp_test |
| ENOPH_HUMAN | 0.00 | 2.04 | HK83 Δhcp_test |
| A8MUM1_HUMAN | 0.01 | 2.69 | HK83 Δhcp_test |
| TPX2_HUMAN | 0.00 | 2.49 | Control |
| TUFT1_HUMAN | 0.00 | 2.42 | HK83 Δhcp_test |
| CRYAB_HUMAN | 0.01 | 2.67 | HK83 Δhcp_test |
| COF1_HUMAN | 0.01 | 2.24 | HK83 Δhcp_test |
| A0A087WWF6_HUMAN | 0.00 | 2.57 | Control |
| NUSAP_HUMAN | 0.02 | 2.12 | Control |
| SSRD_HUMAN | 0.00 | 2.29 | HK83 Δhcp_test |
| GFPT1_HUMAN | 0.04 | 2.17 | HK83 Δhcp_test |
| NDRG1_HUMAN | 0.00 | 2.20 | HK83 Δhcp_test |
| S100P_HUMAN | 0.00 | 2.38 | HK83 Δhcp_test |
| J3QSV6_HUMAN | 0.00 | 3.06 | Control |
| ICAM1_HUMAN | 0.00 | 4.88 | HK83 Δhcp_test |
| ADDA_HUMAN | 0.00 | 2.87 | Control |
| PHLB1_HUMAN | 0.00 | 2.77 | Control |
| IL1B_HUMAN | 0.00 | 6.67 | HK83 Δhcp_test |
| NICA_HUMAN | 0.00 | 2.09 | HK83 Δhcp_test |
| RRP1_HUMAN | 0.01 | 2.31 | Control |
| IL1RA_HUMAN | 0.01 | 3.12 | HK83 Δhcp_test |
| RRP12_HUMAN | 0.01 | 2.35 | Control |
| CCNB1_HUMAN | 0.00 | 2.56 | Control |
| I3L397_HUMAN | 0.00 | 2.22 | HK83 Δhcp_test |
| DDX54_HUMAN | 0.00 | 2.56 | Control |
| PKP1_HUMAN | 0.00 | 2.69 | Control |
| RAP2B_HUMAN | 0.01 | 2.17 | HK83 Δhcp_test |
| GAR1_HUMAN | 0.01 | 2.12 | Control |
| MGME1_HUMAN | 0.00 | 2.02 | Control |
| BIP_HUMAN | 0.01 | 2.02 | HK83 Δhcp_test |
| CL029_HUMAN | 0.01 | 3.09 | Control |
| CP2S1_HUMAN | 0.02 | 2.80 | HK83 Δhcp_test |
| A0A0D9SGE8_HUMAN | 0.03 | 3.16 | Control |
| A6NEL0_HUMAN | 0.00 | 2.64 | Control |
| CDC20_HUMAN | 0.00 | 4.02 | Control |
| TAGL_HUMAN | 0.00 | 2.09 | HK83 Δhcp_test |
| RT4I1_HUMAN | 0.01 | 3.29 | Control |
| FGFP1_HUMAN | 0.01 | 3.08 | Control |
| PLSL_HUMAN | 0.03 | 2.02 | HK83 Δhcp_test |
| RRP7A_HUMAN | 0.02 | 2.43 | Control |
| PIGR_HUMAN | 0.04 | 2.62 | Control |
| A0A0A0MRW6_HUMAN | 0.01 | 2.17 | Control |
| DYR_HUMAN | 0.00 | 2.47 | Control |
| A0A0A6YYI3_HUMAN | 0.01 | 2.04 | Control |
| PAI2_HUMAN | 0.01 | 4.14 | HK83 Δhcp_test |
| D3YTB1_HUMAN | 0.00 | 2.03 | Control |
| RGAP1_HUMAN | 0.02 | 2.74 | Control |
| CDCP1_HUMAN | 0.01 | 2.19 | HK83 Δhcp_test |
| ITA5_HUMAN | 0.00 | 2.05 | HK83 Δhcp_test |
| DNLI1_HUMAN | 0.00 | 3.05 | Control |
| LGUL_HUMAN | 0.00 | 2.04 | HK83 Δhcp_test |
| TENS4_HUMAN | 0.00 | 2.12 | Control |
| CH10_HUMAN | 0.00 | 2.12 | HK83 Δhcp_test |
| TYSY_HUMAN | 0.00 | 2.26 | Control |
| A0A2R8YDM2_HUMAN | 0.01 | 2.60 | Control |
| RT09_HUMAN | 0.01 | 2.21 | Control |
| 1433E_HUMAN | 0.00 | 2.14 | HK83 Δhcp_test |
| BI2L1_HUMAN | 0.01 | 2.12 | Control |
| A0A087WZG4_HUMAN | 0.01 | 2.18 | Control |
| IFIT1_HUMAN | 0.01 | 2.03 | Control |
| DLRB1_HUMAN | 0.04 | 2.12 | HK83 Δhcp_test |
| A0A0A0MTB8_HUMAN | 0.02 | 2.28 | Control |
| MPP10_HUMAN | 0.00 | 2.03 | Control |
| LAMB3_HUMAN | 0.02 | 2.01 | HK83 Δhcp_test |
| SPS2L_HUMAN | 0.00 | 2.25 | Control |
| RL34_HUMAN | 0.01 | 4.17 | Control |
| GPN1_HUMAN | 0.00 | 2.18 | Control |
| LYAR_HUMAN | 0.00 | 2.07 | Control |
| H7BYN4_HUMAN | 0.02 | 2.17 | Control |
| MRPP3_HUMAN | 0.00 | 3.23 | Control |
| HIP1R_HUMAN | 0.04 | 2.02 | Control |
| PRP16_HUMAN | 0.00 | 2.31 | Control |
| NPL4_HUMAN | 0.02 | 2.04 | HK83 Δhcp_test |
| DHTK1_HUMAN | 0.01 | 2.06 | Control |
|  |  |  |  |
| Control VS HK83 Δtle5 Δglh | | | |
| TOP accession | Anova (p) | Max fold change | Highest mean condition |
| S10A8_HUMAN | 0.00 | 2.18 | HK83 Δtle5 Δglh |
| HEMO_HUMAN | 0.00 | 3.19 | HK83 Δtle5 Δglh |
| H0Y507_HUMAN | 0.00 | 2.11 | Control |
| WDR33_HUMAN | 0.00 | 2.16 | Control |
| FADS2_HUMAN | 0.00 | 2.52 | Control |
| UT14A_HUMAN | 0.00 | 2.98 | Control |
| A0A0C4DFL7_HUMAN | 0.00 | 2.00 | Control |
| SODM_HUMAN | 0.00 | 2.71 | HK83 Δtle5 Δglh |
| KRR1_HUMAN | 0.01 | 2.11 | Control |
| RRP1B_HUMAN | 0.00 | 2.19 | Control |
| S38A2_HUMAN | 0.00 | 2.47 | HK83 Δtle5 Δglh |
| PSA5_HUMAN | 0.00 | 2.23 | HK83 Δtle5 Δglh |
| NSA2_HUMAN | 0.00 | 2.21 | Control |
| FAT2_HUMAN | 0.01 | 2.27 | Control |
| IL1A_HUMAN | 0.00 | 6.73 | HK83 Δtle5 Δglh |
| RBM19_HUMAN | 0.01 | 3.27 | Control |
| A0A0C4DGN4_HUMAN | 0.04 | 9.16 | Control |
| DDX24_HUMAN | 0.02 | 2.16 | Control |
| MDC1_HUMAN | 0.00 | 2.35 | Control |
| RFC1_HUMAN | 0.00 | 2.94 | Control |
| ACOD_HUMAN | 0.00 | 2.13 | Control |
| B7ZKJ8_HUMAN | 0.00 | 3.17 | HK83 Δtle5 Δglh |
| TRM1L_HUMAN | 0.01 | 2.13 | Control |
| ACTG_HUMAN | 0.00 | 2.86 | HK83 Δtle5 Δglh |
| ACTB_HUMAN | 0.00 | 2.86 | HK83 Δtle5 Δglh |
| A8MUM1_HUMAN | 0.01 | 2.34 | HK83 Δtle5 Δglh |
| CRYAB_HUMAN | 0.00 | 3.50 | HK83 Δtle5 Δglh |
| CDSN_HUMAN | 0.02 | 134.01 | HK83 Δtle5 Δglh |
| MEPCE_HUMAN | 0.00 | 2.22 | Control |
| COF1_HUMAN | 0.04 | 2.00 | HK83 Δtle5 Δglh |
| NFKB2_HUMAN | 0.00 | 2.51 | HK83 Δtle5 Δglh |
| A0A087WWF6_HUMAN | 0.00 | 2.92 | Control |
| RRS1_HUMAN | 0.00 | 2.18 | Control |
| SSRD_HUMAN | 0.01 | 2.07 | HK83 Δtle5 Δglh |
| A0A0A0MRZ4_HUMAN | 0.01 | 2.94 | HK83 Δtle5 Δglh |
| J3QSV6_HUMAN | 0.00 | 2.10 | Control |
| ICAM1_HUMAN | 0.02 | 3.52 | HK83 Δtle5 Δglh |
| PHLB1_HUMAN | 0.01 | 2.06 | Control |
| IL1B_HUMAN | 0.00 | 4.23 | HK83 Δtle5 Δglh |
| E7ES96_HUMAN | 0.03 | 2.26 | HK83 Δtle5 Δglh |
| RRP1_HUMAN | 0.00 | 2.02 | Control |
| IL1RA_HUMAN | 0.01 | 2.83 | HK83 Δtle5 Δglh |
| A0A087WUQ6_HUMAN | 0.02 | 2.21 | HK83 Δtle5 Δglh |
| 1A66_HUMAN | 0.00 | 2.04 | HK83 Δtle5 Δglh |
| LMNB2_HUMAN | 0.03 | 2.44 | HK83 Δtle5 Δglh |
| C9JBI3_HUMAN | 0.01 | 2.02 | HK83 Δtle5 Δglh |
| CALL5_HUMAN | 0.03 | 4.76 | HK83 Δtle5 Δglh |
| CP2S1_HUMAN | 0.02 | 2.98 | HK83 Δtle5 Δglh |
| A0A0D9SGE8_HUMAN | 0.00 | 3.32 | Control |
| CDC20_HUMAN | 0.01 | 2.27 | Control |
| A0A087WXS7_HUMAN | 0.03 | 2.13 | HK83 Δtle5 Δglh |
| GARS_HUMAN | 0.00 | 2.12 | HK83 Δtle5 Δglh |
| TAGL_HUMAN | 0.00 | 2.12 | HK83 Δtle5 Δglh |
| RRP7A_HUMAN | 0.01 | 4.11 | Control |
| PAI2_HUMAN | 0.00 | 3.24 | HK83 Δtle5 Δglh |
| 1A02_HUMAN | 0.01 | 2.21 | HK83 Δtle5 Δglh |
| FRIH_HUMAN | 0.00 | 2.03 | HK83 Δtle5 Δglh |
| REEP5_HUMAN | 0.02 | 2.11 | Control |
| PDIA4_HUMAN | 0.00 | 2.13 | HK83 Δtle5 Δglh |
| DNLI1_HUMAN | 0.00 | 2.29 | Control |
| CHRD1_HUMAN | 0.02 | 2.15 | Control |
| CASPE_HUMAN | 0.03 | 3.81 | HK83 Δtle5 Δglh |
| A0A2R8YDM2_HUMAN | 0.00 | 2.02 | Control |
| Q6P0N6_HUMAN | 0.03 | 2.27 | Control |
| H3BM67_HUMAN | 0.02 | 2.26 | Control |
| A0A0A0MQX8_HUMAN | 0.02 | 2.45 | Control |
| STX6_HUMAN | 0.02 | 2.07 | HK83 Δtle5 Δglh |
| C9JEV0_HUMAN | 0.04 | 5.14 | HK83 Δtle5 Δglh |
| SDE2_HUMAN | 0.01 | 2.19 | Control |
| HBB_HUMAN | 0.03 | 3.49 | HK83 Δtle5 Δglh |
| LAMB3_HUMAN | 0.01 | 2.64 | HK83 Δtle5 Δglh |
| E7EW05_HUMAN | 0.04 | 3.04 | HK83 Δtle5 Δglh |
| EI24_HUMAN | 0.02 | 2.34 | Control |
| TB10B_HUMAN | 0.01 | 2.48 | Control |
| NPC1_HUMAN | 0.00 | 2.33 | HK83 Δtle5 Δglh |
| RL34_HUMAN | 0.01 | 3.64 | Control |
| GPN1_HUMAN | 0.01 | 2.12 | Control |
| MRCKB_HUMAN | 0.01 | 2.09 | Control |
| LAMC2_HUMAN | 0.01 | 2.53 | HK83 Δtle5 Δglh |
| LYAR_HUMAN | 0.02 | 2.02 | Control |
| STX5_HUMAN | 0.01 | 2.03 | HK83 Δtle5 Δglh |
| HBA_HUMAN | 0.05 | 2.47 | HK83 Δtle5 Δglh |
| NEP1_HUMAN | 0.01 | 2.82 | Control |
| KIF4A_HUMAN | 0.01 | 2.26 | Control |
| A0A0A0MRX2_HUMAN | 0.00 | 2.10 | HK83 Δtle5 Δglh |
| A0A0G2JH66_HUMAN | 0.02 | 2.19 | HK83 Δtle5 Δglh |
| PHF5A_HUMAN | 0.04 | 3.37 | Control |
| F16B1_HUMAN | 0.00 | 2.34 | Control |
| DAZP1_HUMAN | 0.01 | 2.37 | HK83 Δtle5 Δglh |
|  |  |  |  |
| Control VS HK83+JP2 | | | |
| TOP accession | Anova (p) | Max fold change | Highest mean condition |
| A0A0C4DGN4_HUMAN | 0.02 | 12.32 | Control |
| ACTG_HUMAN | 0.03 | 2.29 | HK83+JP2 |
| ACTB_HUMAN | 0.03 | 2.29 | HK83+JP2 |
| A8MUM1_HUMAN | 0.02 | 2.29 | HK83+JP2 |
| CRYAB_HUMAN | 0.02 | 2.14 | HK83+JP2 |
| A0A0A0MRZ4_HUMAN | 0.01 | 2.04 | HK83+JP2 |
| A0A0D9SGE8_HUMAN | 0.03 | 2.18 | Control |
| A6NEL0_HUMAN | 0.01 | 2.15 | Control |
| REEP5_HUMAN | 0.03 | 2.00 | Control |
| SFR15_HUMAN | 0.01 | 2.42 | Control |
| H4_HUMAN | 0.04 | 2.07 | Control |
| D6REL8_HUMAN | 0.03 | 2.14 | Control |
|  |  |  |  |
| Control VS HK83 Δhcp+JP2 | | | |
| TOP accession | Anova (p) | Max fold change | Highest mean condition |
| S10A8_HUMAN | 0.00 | 2.22 | HK83 Δhcp_JP2 |
| HEMO_HUMAN | 0.00 | 2.56 | HK83 Δhcp_JP2 |
| FADS2_HUMAN | 0.00 | 3.93 | Control |
| DNMT1_HUMAN | 0.00 | 2.38 | Control |
| UT14A_HUMAN | 0.00 | 3.94 | Control |
| KYNU_HUMAN | 0.00 | 2.05 | HK83 Δhcp_JP2 |
| A0A0C4DFL7_HUMAN | 0.00 | 2.70 | Control |
| SODM_HUMAN | 0.00 | 2.78 | HK83 Δhcp_JP2 |
| DDX56_HUMAN | 0.00 | 3.20 | Control |
| NSA2_HUMAN | 0.01 | 2.53 | Control |
| FAT2_HUMAN | 0.01 | 2.61 | Control |
| DIAP3_HUMAN | 0.00 | 2.06 | Control |
| IL1A_HUMAN | 0.00 | 6.44 | HK83 Δhcp_JP2 |
| RBM19_HUMAN | 0.01 | 2.84 | Control |
| DDX24_HUMAN | 0.00 | 2.77 | Control |
| MDC1_HUMAN | 0.00 | 3.17 | Control |
| RFC1_HUMAN | 0.01 | 2.23 | Control |
| B7ZKJ8_HUMAN | 0.00 | 2.53 | HK83 Δhcp_JP2 |
| TRM1L_HUMAN | 0.00 | 2.41 | Control |
| P63_HUMAN | 0.00 | 2.30 | Control |
| BYST_HUMAN | 0.00 | 2.77 | Control |
| CADH3_HUMAN | 0.00 | 2.39 | Control |
| ZMYM2_HUMAN | 0.00 | 2.62 | Control |
| A0A0A0MQR2_HUMAN | 0.00 | 2.04 | Control |
| CRYAB_HUMAN | 0.01 | 2.21 | HK83 Δhcp_JP2 |
| I2BP2_HUMAN | 0.00 | 2.33 | Control |
| GPC1_HUMAN | 0.00 | 2.60 | Control |
| NFKB2_HUMAN | 0.01 | 2.06 | HK83 Δhcp_JP2 |
| A0A087WWF6_HUMAN | 0.00 | 2.91 | Control |
| J3QSV6_HUMAN | 0.00 | 2.29 | Control |
| ICAM1_HUMAN | 0.00 | 5.18 | HK83 Δhcp_JP2 |
| ADDA_HUMAN | 0.00 | 2.23 | Control |
| PHLB1_HUMAN | 0.00 | 2.72 | Control |
| IL1B_HUMAN | 0.01 | 3.86 | HK83 Δhcp_JP2 |
| IL1RA_HUMAN | 0.01 | 2.57 | HK83 Δhcp_JP2 |
| DAG1_HUMAN | 0.00 | 2.55 | Control |
| CL029_HUMAN | 0.01 | 2.24 | Control |
| CP2S1_HUMAN | 0.03 | 2.29 | HK83 Δhcp_JP2 |
| A6NEL0_HUMAN | 0.00 | 2.38 | Control |
| CDC20_HUMAN | 0.02 | 2.25 | Control |
| B3KTM8_HUMAN | 0.02 | 2.13 | Control |
| A0A0A0MRW6_HUMAN | 0.01 | 2.08 | Control |
| DYR_HUMAN | 0.01 | 2.03 | Control |
| PAI2_HUMAN | 0.01 | 2.52 | HK83 Δhcp_JP2 |
| J3KTL8_HUMAN | 0.04 | 2.32 | Control |
| A0A2R8YDM2_HUMAN | 0.00 | 2.10 | Control |
| SDE2_HUMAN | 0.03 | 2.67 | Control |
| BRE1B_HUMAN | 0.01 | 2.70 | Control |
| DSC3_HUMAN | 0.02 | 3.40 | Control |
| RL34_HUMAN | 0.00 | 5.38 | Control |
| GPN1_HUMAN | 0.01 | 2.23 | Control |
| KIF4A_HUMAN | 0.00 | 3.04 | Control |
|  |  |  |  |
| Control VS HK83 Δtle5 Δglh+JP2 | | | |
| TOP accession | Anova (p) | Max fold change | Highest mean condition |
| HEMO_HUMAN | 0.00 | 2.83 | HK83 Δtle5 Δglh+JP2 |
| H0Y507_HUMAN | 0.00 | 2.35 | Control |
| FADS2_HUMAN | 0.00 | 3.14 | Control |
| UT14A_HUMAN | 0.00 | 5.67 | Control |
| SODM_HUMAN | 0.00 | 2.88 | HK83 Δtle5 Δglh+JP2 |
| KRR1_HUMAN | 0.02 | 2.14 | Control |
| DDX56_HUMAN | 0.00 | 3.19 | Control |
| TSP1_HUMAN | 0.04 | 2.05 | Control |
| NSA2_HUMAN | 0.00 | 2.32 | Control |
| FAT2_HUMAN | 0.00 | 2.60 | Control |
| DIAP3_HUMAN | 0.00 | 3.51 | Control |
| IL1A_HUMAN | 0.00 | 7.30 | HK83 Δtle5 Δglh+JP2 |
| RBM19_HUMAN | 0.01 | 4.44 | Control |
| MK67I_HUMAN | 0.00 | 2.04 | Control |
| DDX24_HUMAN | 0.00 | 3.02 | Control |
| MDC1_HUMAN | 0.00 | 2.04 | Control |
| RFC1_HUMAN | 0.00 | 3.70 | Control |
| LARP4_HUMAN | 0.00 | 2.33 | Control |
| B7ZKJ8_HUMAN | 0.00 | 3.08 | HK83 Δtle5 Δglh+JP2 |
| TRM1L_HUMAN | 0.00 | 2.43 | Control |
| P63_HUMAN | 0.00 | 2.81 | Control |
| BYST_HUMAN | 0.00 | 2.19 | Control |
| ACTG_HUMAN | 0.00 | 2.97 | HK83 Δtle5 Δglh+JP2 |
| ACTB_HUMAN | 0.00 | 2.97 | HK83 Δtle5 Δglh+JP2 |
| A8MUM1_HUMAN | 0.02 | 2.59 | HK83 Δtle5 Δglh+JP2 |
| ZMYM2_HUMAN | 0.00 | 2.72 | Control |
| TUFT1_HUMAN | 0.04 | 2.12 | HK83 Δtle5 Δglh+JP2 |
| CRYAB_HUMAN | 0.01 | 2.67 | HK83 Δtle5 Δglh+JP2 |
| CDSN_HUMAN | 0.05 | 13.44 | HK83 Δtle5 Δglh+JP2 |
| I2BP2_HUMAN | 0.00 | 2.25 | Control |
| COF1_HUMAN | 0.01 | 2.04 | HK83 Δtle5 Δglh+JP2 |
| NFKB2_HUMAN | 0.00 | 2.36 | HK83 Δtle5 Δglh+JP2 |
| A0A087WWF6_HUMAN | 0.02 | 2.03 | Control |
| A0A0A0MRZ4_HUMAN | 0.03 | 2.08 | HK83 Δtle5 Δglh+JP2 |
| S100P_HUMAN | 0.00 | 2.07 | HK83 Δtle5 Δglh+JP2 |
| J3QSV6_HUMAN | 0.00 | 2.04 | Control |
| ICAM1_HUMAN | 0.00 | 4.41 | HK83 Δtle5 Δglh+JP2 |
| ADDA_HUMAN | 0.00 | 2.22 | Control |
| PHLB1_HUMAN | 0.01 | 2.04 | Control |
| IL1B_HUMAN | 0.01 | 4.71 | HK83 Δtle5 Δglh+JP2 |
| E7ES96_HUMAN | 0.00 | 2.07 | HK83 Δtle5 Δglh+JP2 |
| RRP1_HUMAN | 0.00 | 2.21 | Control |
| IL1RA_HUMAN | 0.01 | 2.97 | HK83 Δtle5 Δglh+JP2 |
| PKP1_HUMAN | 0.01 | 2.54 | Control |
| RM55_HUMAN | 0.00 | 2.67 | Control |
| PDS5A_HUMAN | 0.00 | 2.52 | Control |
| CSN4_HUMAN | 0.00 | 2.06 | HK83 Δtle5 Δglh+JP2 |
| S10A9_HUMAN | 0.00 | 3.26 | HK83 Δtle5 Δglh+JP2 |
| LMNB2_HUMAN | 0.00 | 2.20 | HK83 Δtle5 Δglh+JP2 |
| DAG1_HUMAN | 0.01 | 2.66 | Control |
| CP2S1_HUMAN | 0.02 | 2.33 | HK83 Δtle5 Δglh+JP2 |
| A0A0D9SGE8_HUMAN | 0.00 | 2.78 | Control |
| DDX10_HUMAN | 0.00 | 2.19 | Control |
| MMAB_HUMAN | 0.04 | 2.01 | HK83 Δtle5 Δglh+JP2 |
| CDC20_HUMAN | 0.01 | 2.41 | Control |
| TAGL_HUMAN | 0.00 | 2.22 | HK83 Δtle5 Δglh+JP2 |
| RT4I1_HUMAN | 0.00 | 2.06 | Control |
| PAI2_HUMAN | 0.00 | 2.93 | HK83 Δtle5 Δglh+JP2 |
| D3YTB1_HUMAN | 0.01 | 2.13 | Control |
| REEP5_HUMAN | 0.01 | 2.54 | Control |
| DNLI1_HUMAN | 0.01 | 2.72 | Control |
| TYSY_HUMAN | 0.01 | 2.11 | Control |
| RIOK1_HUMAN | 0.00 | 2.08 | Control |
| Q6P0N6_HUMAN | 0.02 | 2.30 | Control |
| TACO1_HUMAN | 0.04 | 2.02 | Control |
| A0A0A0MQX8_HUMAN | 0.00 | 3.44 | Control |
| CYTC_HUMAN | 0.02 | 2.01 | Control |
| PTN6_HUMAN | 0.03 | 2.01 | HK83 Δtle5 Δglh+JP2 |
| E7ETU5_HUMAN | 0.01 | 2.04 | Control |
| HBB_HUMAN | 0.05 | 2.66 | HK83 Δtle5 Δglh+JP2 |
| E7EW05_HUMAN | 0.01 | 2.33 | HK83 Δtle5 Δglh+JP2 |
| A0A2R8YF87_HUMAN | 0.02 | 2.04 | Control |
| MGST3_HUMAN | 0.01 | 2.20 | Control |
| A0A087WT99_HUMAN | 0.00 | 3.24 | Control |
| COMD9_HUMAN | 0.00 | 2.22 | Control |
| RM51_HUMAN | 0.03 | 2.05 | Control |
| HBA_HUMAN | 0.03 | 2.49 | HK83 Δtle5 Δglh+JP2 |
| RBX1_HUMAN | 0.00 | 2.24 | Control |
| NEP1_HUMAN | 0.01 | 2.76 | Control |
| K7EIY6_HUMAN | 0.01 | 3.43 | Control |
| TM192_HUMAN | 0.01 | 2.02 | HK83 Δtle5 Δglh+JP2 |
| KIF4A_HUMAN | 0.01 | 2.23 | Control |
| sp\|P12035\|K2C3_HUMAN | 0.01 | 2.01 | HK83 Δtle5 Δglh+JP2 |
| NTM1A_HUMAN | 0.02 | 2.51 | Control |
|  |  |  |  |
| HK83 VS HK83 Δhcp | | | |
| FADS2_HUMAN | 0.04 | 2.06 | HK83 |
| BYST_HUMAN | 0.05 | 2.04 | HK83 |
| sp\|P13645\|K1C10_HUMAN | 0.02 | 2.10 | HK83 |
| A6NEL0_HUMAN | 0.00 | 2.01 | HK83 |
| CDC20_HUMAN | 0.00 | 2.51 | HK83 |
| RT4I1_HUMAN | 0.03 | 2.30 | HK83 |
| AAKG1_HUMAN | 0.04 | 2.27 | HK83 |
| H7BYF2_HUMAN | 0.02 | 2.23 | HK83 |
| OCAD1_HUMAN | 0.00 | 2.07 | HK83 Δhcp_test |
| H7BYN4_HUMAN | 0.02 | 2.02 | HK83 |
| MRPP3_HUMAN | 0.00 | 3.17 | HK83 |
| HIP1R_HUMAN | 0.01 | 2.10 | HK83 |
| PRP16_HUMAN | 0.01 | 2.22 | HK83 |
| DHTK1_HUMAN | 0.00 | 2.08 | HK83 |
| PHF5A_HUMAN | 0.04 | 3.44 | HK83 |
|  |  |  |  |
| HK83 VS HK83 Δtle5 Δglh | | | |
| TOP accession | Anova (p) | Max fold change | Highest mean condition |
| TSP1_HUMAN | 0.01 | 2.15 | HK83 Δtle5 Δglh |
| E7EW05_HUMAN | 0.01 | 3.75 | HK83 Δtle5 Δglh |
| BGH3_HUMAN | 0.00 | 2.13 | HK83 Δtle5 Δglh |
| TB10B_HUMAN | 0.03 | 2.20 | HK83 |
| PXDN_HUMAN | 0.04 | 2.08 | HK83 Δtle5 Δglh |
| NEP1_HUMAN | 0.02 | 2.51 | HK83 |
| KIF4A_HUMAN | 0.02 | 2.23 | HK83 |
| Q5LJA5_HUMAN | 0.01 | 2.38 | HK83 Δtle5 Δglh |
| RIOX1_HUMAN | 0.02 | 2.08 | HK83 |
| A0A0A0MRX2_HUMAN | 0.00 | 2.03 | HK83 Δtle5 Δglh |
| NPS3A_HUMAN | 0.05 | 2.31 | HK83 |
| PHF5A_HUMAN | 0.02 | 2.99 | HK83 |
| F16B1_HUMAN | 0.00 | 2.30 | HK83 |
| DAZP1_HUMAN | 0.01 | 2.37 | HK83 Δtle5 Δglh |
|  |  |  |  |
| HK83+JP2 VS HK83 Δhcp+JP2 | | | |
| TOP accession | Anova (p) | Max fold change | Highest mean condition |
| FADS2_HUMAN | 0.00 | 3.36 | HK83+JP2 |
| DNMT1_HUMAN | 0.00 | 2.05 | HK83+JP2 |
| UT14A_HUMAN | 0.01 | 2.66 | HK83+JP2 |
| A0A0C4DFL7_HUMAN | 0.00 | 2.37 | HK83+JP2 |
| DDX56_HUMAN | 0.00 | 2.94 | HK83+JP2 |
| S38A2_HUMAN | 0.00 | 2.01 | HK83 Δhcp_JP2 |
| FAT2_HUMAN | 0.03 | 2.23 | HK83+JP2 |
| DIAP3_HUMAN | 0.00 | 2.17 | HK83+JP2 |
| IL1A_HUMAN | 0.00 | 4.11 | HK83 Δhcp_JP2 |
| RBM19_HUMAN | 0.00 | 3.29 | HK83+JP2 |
| TRM1L_HUMAN | 0.00 | 2.15 | HK83+JP2 |
| P63_HUMAN | 0.01 | 2.45 | HK83+JP2 |
| BYST_HUMAN | 0.00 | 2.88 | HK83+JP2 |
| ZMYM2_HUMAN | 0.00 | 2.14 | HK83+JP2 |
| I2BP2_HUMAN | 0.00 | 2.53 | HK83+JP2 |
| A0A087WWF6_HUMAN | 0.00 | 2.72 | HK83+JP2 |
| RDH13_HUMAN | 0.00 | 2.11 | HK83+JP2 |
| CKS1_HUMAN | 0.00 | 2.55 | HK83+JP2 |
| ICAM1_HUMAN | 0.00 | 3.98 | HK83 Δhcp_JP2 |
| ADDA_HUMAN | 0.00 | 2.22 | HK83+JP2 |
| PHLB1_HUMAN | 0.01 | 2.17 | HK83+JP2 |
| IL1B_HUMAN | 0.00 | 3.86 | HK83 Δhcp_JP2 |
| IL1RA_HUMAN | 0.03 | 2.09 | HK83 Δhcp_JP2 |
| S10A9_HUMAN | 0.00 | 2.01 | HK83 Δhcp_JP2 |
| DAG1_HUMAN | 0.00 | 2.25 | HK83+JP2 |
| CDC20_HUMAN | 0.02 | 3.11 | HK83+JP2 |
| DYR_HUMAN | 0.01 | 2.28 | HK83+JP2 |
| I3L2R9_HUMAN | 0.00 | 2.06 | HK83+JP2 |
| TYSY_HUMAN | 0.00 | 2.30 | HK83+JP2 |
| sp\|O00762\|UBE2C_HUMAN | 0.01 | 2.28 | HK83+JP2 |
| SFR15_HUMAN | 0.04 | 2.61 | HK83 Δhcp_JP2 |
| K2C78_HUMAN | 0.05 | 6.01 | HK83+JP2 |
| I3L0X5_HUMAN | 0.00 | 2.26 | HK83+JP2 |
| BRE1B_HUMAN | 0.01 | 2.97 | HK83+JP2 |
| DSC3_HUMAN | 0.01 | 2.74 | HK83+JP2 |
| ANLN_HUMAN | 0.00 | 2.38 | HK83+JP2 |
| A0A2R8Y653_HUMAN | 0.00 | 2.17 | HK83+JP2 |
| DYL1_HUMAN | 0.01 | 2.48 | HK83+JP2 |
| RL37A_HUMAN | 0.01 | 2.47 | HK83 Δhcp_JP2 |
| MX2_HUMAN | 0.01 | 2.15 | HK83+JP2 |
| SC61B_HUMAN | 0.00 | 2.01 | HK83 Δhcp_JP2 |
| ARGI1_HUMAN | 0.03 | 4.13 | HK83+JP2 |
| A0A1B0GVV3_HUMAN | 0.00 | 3.45 | HK83+JP2 |
| TMED5_HUMAN | 0.00 | 2.41 | HK83 Δhcp_JP2 |
| KIF4A_HUMAN | 0.00 | 2.78 | HK83+JP2 |
| T2FB_HUMAN | 0.00 | 2.33 | HK83+JP2 |
| PIP_HUMAN | 0.01 | 11.32 | HK83 Δhcp_JP2 |
| APLP2_HUMAN | 0.03 | 2.14 | HK83+JP2 |
| MTND_HUMAN | 0.00 | 2.16 | HK83+JP2 |
|  |  |  |  |
| HK83+JP2 VS HK83 Δtle5 Δglh+JP2 | | | |
| TOP accession | Anova (p) | Max fold change | Highest mean condition |
| FADS2_HUMAN | 0.00 | 2.68 | HK83+JP2 |
| UT14A_HUMAN | 0.00 | 3.83 | HK83+JP2 |
| DDX56_HUMAN | 0.00 | 2.93 | HK83+JP2 |
| S38A2_HUMAN | 0.00 | 2.16 | HK83 Δtle5 Δglh+JP2 |
| TSP1_HUMAN | 0.01 | 2.27 | HK83+JP2 |
| FAT2_HUMAN | 0.02 | 2.21 | HK83+JP2 |
| DIAP3_HUMAN | 0.00 | 3.68 | HK83+JP2 |
| IL1A_HUMAN | 0.00 | 4.66 | HK83 Δtle5 Δglh+JP2 |
| RBM19_HUMAN | 0.00 | 5.14 | HK83+JP2 |
| RFC1_HUMAN | 0.02 | 2.46 | HK83+JP2 |
| LARP4_HUMAN | 0.00 | 2.05 | HK83+JP2 |
| B7ZKJ8_HUMAN | 0.00 | 2.35 | HK83 Δtle5 Δglh+JP2 |
| TRM1L_HUMAN | 0.00 | 2.16 | HK83+JP2 |
| P63_HUMAN | 0.00 | 3.00 | HK83+JP2 |
| BYST_HUMAN | 0.00 | 2.27 | HK83+JP2 |
| ZMYM2_HUMAN | 0.00 | 2.23 | HK83+JP2 |
| I2BP2_HUMAN | 0.00 | 2.45 | HK83+JP2 |
| NUSAP_HUMAN | 0.04 | 2.06 | HK83+JP2 |
| CKS1_HUMAN | 0.00 | 2.49 | HK83+JP2 |
| ICAM1_HUMAN | 0.00 | 3.39 | HK83 Δtle5 Δglh+JP2 |
| ADDA_HUMAN | 0.00 | 2.21 | HK83+JP2 |
| IL1B_HUMAN | 0.00 | 4.71 | HK83 Δtle5 Δglh+JP2 |
| IL1RA_HUMAN | 0.02 | 2.42 | HK83 Δtle5 Δglh+JP2 |
| CCNB1_HUMAN | 0.00 | 2.04 | HK83+JP2 |
| RM55_HUMAN | 0.00 | 2.34 | HK83+JP2 |
| PDS5A_HUMAN | 0.00 | 2.16 | HK83+JP2 |
| CSN4_HUMAN | 0.00 | 2.06 | HK83 Δtle5 Δglh+JP2 |
| S10A9_HUMAN | 0.00 | 3.77 | HK83 Δtle5 Δglh+JP2 |
| DAG1_HUMAN | 0.01 | 2.36 | HK83+JP2 |
| CDC20_HUMAN | 0.01 | 3.33 | HK83+JP2 |
| ZNT1_HUMAN | 0.01 | 2.27 | HK83 Δtle5 Δglh+JP2 |
| DYR_HUMAN | 0.01 | 2.17 | HK83+JP2 |
| PAI2_HUMAN | 0.03 | 2.01 | HK83 Δtle5 Δglh+JP2 |
| DNLI1_HUMAN | 0.00 | 2.77 | HK83+JP2 |
| TYSY_HUMAN | 0.00 | 2.56 | HK83+JP2 |
| RIOK1_HUMAN | 0.01 | 2.08 | HK83+JP2 |
| A0A0A0MQX8_HUMAN | 0.00 | 4.10 | HK83+JP2 |
| A6ND22_HUMAN | 0.00 | 2.08 | HK83 Δtle5 Δglh+JP2 |
| THYN1_HUMAN | 0.01 | 2.32 | HK83+JP2 |
| A0A0A0MTJ9_HUMAN | 0.00 | 2.19 | HK83 Δtle5 Δglh+JP2 |
| A0A087WX29_HUMAN | 0.04 | 2.07 | HK83+JP2 |
| DYL1_HUMAN | 0.00 | 3.56 | HK83+JP2 |
| F8VP94_HUMAN | 0.02 | 2.17 | HK83+JP2 |
| RMP_HUMAN | 0.01 | 2.31 | HK83 Δtle5 Δglh+JP2 |
| P20D2_HUMAN | 0.02 | 2.31 | HK83+JP2 |
| RL37A_HUMAN | 0.00 | 3.18 | HK83 Δtle5 Δglh+JP2 |
| B7Z7B0_HUMAN | 0.02 | 2.07 | HK83+JP2 |
| D6REL8_HUMAN | 0.02 | 2.78 | HK83 Δtle5 Δglh+JP2 |
| COMD9_HUMAN | 0.00 | 2.29 | HK83+JP2 |
| HELZ2_HUMAN | 0.02 | 2.68 | HK83 Δtle5 Δglh+JP2 |
| HBA_HUMAN | 0.03 | 2.20 | HK83 Δtle5 Δglh+JP2 |
| IL36G_HUMAN | 0.02 | 2.10 | HK83 Δtle5 Δglh+JP2 |
| RBX1_HUMAN | 0.00 | 2.58 | HK83+JP2 |
| NEP1_HUMAN | 0.01 | 2.99 | HK83+JP2 |
| K7EIY6_HUMAN | 0.00 | 3.71 | HK83+JP2 |
| KIF4A_HUMAN | 0.02 | 2.04 | HK83+JP2 |
| A0A0A6YYF2_HUMAN | 0.01 | 2.02 | HK83 Δtle5 Δglh+JP2 |
| TBC15_HUMAN | 0.00 | 2.85 | HK83+JP2 |
| ANM3_HUMAN | 0.00 | 2.12 | HK83+JP2 |
| EBP_HUMAN | 0.01 | 2.04 | HK83 Δtle5 Δglh+JP2 |
| sp\|P12035\|K2C3_HUMAN | 0.00 | 2.06 | HK83 Δtle5 Δglh+JP2 |
| PIP_HUMAN | 0.03 | 6.42 | HK83 Δtle5 Δglh+JP2 |
| APLP2_HUMAN | 0.04 | 2.04 | HK83+JP2 |
| BOLA1_HUMAN | 0.01 | 2.05 | HK83+JP2 |
| INVO_HUMAN | 0.05 | 2.21 | HK83 Δtle5 Δglh+JP2 |
| H3BNC9_HUMAN | 0.00 | 2.49 | HK83+JP2 |
| NTM1A_HUMAN | 0.00 | 3.39 | HK83+JP2 |

# The first accession for each protein group is used as the top accession, and the full accession and description can be found in the deposited data. Although fold change and t-test p-value <0.05 are commonly used to define significance in studies of this kind, we also provide the results of multiple comparison tests (q-value and power) in the deposited data for evaluating individual proteins.

**Supplementary Table 5:** List of top ten enriched GO-BPnR functions

| Control VS HK83 | |  |  |  |
| --- | --- | --- | --- | --- |
| **geneSet** | **description** | **Regulated /All identified proteins** | **FDR** | **Involved protein** |
| GO:0048871 | multicellular organismal homeostasis | 8/82 | 2.8E-03 | ZG16B; ACTB; ACTG1; CDH3;IL1A;IL1B;KRT1;TP63 |
| GO:1901342 | regulation of vasculature development | 6/45 | 4.4E-03 | DNMT1;IL1A;IL1B;KRT1;SOD2;THBS1 |
| GO:0002526 | acute inflammatory response | 4/19 | 1.4E-02 | ICAM1;IL1A;IL1B;S100A8 |
| GO:0072593 | reactive oxygen species metabolic process | 6/62 | 1.4E-02 | CRYAB;SH3PXD2A;ICAM1;IL1B;SOD2;THBS1 |
| GO:0098742 | cell-cell adhesion via plasma-membrane adhesion molecules | 4/25 | 2.9E-02 | CDH3;FAT2;ICAM1;IL1RN |
| GO:0018149 | peptide cross-linking | 3/12 | 3.8E-02 | KRT10;KRT1;THBS1 |
| GO:2001233 | regulation of apoptotic signaling pathway | 7/115 | 3.8E-02 | ICAM1;IL1A;IL1B;TP63;S100A8;SOD2;THBS1 |
| GO:0070555 | response to interleukin-1 | 4/31 | 4.3E-02 | ICAM1;IL1A;IL1B;IL1RN |
| GO:0002237 | response to molecule of bacterial origin | 5/61 | 6.0E-02 | ICAM1;IL1B;IL1RN;NFKB2;S100A8 |
| GO:0042107 | cytokine metabolic process | 3/16 | 6.3E-02 | IL1A;IL1B;THBS1 |
|  |  |  |  |  |
| Control VS JP2 | |  |  |  |
| **geneSet** | **description** | **Regulated /All identified proteins** | **FDR** | **Involved protein** |
| GO:0043062 | extracellular structure organization | 12/77 | 1.6E-02 | BSG;ALB;DAG1;SH3PXD2A;ICAM1;  ITGA5;LAMB3;LAMC2;NFKB2;  P4HB;PHLDB1;THBS1 |
| GO:1901342 | regulation of vasculature development | 9/45 | 1.6E-02 | GPNMB;EPHA2;FGFBP1;IL1A;  IL1B;ITGA5;RRAS;SOD2;THBS1 |
| GO:0007369 | gastrulation | 7/35 | 7.9E-02 | TXNRD1;DAG1;EPHA2;  IL1RN;ITGA5;LAMB3;PHLDB1 |
| GO:2001233 | regulation of apoptotic signaling pathway | 13/115 | 7.9E-02 | YWHAB;YWHAE;PSEN1;NOL3;  ICAM1;IL1A;IL1B;TP63;P4HB;  S100A8;S100A9;SOD2;THBS1 |
| GO:0002526 | acute inflammatory response | 5/19 | 8.1E-02 | ITIH4;ICAM1;IL1A;IL1B;S100A8 |
| GO:0060326 | cell chemotaxis | 7/40 | 8.1E-02 | EPHA2;IL1B;IL1RN;LYN;S100A8;S100A9;THBS1 |
| GO:0045861 | negative regulation of proteolysis | 10/78 | 8.1E-02 | YWHAE;ITIH4;CRYAB;CSTB;  PSEN1;NOL3;SERPINB2;TRIM21;SMARCC1;THBS1 |
| GO:0051235 | maintenance of location | 9/74 | 1.6E-01 | YWHAB;EHD1;ALB;DAG1;  NOL3;IL1B;LYN;S100A8;S100A9 |
| GO:0052547 | regulation of peptidase activity | 11/105 | 1.6E-01 | YWHAE;ITIH4;CRYAB;CSTB;  NOL3;LYN;TP63;SERPINB2;S100A8;S100A9;THBS1 |
| GO:0098742 | cell-cell adhesion via plasma-membrane adhesion molecules | 5/25 | 1.6E-01 | BSG;FAT2;ICAM1;IL1RN;ITGA5 |
|  |  |  |  |  |
| Control vs HK83 Δhcp | |  |  |  |
| **geneSet** | **description** | **Regulated /All identified proteins** | **FDR** | **Involved protein** |
| GO:0016072 | rRNA metabolic process | 14/104 | 1.5E-02 | NOL6;WDR36;BYSL;DDX56;GAR1  ;RSL1D1;KRR1;LYAR;MPHOSPH10;  NSA2;RRP1;RRP7A;MRPS9;UTP14A |
| GO:0034470 | ncRNA processing | 15/125 | 1.5E-02 | NOL6;WDR36;BYSL;DDX56;GAR1;  RSL1D1;KRR1;LYAR;MPHOSPH10;  NSA2;RRP1;RRP7A;MRPS9;TRMT1L;UTP14A |
| GO:0055123 | digestive system development | 4/11 | 9.5E-02 | CCNB1;HIP1R;TP63;TYMS |
| GO:0002526 | acute inflammatory response | 5/19 | 9.5E-02 | ITIH4;ICAM1;IL1A;IL1B;S100A8 |
| GO:0051302 | regulation of cell division | 5/21 | 1.3E-01 | KIF23;IL1A;IL1B;TP63;RACGAP1 |
| GO:0007568 | aging | 9/79 | 2.8E-01 | OPA1;CRYAB;EIF5A;ICAM1;  RSL1D1;KYNU;TP63;SOD2;TYMS |
| GO:0010975 | regulation of neuron projection development | 9/81 | 2.8E-01 | WDR36;OPA1;HSPA5;CDC20;  CFL1;CRK;DDX56;CRABP2;RTN4IP1 |
| GO:0048871 | multicellular organismal homeostasis | 9/82 | 2.8E-01 | WDR36;SCD;ACTB;ACTG1;  ADD1;IL1A;IL1B;TP63;PIGR |
| GO:0001101 | response to acid chemical | 8/68 | 2.8E-01 | OPA1;SCD;CCNB1;CFL1;  DNMT1;DHFR;ICAM1;TYMS |
| GO:0048285 | organelle fission | 9/87 | 3.4E-01 | OPA1;CCNB1;CDC20;KIF23;  IL1A;IL1B;NUSAP1;RACGAP1;TPX2 |
|  |  |  |  |  |
| Control vs HK83 Δtle5 Δglh | |  |  |  |
| **geneSet** | **description** | **Regulated /All identified proteins** | **FDR** | **Involved protein** |
| GO:0002526 | acute inflammatory response | 5/19 | 1.4E-01 | ITIH4;ICAM1;IL1A;IL1B;S100A8 |
| GO:0072593 | reactive oxygen species metabolic process | 8/62 | 1.6E-01 | GPX1;CRYAB;SH3PXD2A;  HBA1;HBB;ICAM1;IL1B;SOD2 |
| GO:0016072 | rRNA metabolic process | 10/104 | 2.0E-01 | RSL1D1;KRR1;LYAR;EMG1;  NSA2;RRP1;RRP1B;RRP7A;RRS1;UTP14A |
| GO:0034470 | ncRNA processing | 11/125 | 2.0E-01 | RSL1D1;KRR1;LYAR;EMG1;  NSA2;RRP1;RRP1B;RRP7A;  RRS1;TRMT1L;UTP14A |
| GO:0048871 | multicellular organismal homeostasis | 8/82 | 4.2E-01 | GPX1;ZG16B;SCD;ACTB;ACTG1;AZGP1;IL1A;IL1B |
| GO:2001233 | regulation of apoptotic signaling pathway | 9/115 | 7.3E-01 | GPX1;OPA1;PSEN1;NOL3;  ICAM1;IL1A;IL1B;S100A8;SOD2 |
| GO:0045861 | negative regulation of proteolysis | 7/78 | 7.3E-01 | GPX1;DDRGK1;ITIH4;  CRYAB;PSEN1;NOL3;SERPINB2 |
| GO:0015695 | organic cation transport | 2/5 | 7.3E-01 | PSEN1;SLC38A2 |
| GO:0007568 | aging | 7/79 | 7.3E-01 | OPA1;CRYAB;PSEN1;  ICAM1;RSL1D1;NFKB2;SOD2 |
| GO:0046677 | response to antibiotic | 7/82 | 7.7E-01 | GPX1;OPA1;CRYAB;  HBA1;HBB;ICAM1;S100A8 |
|  |  |  |  |  |
| Control vs HK83+JP2 | |  |  |  |
| **geneSet** | **description** | **Regulated /All identified proteins** | **FDR** | **Involved protein** |
| GO:0034109 | homotypic cell-cell adhesion | 3/27 | 3.2E-02 | ACTB;ACTG1;FGB |
| GO:0002764 | immune response-regulating signaling pathway | 4/91 | 3.2E-02 | TNIP1;ACTB;ACTG1;FGB |
| GO:0050817 | coagulation | 3/70 | 2.3E-01 | ACTB;ACTG1;FGB |
| GO:0048871 | multicellular organismal homeostasis | 3/82 | 2.8E-01 | ZG16B;ACTB;ACTG1 |
| GO:0050878 | regulation of body fluid levels | 3/92 | 3.1E-01 | ACTB;ACTG1;FGB |
| GO:0048013 | ephrin receptor signaling pathway | 2/28 | 3.5E-01 | ACTB;ACTG1 |
| GO:0060249 | anatomical structure homeostasis | 3/108 | 3.5E-01 | ZG16B;ACTB;ACTG1 |
| GO:0048880 | sensory system development | 2/42 | 6.2E-01 | HMGN1;CRYAB |
| GO:0070371 | ERK1 and ERK2 cascade | 2/46 | 6.6E-01 | TNIP1;FGB |
| GO:0006909 | phagocytosis | 2/62 | 8.8E-01 | ACTB;ACTG1 |
|  |  |  |  |  |
| Control vs HK83 Δhcp+JP2 | |  |  |  |
| **geneSet** | **description** | **Regulated /All identified proteins** | **FDR** | **Involved protein** |
| GO:0007568 | aging | 9/79 | 0.01 | OPA1;CRYAB;DAG1;ICAM1;  RSL1D1;KYNU;NFKB2;TP63;SOD2 |
| GO:0002526 | acute inflammatory response | 5/19 | 0.01 | ITIH4;ICAM1;IL1A;IL1B;S100A8 |
| GO:0098742 | cell-cell adhesion via plasma-membrane adhesion molecules | 5/25 | 0.02 | CDH3;DSC3;FAT2;ICAM1;IL1RN |
| GO:0045995 | regulation of embryonic development | 4/23 | 0.15 | DAG1;IL1RN;PHLDB1;RBM19 |
| GO:0070555 | response to interleukin-1 | 4/31 | 0.37 | ICAM1;IL1A;IL1B;IL1RN |
| GO:0021700 | developmental maturation | 4/35 | 0.37 | OPA1;CDH3;CDC20;DAG1 |
| GO:0006022 | aminoglycan metabolic process | 3/18 | 0.37 | ITIH4;GPC1;IL1B |
| GO:0010927 | cellular component assembly involved in morphogenesis | 3/18 | 0.37 | DAG1;GPC1;PHLDB1 |
| GO:0030856 | regulation of epithelial cell differentiation | 3/19 | 0.37 | ADD1;IL1B;TP63 |
| GO:2001233 | regulation of apoptotic signaling pathway | 7/115 | 0.37 | OPA1;ICAM1;IL1A;IL1B;TP63;S100A8;SOD2 |
|  |  |  |  |  |
| Control vs HK83 Δtle5 Δglh +JP2 | | |  |  |
| **geneSet** | **description** | **Regulated /All identified proteins** | **FDR** | **Involved protein** |
| GO:0002526 | acute inflammatory response | 5/19 | 0.11 | ITIH4;ICAM1;IL1A;IL1B;S100A8 |
| GO:0072593 | reactive oxygen species metabolic process | 8/62 | 0.11 | CRYAB;SH3PXD2A;HBA1;  HBB;ICAM1;IL1B;SOD2;THBS1 |
| GO:0007568 | aging | 9/79 | 0.11 | CRYAB;DAG1;PSEN1;ICAM1;  RSL1D1;NFKB2;TP63;SOD2;TYMS |
| GO:0016072 | rRNA metabolic process | 10/104 | 0.15 | BYSL;DDX56;RSL1D1;KRR1;  NIFK;EMG1;NSA2;RIOK1;RRP1;UTP14A |
| GO:0070555 | response to interleukin-1 | 5/31 | 0.25 | ICAM1;IL1A;IL1B;IL1RN;RBX1 |
| GO:0030856 | regulation of epithelial cell differentiation | 4/19 | 0.25 | ADD1;CDSN;IL1B;TP63 |
| GO:0034470 | ncRNA processing | 10/125 | 0.31 | BYSL;DDX56;RSL1D1;KRR1;  EMG1;NSA2;RIOK1;RRP1;TRMT1L;UTP14A |
| GO:0050817 | coagulation | 7/70 | 0.31 | ACTB;ACTG1;PSEN1;  HBB;SERPINB2;PTPN6;THBS1 |
| GO:0045995 | regulation of embryonic development | 4/23 | 0.31 | DAG1;IL1RN;PHLDB1;RBM19 |
| GO:0002064 | epithelial cell development | 6/54 | 0.31 | ADD1;CDSN;ICAM1;IL1B;TP63;TYMS |
|  |  |  |  |  |
| HK83 vs HK83 Δhcp | |  |  |  |
| **geneSet** | **description** | **Regulated /All identified proteins** | **FDR** | **Involved protein** |
| GO:0046939 | nucleotide phosphorylation | 2/29 | 1.00 | PRKAG1;DHTKD1 |
| GO:0009132 | nucleoside diphosphate metabolic process | 2/35 | 1.00 | PRKAG1;DHTKD1 |
| GO:0051291 | protein heterooligomerization | 2/39 | 1.00 | NUP58;KRT10 |
| GO:0006090 | pyruvate metabolic process | 2/40 | 1.00 | PRKAG1;DHTKD1 |
| GO:0016053 | organic acid biosynthetic process | 3/110 | 1.00 | PRKAG1;DHTKD1;FADS2 |
| GO:0016052 | carbohydrate catabolic process | 2/41 | 1.00 | PRKAG1;DHTKD1 |
| GO:0016358 | dendrite development | 2/44 | 1.00 | CDC20;RTN4IP1 |
| GO:0051262 | protein tetramerization | 2/48 | 1.00 | NUP58;KRT10 |
| GO:1902850 | microtubule cytoskeleton organization involved in mitosis | 2/48 | 1.00 | CDC20;KIF23 |
| GO:0007051 | spindle organization | 2/52 | 1.00 | CDC20;KIF23 |
|  |  |  |  |  |
| HK83 vs HK83 Δtle5 Δglh | |  |  |  |
| **geneSet** | **description** | **Regulated /All identified proteins** | **FDR** | **Involved protein** |
| GO:0043062 | extracellular structure organization | 3/77 | 1.00 | TGFBI;PXDN;THBS1 |
| GO:0045861 | negative regulation of proteolysis | 3/78 | 1.00 | DDRGK1;UCHL5;THBS1 |
| GO:0061448 | connective tissue development | 2/28 | 1.00 | DDRGK1;TGFBI |
| GO:0052547 | regulation of peptidase activity | 3/105 | 1.00 | DDRGK1;UCHL5;THBS1 |
| GO:0007162 | negative regulation of cell adhesion | 2/47 | 1.00 | TGFBI;THBS1 |
| GO:0021591 | ventricular system development | 1/6 | 1.00 | UCHL5 |
| GO:0072593 | reactive oxygen species metabolic process | 2/62 | 1.00 | PXDN;THBS1 |
| GO:0032615 | interleukin-12 production | 1/8 | 1.00 | THBS1 |
| GO:0033555 | multicellular organismal response to stress | 1/8 | 1.00 | THBS1 |
| GO:0019882 | antigen processing and presentation | 2/70 | 1.00 | KIF4A;THBS1 |
|  |  |  |  |  |
| HK83+JP2 vs HK83 Δhcp+JP2 | |  |  |  |
| **geneSet** | **description** | **Regulated /All identified proteins** | **FDR** | **Involved protein** |
| GO:0048285 | organelle fission | 8/87 | 0.09 | ANLN;CDC20;NDE1;IL1A;IL1B;KIF4A;MX2;UBE2C |
| GO:0002064 | epithelial cell development | 6/54 | 0.12 | RILPL1;ADD1;ICAM1;IL1B;TP63;TYMS |
| GO:0045995 | regulation of embryonic development | 4/23 | 0.16 | DAG1;IL1RN;PHLDB1;RBM19 |
| GO:0098742 | cell-cell adhesion via plasma-membrane adhesion molecules | 4/25 | 0.16 | DSC3;FAT2;ICAM1;IL1RN |
| GO:0044772 | mitotic cell cycle phase transition | 8/129 | 0.25 | ANLN;CDC20;CKS1B;DYNLL1;  DHFR;NDE1;TYMS;UBE2C |
| GO:0070555 | response to interleukin-1 | 4/31 | 0.25 | ICAM1;IL1A;IL1B;IL1RN |
| GO:0051258 | protein polymerization | 6/84 | 0.32 | ADD1;DIAPH3;NDE1;ICAM1;MX2;UBE2C |
| GO:0002526 | acute inflammatory response | 3/19 | 0.32 | ICAM1;IL1A;IL1B |
| GO:0030856 | regulation of epithelial cell differentiation | 3/19 | 0.32 | ADD1;IL1B;TP63 |
| GO:0048678 | response to axon injury | 3/19 | 0.32 | ARG1;DAG1;DHFR |
|  |  |  |  |  |
| HK83+JP2 vs HK83 Δtle5 Δglh +JP2 | | |  |  |
| **geneSet** | **description** | **Regulated /All identified proteins** | **FDR** | **Involved protein** |
| GO:0070555 | response to interleukin-1 | 6/31 | 0.06 | FGB;ICAM1;IL1A;IL1B;IL1RN;RBX1 |
| GO:0002526 | acute inflammatory response | 4/19 | 0.28 | ITIH4;ICAM1;IL1A;IL1B |
| GO:0009612 | response to mechanical stimulus | 5/34 | 0.28 | CCNB1;DAG1;IL1B;SLC38A2;THBS1 |
| GO:0055123 | digestive system development | 3/11 | 0.28 | CCNB1;TP63;TYMS |
| GO:0045995 | regulation of embryonic development | 4/23 | 0.28 | LAMA3;DAG1;IL1RN;RBM19 |
| GO:0060326 | cell chemotaxis | 5/40 | 0.28 | IL1B;IL1RN;IL36G;S100A9;THBS1 |
| GO:0042558 | pteridine-containing compound metabolic process | 3/12 | 0.28 | DHFR;PM20D2;TYMS |
| GO:0031099 | regeneration | 5/44 | 0.29 | CCNB1;DAG1;DHFR;TARBP2;TYMS |
| GO:0048285 | organelle fission | 7/87 | 0.29 | CCNB1;CDC20;IL1A;IL1B;KIF4A;NUSAP1;PDS5A |
| GO:0010038 | response to metal ion | 7/89 | 0.29 | ADD1;CCNB1;FGB;ICAM1;IL1A;THBS1;SLC30A1 |

**Supplementary Table 6:** List of protein-protein interactions between two immuno-related GO terms

| #node1 | node2 | combined_score |
| --- | --- | --- |
| ACTB | IL1B | 0.847 |
| ACTB | ACTG1 | 0.999 |
| ACTG1 | ACTB | 0.999 |
| ICAM1 | IL1A | 0.807 |
| ICAM1 | IL1B | 0.912 |
| IL1A | IL1RN | 0.99 |
| IL1A | ICAM1 | 0.807 |
| IL1A | IL1B | 0.999 |
| IL1B | IL1RN | 0.968 |
| IL1B | IL1A | 0.999 |
| IL1B | ACTB | 0.847 |
| IL1B | ICAM1 | 0.912 |
| IL1RN | IL1B | 0.968 |
| IL1RN | IL1A | 0.99 |
